# Circular RNA CDR1as Alleviates Cisplatin-Based Chemoresistance by Suppressing MiR-1299 in Ovarian Cancer

**DOI:** 10.3389/fgene.2021.815448

**Published:** 2022-01-26

**Authors:** Han Wu, Xibo Zhao, Jing Wang, Xinyan Jiang, Yan Cheng, Yanan He, Liyuan Sun, Guangmei Zhang

**Affiliations:** Department of Gynecology, The First Affiliated Hospital, Harbin Medical University, Harbin, China

**Keywords:** ovarian cancer, cisplatin chemoresistance, CDR1as, miR-1299, PPP1R12B

## Abstract

Cisplatin (CDDP) chemoresistance seriously affects the prognosis and survival of patients with ovarian cancer (OC). Previous research has shown that circular RNA CDR1as is biologically associated with a large number of cancers. However, the molecular mechanism underlying the role of CDR1as in CDDP chemoresistance in OC remains unclear. Here, we investigated the mechanism of CDR1as in CDDP-resistant OC. First, we employed bioinformatics analysis and quantitative real-time PCR (qRT-PCR) to determine the expression of CDR1as and related RNAs in CDDP-sensitive and -resistant OC tissues and cells. Then, functional experiments were used to determine cell proliferation, invasion, migration, and apoptosis in CDDP chemoresistance and parent OC cells *in vitro*. The effect of CDR1as in CDDP chemoresistance OC progression was tested in nude mice *in vivo*. Moreover, dual-luciferase assays and RNA immunoprecipitation (RIP) were performed to confirm the interactions of CDR1as and related RNAs. Finally, we used Western blotting to determine protein expression levels. Our findings interpret the underlying mechanisms of the CDR1as/miR-1299/PPP1R12B axis and shed light on the clinical applications for CDDP-chemoresistant OC.

## Introduction

Ovarian cancer (OC) is one of the most common malignant tumors of the female reproductive organs and the second most common form of cancer, followed by cervical cancer and uterine body cancer. OC is considered to be the most fatal form of cancer in gynecological oncology with the highest mortality rate (Barani et al., 2021). Due to the lack of sensitive diagnosis and specific symptoms, OC patients are difficult to identify in the early stage of disease. Surgical resection and cisplatin-based chemotherapy still serve as the main treatments for OC. However, 50% of OC patients acquire drug resistance after several cycles of cisplatin (CDDP) chemotherapy. The 5-year survival rate ranges from 25% to 30% in OC (Long et al., 2019). Therefore, it is necessary to further investigate the underlying molecular mechanisms of carcinogenesis and CDDP chemoresistance in OC so that we can improve the prognosis and survival of patients with advanced OC.

Circular RNAs (circRNAs) are a new form of non-coding RNAs (ncRNAs) that lack both a 5′-cap structure and a 3′-poly-A tail; these are considered to represent by-products of aberrant splicing (Huang et al., 2020). circRNAs can regulate transcription and function as competitive endogenous RNAs (ceRNAs) to sponge microRNAs (miRNAs) and suppress their expression (Chen, 2016). circRNAs are widely expressed in mammalian cells and differ across diverse tissues and cell lines. Circular RNA hsa_circ_0001946, also known as cerebella degeneration-associated protein 1 antisense transcript (CDR1as) or ciRS-7, contains more than 70 binding sites for miR-7 (Hansen et al., 2013). CDR1as is located on the X chromosome (chrX: 139865339–139866824) and is 1,485 bp in size. CDR1as has been discovered in multiple tumors and participates in various biological processes, such as proliferation, invasion, and migration (Jian et al., 2020). In glioma, CDR1as can disrupt the complex of p53 and MDM2 to inhibit tumorigenesis (Lou et al., 2020). CDR1as can sponge miR-1270 to dysregulate AFP levels and promote tumor progression in hepatocellular carcinoma (Su et al., 2019) and can also enhance E2F3 expression to promote nasopharyngeal carcinoma by sponging miR-7-5p (Zhong et al., 2019). However, the specific relationship between CDR1as and CDDP resistance in OC remains unknown.

MicroRNAs (miRNAs) are a class of small non-coding RNA molecules that contain 21–25 nucleotide RNAs. miRNAs can play a critical role in gene expression and cell differentiation and bind to target mRNAs to cause the inhibition of mRNA translation or degradation (Rupaimoole and Slack, 2017). miR-1299 is known to regulate the occurrence of cancers and play different roles. It has also been reported that miR-1299 is involved in the paclitaxel resistance of OC (Zhao et al., 2020). Nevertheless, the exact role of miR-1299 in CDDP chemoresistant OC remains unknown. Therefore, we aimed to investigate the regulatory mechanism and potential function of CDR1as and miR-1299 in CDDP chemoresistant OC.

The protein phosphatase 1 regulatory subunit 12B gene (PPP1R12B), also referred to as MYPT2, is a member of the KARPP-32 family. PPP1R12B mRNA has been confirmed to be involved in dimerization and protein–protein interaction (Grassie et al., 2011). Through bioinformatic analysis (Diana, TargetMiner, TargetScan, miRDB), we found complementary binding sequences between miR-1299 and PPP1R12B. However, thus far, PPP1R12B has yet to be the main focus of any study on OC.

In this study, we investigated the effects of CDR1as and demonstrated that CDR1as enhances CDDP sensitivity to OC and acts as a sponge for miR-1299 *via* the upregulation of PPP1R12B. Our findings may be beneficial to the treatment of OC patients with CDDP chemoresistance and provide a basis for investigating the diagnostic and therapeutic values of the molecular mechanisms involved in CDDP-resistant OC.

## Materials and Methods

### Microarray and RNA-Seq Analyses *In Silico*

Raw data of GSE45553 and GSE51683 with the same platform were obtained from GEO database (https://www.ncbi.nlm.nih.gov/geo/). Samples in either dataset were selected and grouped by either resistance to CDDP or not. The robust multichip average preprocessing methodology and nearest neighbor averaging strategy were applied using “oligo” (Carvalho et al., 2007) and “impute” (Troyanskaya et al., 2001) packages, followed by batch effect removal using “Combat” (Johnson et al., 2007) function for processing the raw data and accurate integrating analysis. All circRNA transcripts from circBase being downloaded (Glazar, et al., 2014), a re-annotation method was performed using the SeqMap tool to map probes to circRNA transcripts without mismatches (Jiang and wong, 2008; Du et al., 2013). Meanwhile, the reference genome (hg19) from the UCSC genome browser (https://genome.ucsc.edu/) and comprehensive gene annotation from GENCODE (v38lift37 version) (https://www.gencodegenes.org/) were downloaded to discard the probes that mapped to other transcripts, retaining ones that re-annotated to circRNAs uniquely. Duplicated circRNAs with the same probe ID were merged by their arithmetic mean values. Differentially expressed circRNAs were screened out using the “limma” package with thresholds set as adjusted *p*-value < 0.05 and | log_2_-fold change (FC)| > 1 (Jiang and Wong, 2008). Also, the mRNA-sequencing profiles (level 3) were downloaded from The Cancer Genome Atlas (TCGA), while the RNA-seq datasets of normal ovarian tissue were separated from the Genotype-Tissue Expression (GTEx) (Carithers et al., 2015).

### Tissues

Six pairs of ovarian cancer tissues and adjacent normal tissues were obtained from the Department of Obstetrics and Gynecology in the First Affiliated Hospital of Harbin Medical University between 2017 and 2019. All the patients had received radical surgical resection without chemotherapy or radiotherapy prior to surgery. The samples were stored at −80°C. The collection of human samples was approved by the Biomedical Ethics Committee of Harbin Medical University First Affiliated Hospital (Ethics number 2020JS20).

### Cell Culture and Treatment

We purchased a human OC cell line SKOV3 and CDDP-resistant strains (SKOV3/CDDP) from the American Type Culture Collection (ATCC, United States). We also purchased the human OC cell line HO8910 and CDDP-resistant strains (HO8910/CDDP) from the Cell Bank of the Chinese Academy of Sciences (Shanghai, China). The SKOV3, SKOV3/CDDP, and HO8910/CDDP cell lines were maintained in Roswell Park Memorial Institute 1640 (RPMI-1640) culture medium (Sigma, St. Louis, MO, USA). HO8910 and 293T cells were maintained in Dulbecco’s modified Eagle’s medium (DMEM) (Sigma, USA) with high glucose. All cells were cultured with 10% fetal bovine serum (FBS) (Ausbian, Graz, Austria) and 1% penicillin–streptomycin (100 IU/ml) at 37°C with 5% CO_2_.

### RNA Extraction and Quantitative Real-Time PCR

Total RNAs (circRNAs, miRNAs, and mRNAs) were isolated from tissues and cells using TRIzol reagent (Invitrogen, Carlsbad, CA, USA) in accordance with the manufacturer’s protocol. mRNA was reversely transcribed into complementary DNA (cDNA) using a PrimeScript RT Reagent Kit (TaKaRa, Shiga, Japan). circRNA and miRNA were directly reverse transcribed using the Bulge-Loop miRNA qRT-PCR Starter Kit (RiBoBio, Guangzhou, China). qRT-PCR assays were carried out with the Power SYBR Green PCR Mix and an Agilent Mx3000P PCR system (Stratagene, La Jolla, CA, USA). The expression levels of circRNAs and mRNAs were normalized by GAPDH or β-actin while those of miRNAs were normalized by U6. The CT value was measured, and the 2^−∆∆Ct^ method was used to analyze the results. All experiments were performed in triplicate. The primer sequences are listed in Table 1.

**TABLE 1:** Primer sequences for real-time PCR.

Gene	Primer sequences (5′–3′)
CDR1as	Forward: ACC​CAG​TCT​TCC​ATC​AAC​TGG
	Reverse: TTG​ACA​CAG​GTG​CCA​TCG​GA
miR-1299	Forward: GGG​AAA​TCG​TGC​GTG​ACA​T
	Reverse: CTG​GAA​GGT​GGA​CAG​CGA​G
PPP1R12B	Forward: ATC​ACG​GAG​CCA​GTG​TAG​GTA​TT
	Reverse: GCC​TGC​CTC​ACA​TCC​TCT​ATT​TT
β-Actin	Forward: CTC​CAT​CCT​GGC​CTC​GCT​GT
	Reverse: GCT​GCT​ACC​TTC​ACC​GTT​CC
GAPDH forward	TGC​ACC​ACC​AAC​TGC​TTA​GC
	Reverse: GGC​ATG​GAC​TGT​GGT​CAT​GAG
U6	Forward: GGA​ACG​ATA​CAG​AGA​AGA​TTA​GCA
	Reverse: GTGCAGGGTCCGAGGT

### Cell Transfection

We purchased a CDR1as knockdown lentivirus (including sh-RNA1, sh-RNA2, and sh-RNA3), a negative control lentivirus (sh-Control), a CDR1as overexpression lentivirus (h-CDR1as), and a control lentivirus (h-Control), from Hanbio Biotechnology (Shanghai, China). miR-1299 mimics and controls were synthesized by GenePharma (Shanghai, China). The transfection was carried out using Lipofectamine 2000 reagent (Thermo Fisher Scientific, Waltham, MA, USA), and 293T cells were cultured in 12-well plates (5 × 10^4^/well). Cells were harvested for 48 h after transfection. Stable transfected cell lines were then selected with puromycin for 7–10 days. The sequences of CDR1as knockdown lentivirus are listed in Supplementary Table 1.

### CCK-8 Assay

Cell proliferation was determined with a Cell Counting Kit-8 assay (Beyotime, Shanghai, China). Cells were seeded into 96-well plates (Corning, NY, USA) (1 × 10^3^/well), and 10 μl of CCK-8 solution was added to each well at three collection times (24, 48, and 72 h). After 30 min of incubation at 37°C, the absorbance of each well was measured at 450 nm using a Microplate Reader ELx800 (BioTek Instruments Inc., Highland Park Winooski, VT, USA). The CCK-8 assays were carried out in triplicate.

### 5-Ethynyl-2′-Deoxyuridine Assay

We used a 5-ethynyl-2′-deoxyuridine (EdU) assay kit (RiboBio, Guangzhou, China) to perform EdU assays. We seeded 1 × 10^4^ cells into 96-well plates for overnight incubation. On the second day, we added 100 μl of EdU solution (50 μM) into the cells and incubated it for 2 h. Then, the cells were fixed with 4% paraformaldehyde for 30 min. Then, we added 100 μl of Apollo Reaction Solution to the wells and incubated it for 30 min; this was followed by incubation with 100 μl of Hoechst 33342 in the dark for 30 min. Finally, the cells were photographed with an EVOS M5000 Inverted Fluorescence Microscope (Thermo Fisher Scientific, USA).

### Cell Apoptosis Assay

Cell apoptosis assays were carried out with an Annexin V-allophycocyanin (APC)/propidium iodide (PI) apoptosis kit (MULTI SCIENCES, Hangzhou, China) in accordance with the manufacturer’s protocol. Then, 1 × 10^6^ cells were seeded and washed twice with phosphate balanced solution (PBS). Then, cells were suspended in 500 μl of 1× binding buffer. The cells were then stained using 5 μl of Annexin V-APC and 10 μl of PI for 5 min in the dark at room temperature. The samples were measured by an Apogee A50-Micro Flow cytometer (Apogee, Kent, UK).

### Wound Healing Assay

Cells were cultured in 6-well plates for wound healing assays. When cell confluency reached 80%–90%, the cells were scratched with a 200-μl plastic pipette tip in the middle of the 6-well plates. Then, the cells were washed with PBS to remove the cell debris. Then, we added medium (without FBS) to the 6-well plates for 24 h. Images of wound healing were acquired by microscopy at 0 and 24 h. ImageJ software was used to analyze the width of the wound.

### Transwell Assay

Transwell chambers (Corning Costar, Cambridge, MA, USA, 8.0 μm pore size) were used to determine the extent of cell migration. The upper chamber was covered with 45 μl of Matrigel (BD Biosciences, San Jose, CA, USA). We then seeded the upper chamber with 1 × 10^5^ cells in 200 μl of medium (without FBS). Then, 550 μl of medium containing 20% FBS was loaded into the lower chamber. After 24 h, the surface cells in the upper chamber were wiped softly with cotton swabs. Cells on the lower side of the upper chamber were fixed with 4% paraformaldehyde for 1.5 h and then stained with crystal violet solution for 30 min at room temperature. Images were then acquired by an Inverted Fluorescence Microscope (Carl Zeiss, Germany). Cell numbers were determined and compared using ImageJ software. Experiments were repeated at least three times.

### Dual-Luciferase Reporter Assay

First, we seeded 293T cells into 24-well plates. Then, we used Lipofectamine 2000 (Invitrogen, Carlsbad, CA, USA) for transfection. We co-transfected cells with CDR1as and PPP1R12B wild-type or mutant plasmids (CDR1as-wt, CDR1as-mut, PPP1R12B-wt, PPP1R12B-mut) and miR-1299 mimic or miR-NC. After 48 h, luciferase activity was measured using a Luciferase Reporter Assay System (Promega, WI, USA) according to the manufacturer’s instructions. The plasmids were obtained from Hanbio Biotechnology (Shanghai, China). All experiments were carried out in triplicate.

### RNA Immunoprecipitation Assay

RIP assays were carried out with a Magna RNA Immunoprecipitation (RIP) Kit (Millipore, Bedford, MA, USA) in accordance with the manufacturer’s protocol. The AGO2 and IgG antibodies used for RIP assays were purchased from Abcam (Cambridge, MA, USA). First, we cultured 1 × 10^7^ SKOV3 or HO8910 cells in 1 ml of PBS. Then, the cell lysates were incubated with RIP buffer on ice for 30 min. The cell lysates were immunoprecipitated with anti-AGO2 or anti-IgG with protein A/G magnetic beads. Magnetic bead-bound complexes were immobilized with magnet, and unbound materials 10–15 times were washed off with PBS. The complex bound to the magnetic beads was eluted, and the RNAs are extracted. Finally, the immunoprecipitated RNAs were detected by qRT-PCR.

### Western Blotting

Cells were seeded into 1 ml of PBS. Total proteins were then lysed with RIPA lysis buffer containing protease inhibitors. Then, 20 μg of total protein (per sample) was separated by sodium dodecyl sulfate-polyacrylamide gel electrophoresis (SDS-PAGE) and then transferred onto a polyvinylidene fluoride (PVDF) membrane (Millipore, USA). The membranes were incubated with specific primary antibodies (anti-PPP1R12B, anti-Akt, anti-p-Akt, anti-mTOR, anti-p-mTOR, anti-GAPDH, or anti-β-actin) (Abcam, USA) overnight at 4°C. Then, the membranes were washed thrice with TBST buffer and then incubated with appropriate secondary antibodies (anti-rabbit/mouse IgG) (CST, Danvers, MA, USA) for 2 h in the dark. Positive signals were then detected using a chemiluminescence detection system (Applygen, Beijing, China). GAPDH and β-actin were used as loading controls.

### Tumor Xenograft Study

Twelve BALB/c nude female mice at 4 weeks of age were purchased from Charles River Laboratories (Beijing, China). In order to prevent the murine stress response, we housed mice with free access to food and water for 1 week in specific pathogen-free conditions. Then, we suspended 5 × 10^6^ SKOV3 cells that were stably transfected with knockdown CDR1as lentivirus or control lentivirus in 100 μl of cold PBS and injected this solution into the dorsal flanks of the nude mice. After 1 week, we divided the mice randomly into a treatment group and a control group. Mice in the treatment group were injected with CDDP (5 mg/kg) twice a week; mice in the control groups were injected with the same dose of PBS. Twenty-eight days later, the mice were sacrificed to collect tumors and calculate their size. Tumor volume was estimated by the following formula: volume = 0.5 × length × width^2^. Animal experiments took place in the Animal Experiment Center, Harbin Veterinary Research Institute, Chinese Academy of Agricultural Sciences. This study was carried out according to the Guidelines for the Care and Use of Laboratory Animals and was approved by the Institutional Animal Care and Use Committee of Harbin Medical University First Affiliated Hospital.

### Statistical Analysis

GraphPad Prism version 9.0 and Statistical Product and Service Solutions (SPSS) were used for statistical analysis. Results are presented as the mean value ± standard deviation (SD). The Student’s *t*-test and one-way analysis of variance (ANOVA) were used to compare two or more groups. *p* < 0.05 was considered statistically significant.

## Results

### CDR1as Was Downregulated in OC and Associated With CDDP Chemoresistance

**FIGURE 1:**
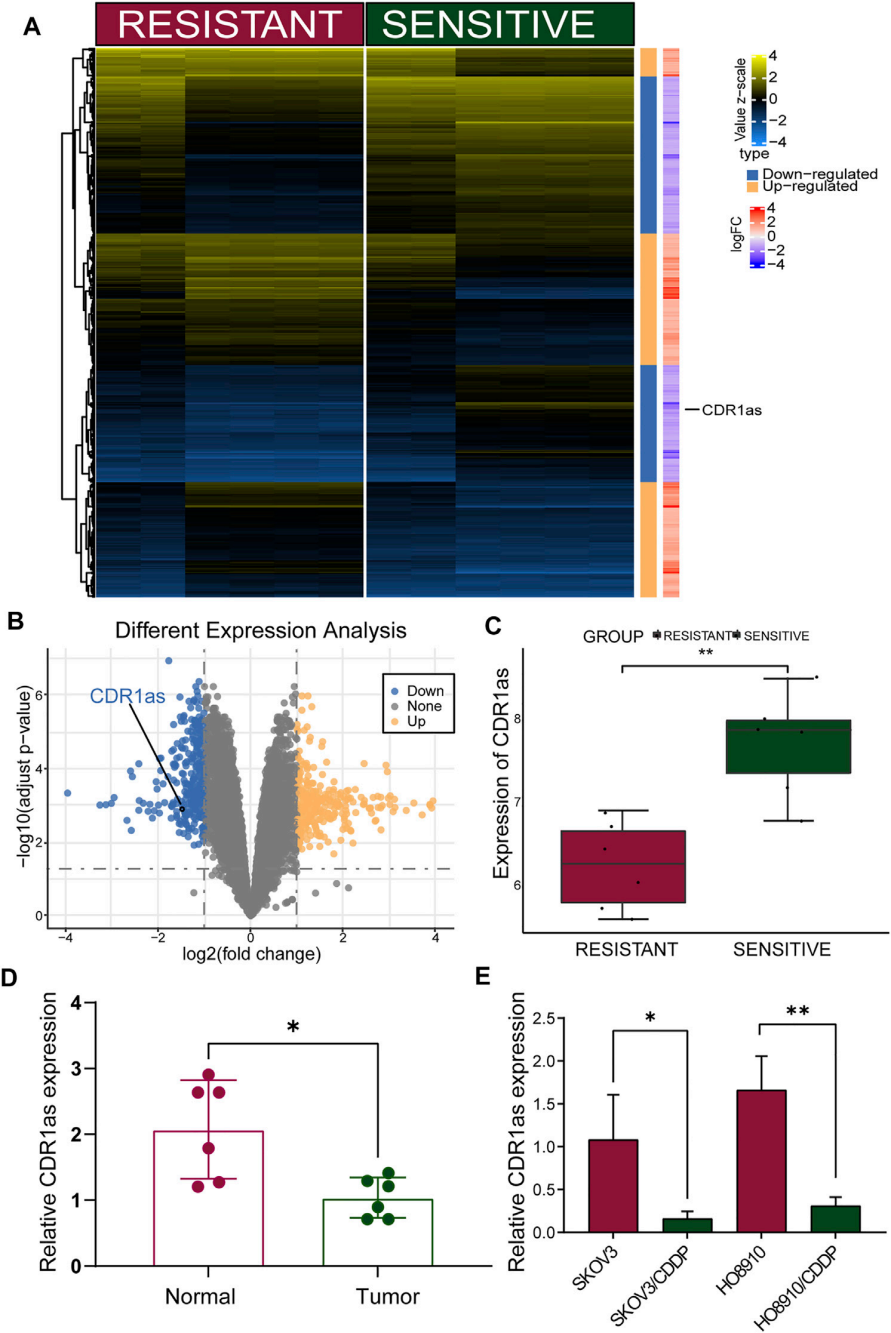
Circular RNA CDR1as significant downregulated in OC resistant tissues and cell lines. **(A,B)** The heatmap and volcano plot show variation in circRNAs expression between CDDP-resistant and -sensitive OC cell lines. **(C)** The expression of CDR1as in CDDP-resistant and -sensitive cell lines by bioinformatics analysis. **(D)** Expression levels of CDR1as in OC tissues and their adjacent normal tissues by qRT-PCR. **(E)** qRT-PCR of CDR1as expression in CDDP resistant OC cell lines (SKOV3/CDDP and HO8910/CDDP) and the parental cell lines (SKOV3 and HO8910). These results were presented as the mean ± SDs. *p < 0.05, **p < 0.01, ***p < 0.001 and ****p < 0.0001.

First, we re-annotated probes and detected the expression of CDR1as in two microarray datasets (Supplementary Figure 1). We created a hierarchical clustering heatmap showing all differentially expressed circRNAs (Figure 1A). Compared with the CDDP-sensitive group, the levels of CDR1as were reduced in the CDDP-resistant group (Figures 1B,C). Subsequently, we confirmed the expression of CDR1as in OC and adjacent normal tissues by qRT-PCR analysis. As shown in Figure 1D, CDR1as was expressed at significantly higher expression levels in non-tumor (normal) tissues compared to OC tissues. Furthermore, we also verified the expression of CDR1as in OC cell lines by qRT-PCR. The expression levels of CDR1as showed a reducing trend decreasing from CDDP-sensitive OC cells (SKOV3 and HO8910) to CDDP-resistant OC cells (SKOV3/CDDP and HO8910/CDDP) (Figure 1E). These results showed that CDR1as was highly expressed in CDDP-resistant OC tissues and cell lines and may be related to the promotion of CDDP chemoresistance in OC.

### CDR1as Inhibited CDDP Chemoresistance in OC *In Vitro*

**FIGURE 2:**
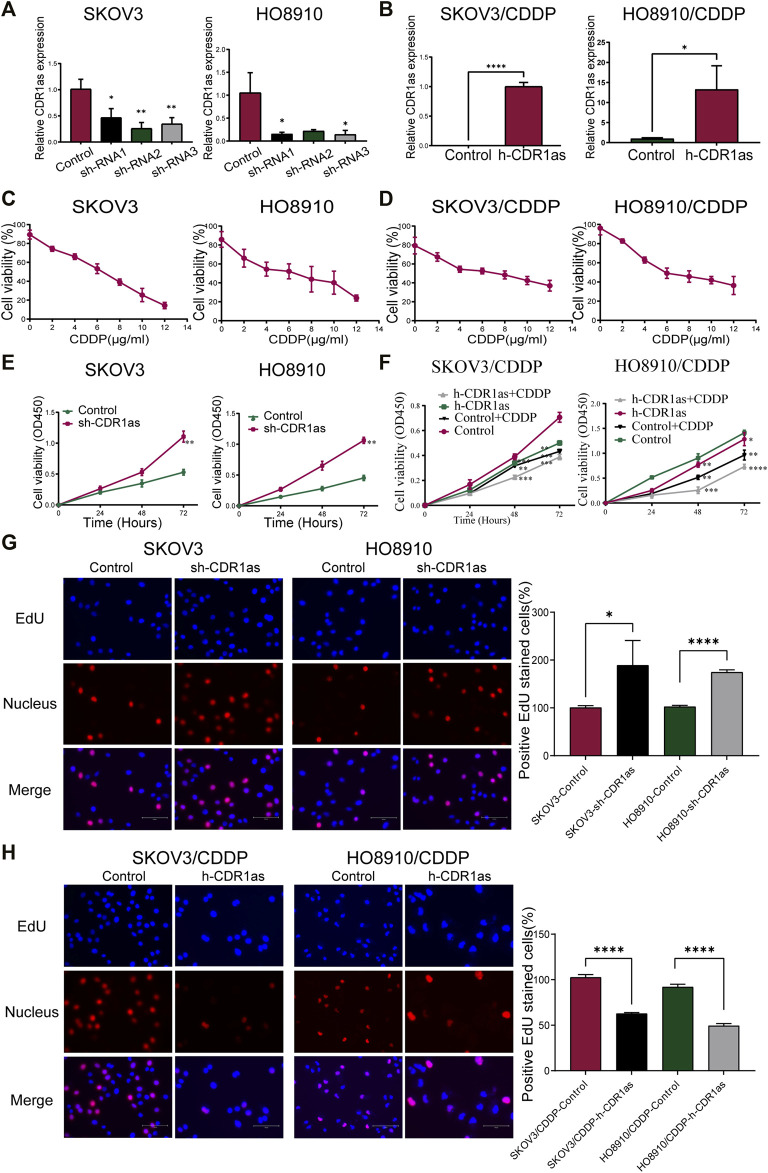
CDR1as suppresses the cell proliferation *in vitro*. **(A,B)** Verification the efficiency of downregulated CDR1as in SKOV3 and HO8910 cells **(A)** and overexpressed CDR1as in SKOV3/CDDP and HO8910/CDDP cells **(B)** by qRT-PCR. **(C,D)** MTT determined the IC50 of CDDP. **(E,F)** CCK-8 assays detected the cell proliferation of CDDP-sensitive **(E)** and -resistant OC cells **(F)** in the presence of CDDP. **(G,H)** EdU assays were performed to identify the cell proliferation of transfected CDDP-sensitive **(G)** and -resistant OC cells **(H)**. These results were presented as the mean ± SDs. *p < 0.05, **p < 0.01, ***p < 0.001 and ****p < 0.0001.

To explore the role of CDR1as, we conducted loss- and gain-of-function studies in CDDP-sensitive and -resistant OC cells. First, we constructed three types of CDR1as knockdown lentiviruses (sh-RNA1, sh-RNA2, sh-RNA3) and a control lentivirus (sh-Control). The transfection efficiency was determined by qRT-PCR. sh-RNA2 successfully knocked down CDR1as in SKOV3 cells when compared to sh-RNA1 and sh-RNA3, while the HO8910 cells were successfully knocked down by sh-RNA3 (Figure 2A). In addition, the CDDP-resistant cells (SKOV3/CDDP and HO8910/CDDP) were transfected with a CDR1as overexpression lentivirus (h-CDR1as) or control lentivirus (h-Control). qRT-PCR was used to verify the efficiency of transfection (Figure 2B). Subsequently, we constructed stable transfected cell lines (SKOV3-sh-CDR1as, SKOV3-Control, SKOV3/CDDP-h-CDR1as, SKOV3/CDDP-Control, HO8910-sh-CDR1as, HO8910-Control, HO8910/CDDP-h-CDR1as, and HO8910/CDDP-Control).

**FIGURE 3:**
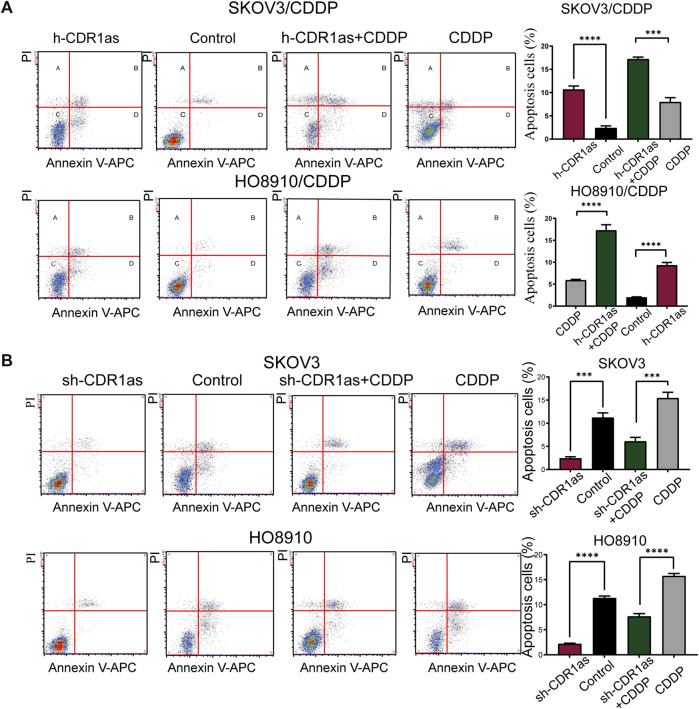
CDR1as promotes the cell apoptosis. **(A,B)** The cell apoptosis rates of the CDDP-resistant and -sensitive OC cells transfected with CDR1as were determined by flow cytometric analysis. These results were presented as the mean ± SDs. ***p < 0.001 and ****p < 0.0001.

**FIGURE 4:**
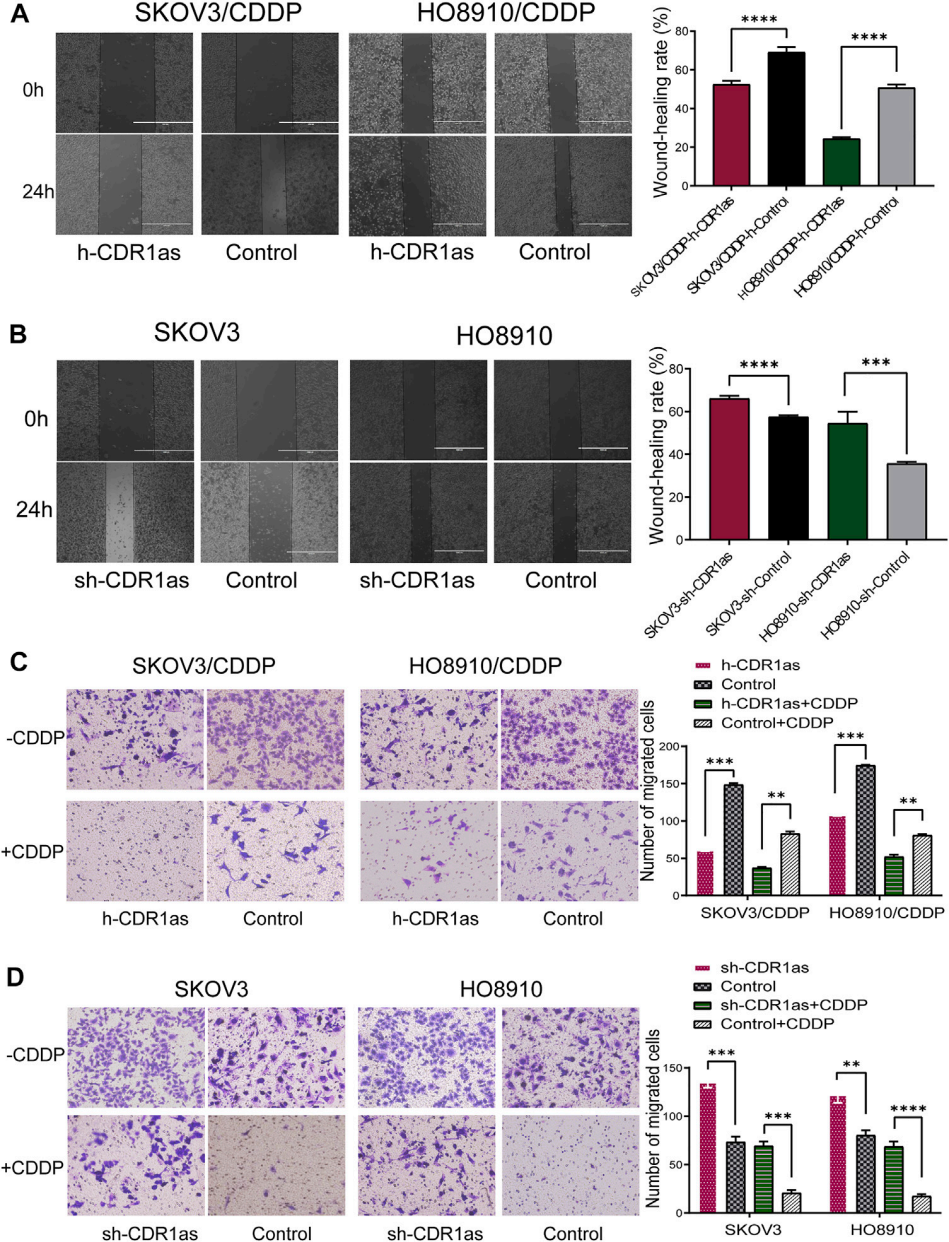
CDR1as reduces the cell migration and invasion. **(A,B)** Wound healing assays detected the effect of CDR1as on cell migration. **(C,D)** Cell invasion analysis of CDDP-resistant and -sensitive cell lines on CDR1as expression. These results were presented as the mean ± SDs. **p < 0.01, ***p < 0.001 and ****p < 0.0001.

MTT assays showed that SKOV3/CDDP and HO8910/CDDP had acquired CDDP resistance. The half maximal inhibitory concentration (IC50) of CDDP was higher in SKOV3/CDDP and HO8910/CDDP cells than in the parental cells SKOV3 and HO8910 cells (Figures 2C,D). CCK-8 assays showed that the overexpression of CDR1as reduced the ability of cell proliferation in SKOV3/CDDP and HO8910/CDDP cells at the IC50 of CDDP, while downregulated CDR1as increased the proliferation of SKOV3 and HO8910 cells (Figures 2E,F). Simultaneously, EdU assays also demonstrated that CDR1as inhibited proliferative capacities of CDDP-sensitive and -resistant OC cells (Figures 2G,H). Furthermore, cell apoptosis was determined by Annexin V-APC and PI double staining and flow cytometry. As shown in Figure 3A, in SKOV3/CDDP and HO8910/CDDP cells, the number of apoptotic cells (B + D) was increased when CDR1as was overexpressed and the cells were treated CDDP compared with the control. In addition, the knockdown of CDR1as decreased the extent of cell apoptosis in SKOV3 and HO8910 at a concentration of 5 μg/ml CDDP (Figure 3B). These results demonstrated that CDR1as could facilitate cell apoptosis and suppress CDDP chemoresistance in OC. In addition, wound healing and Transwell assays were used to confirm the effect of CDR1as on cell migration and invasion in OC. Results from wound healing assays showed that the overexpression of CDR1as significantly attenuated migration in both SKOV3/CDDP and HO8910/CDDP cells, while silencing CDR1as reserved this phenomenon in SKOV3 and HO8910 cells (Figures 4A,B). Transwell assays indicated that the overexpression of CDR1as could restrain the invasion ability of SKOV3/CDDP and HO8910/CDDP cells in the presence of CDDP while cell invasion was promoted with the reduced expression of CDR1as in SKOV3 and HO8910 cells (Figures 4C,D **)**.

### CDR1as Suppressed the CDDP Chemoresistance in OC Cells *In Vivo*

**FIGURE 5:**
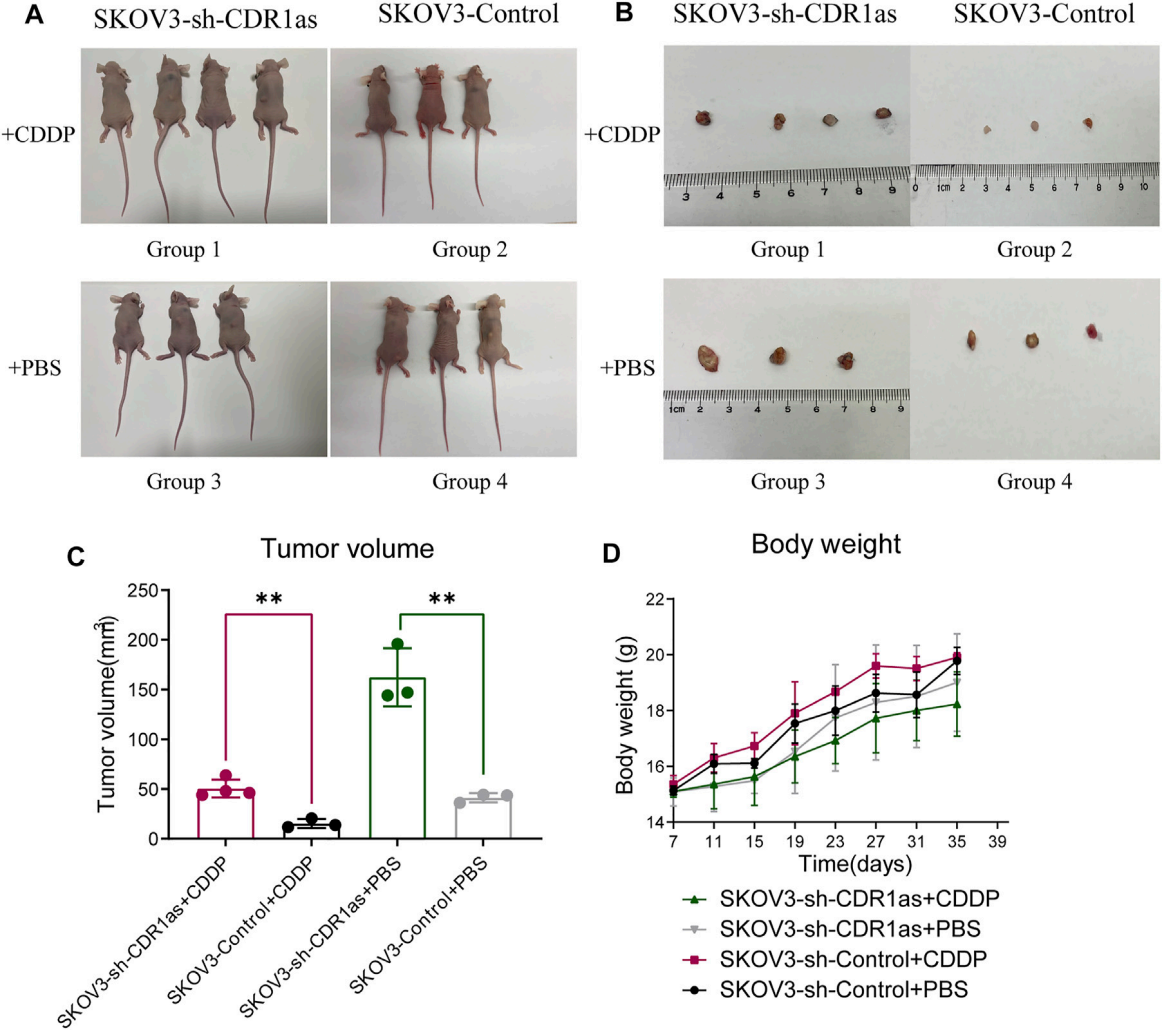
CDR1as promotes CDDP sensitive of OC cells *in vivo*. **(A)** The tumor burden of SKOV3 cells transfected with sh-CDR1as or Control lentivirus. **(B)** The subcutaneous xenograft tumors with or without CDDP treatment were isolated. **(C)** Tumor volume was measured using a caliper. **(D)** The growth curves of nude mice body weight. These results were presented as the mean ± SDs. **p <0.01.

Next, we investigated the clinical relevance of the association between CDR1as and CDDP resistance *in vivo*. We subcutaneously injected 1 × 10^7^ SKOV3 cells that were stably transfected with or without knockdown CDR1as into the each dorsal flanks of female BALB/c nude mice. The mice were then divided into four groups: group 1, SKOV3-sh-CDR1as + CDDP; group 2, SKOV3-sh-CDR1as + PBS; group 3, SKOV3-Control + CDDP; and group 4, SKOV3-Control + PBS. CDDP (5 mg/kg) were injected into group 1 and group 3 twice a week, while group 2 and group 4 were injected into the same dose of PBS (Figures 5A,B). The tumor volume and body weight measurements of nude mice were taken once 4 days. The inhibition of CDR1as in OC cells significantly increased tumor growth (Figures 5C,D). Therefore, CDR1as promoted the response of OC cells to CDDP treatment and suppressed CDDP chemoresistance *in vivo.*

### CDR1as Exerts Functionality by Sponging MiR-1299

**FIGURE 6:**
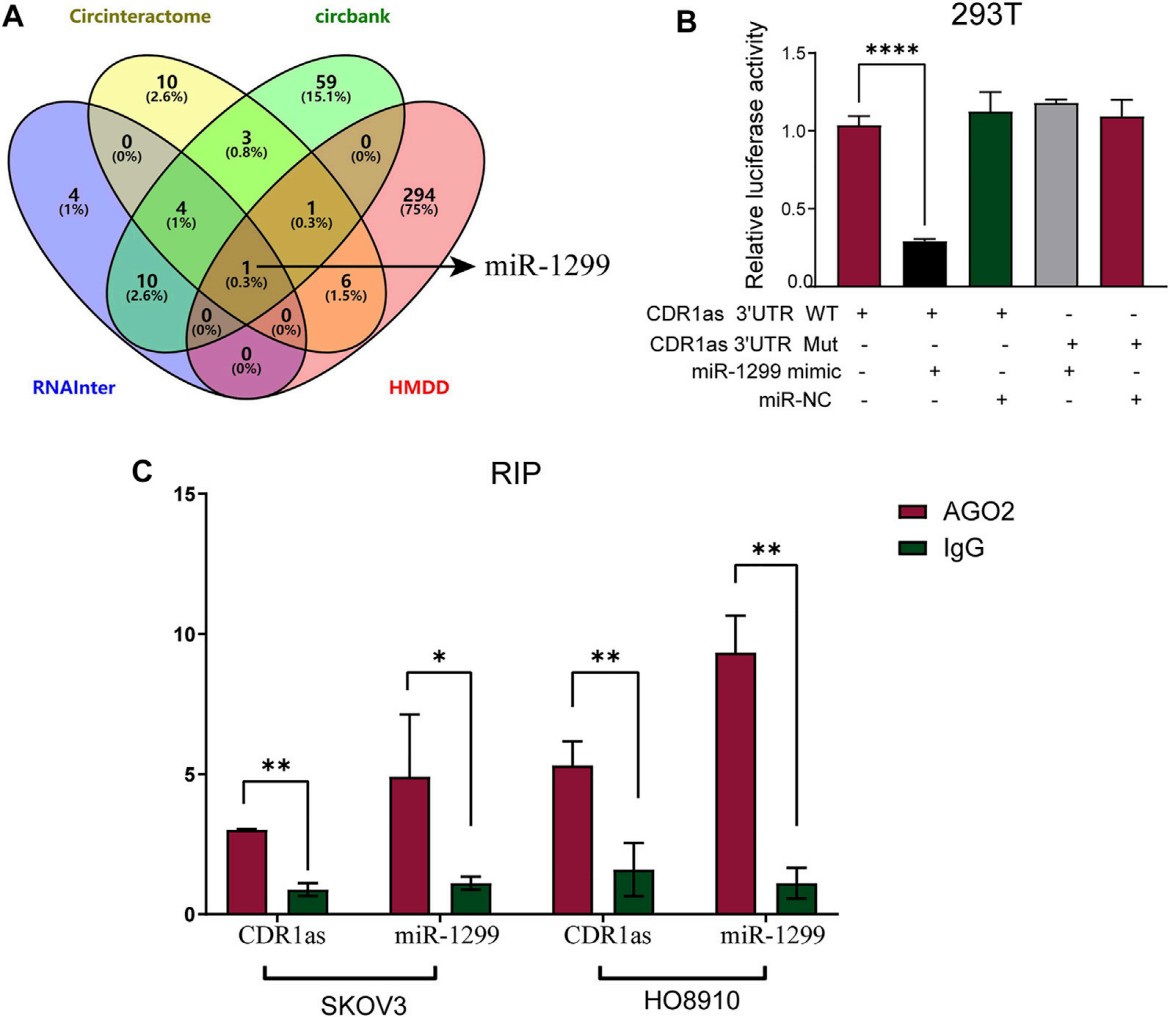
CDR1as serves as a miRNA sponge of miR-1299. **(A)** RNAInter, CircInteraction, CircBank, and HMDD databases were used to predict the target miRNA of CDR1as. **(B)** The interaction between CDR1as and miR-1299 in 293T cells was verified using dual luciferase report assay. **(C)** RIP experiments enriched CDR1as and miR-1299 by qRT-PCR in OC cells. These results were presented as the mean ± SDs. **p < 0.01 and ****p < 0.0001.

To address whether CDR1as could act as a sponge for miRNAs in OC cells, we first performed the bioinformatics analysis for target prediction. Three online tools RNAInter (RNAInter), CircInteraction (CircInteraction), and CircBank (CircBank) were used to predict miRNAs that contain binding sites to the CDR1as sequence. Then, another online database (HMDD) was used to detect miRNAs associated with OC; this database features experimental evidence. Subsequent Venn analysis, as shown in Figure 6A, indicated that miR-1299 (which contained 19 binding sites) was filtered out and predicted to be a possible target for CDR1as. miR-1299 was associated with 19 7mer sponge sites in the 3′-UTR of CDR1as. To confirm the interaction between CDR1as and miR-1299, a dual luciferase reporter assay was designed in 293T cells. The CDR1as full-length wild type (CDR1as-wt) and mutant type (CDR1as-mut; without the miR-1299 binding site) were cloned into a luciferase reporter vector. miR-1299 mimics significantly reduced the luciferase activity of CDR1as-wt. However, there was no obvious effect of the CDR1as-mut reporter following transfection with miR-1299 mimics (Figure 6B). Furthermore, the RNA Immunoprecipitation (RIP) assays were performed with argonaute 2 (AGO2) antibody in SKOV3 and HO8910 cells. We then used qRT-PCR to detect the expression levels of CDR1as and miR-1299 enriched by anti-AGO2 antibody and anti-IgG antibody. Our results demonstrated the specific enrichment of CDR1as and miR-1299 in the AGO2 group when compared to the IgG group (Figure 6C). These results indicated that CDR1as might function as a miR-1299 sponge in OC cells.

### MiR-1299 Promoted CDDP Chemoresistance in OC

**FIGURE 7:**
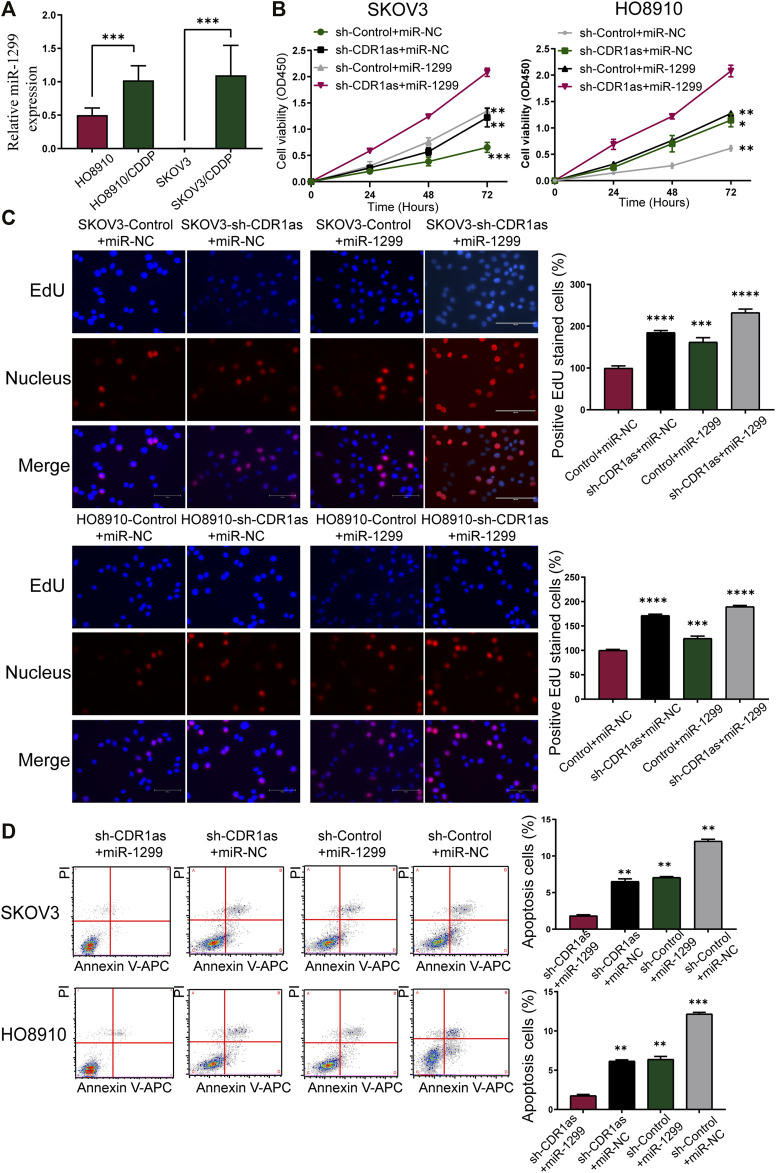
The relationship between miR-1299 and CDR1as in OC cells. **(A)** The expression of miR-1299 in cell lines by qRT-PCR. **(B,C)** Proliferation of CDR1as-knockout OC cells that transfected with miR-1299 mimic or miR-NC was detected by CCK-8 **(B)** and EdU assays **(C)**. **(D)** Flow cytometry analyzed the CDR1as-knockout OC cell apoptosis rate with or without miR-1299 transfected. These results were presented as the mean ± SDs. *p < 0.05, **p <0.01, ***p < 0.001, and ****p < 0.0001.

To evaluate the potential functional association between miR-1299 and OC cells, we confirmed the expression of miR-1299 in CDPP-sensitive and -resistant OC cells. By qRT-PCR, we found that the expression of miR-1299 was increased in SKOV3/CDDP and HO8910/CDDP compared with SKOV3 and HO8910 (Figure 7A). We also found that the effects of miR-1299 were opposite to those of CDR1as on CDDP-resistant OC cells. To further verify the role of miR-1299 in OC, we designed rescue experiments. SKOV3 and HO8910 cells were transfected as follows: 1) sh-Control + miR-NC; 2) sh-CDR1as + miR-NC; 3) sh-Control + miR-1299 mimic; and 4) sh-CDR1as + miR-1299 mimic. CCK-8 (Figure 7B) and EdU assays (Figure 7C) showed that the miR-1299 mimic significantly reversed the cell proliferation effects caused by the downregulation of CDR1as. Flow cytometry analyses further indicated that co-transfection with sh-CDR1as and miR-1299 mimic significantly suppressed the cell apoptosis when compared with the controls (sh-Control + miR-NC). Moreover, miR-1299 could also inhibit the cell apoptosis in OC cells (Figure 7D). Based on these results, we found that the increased cell proliferation and decreased cell apoptosis caused by the knockdown of CDR1as could be reversed by the overexpression of miR-1299 in OC cells.

### PPP1R12B Was Identified as a Direct Target of MiR-1299

**FIGURE 8:**
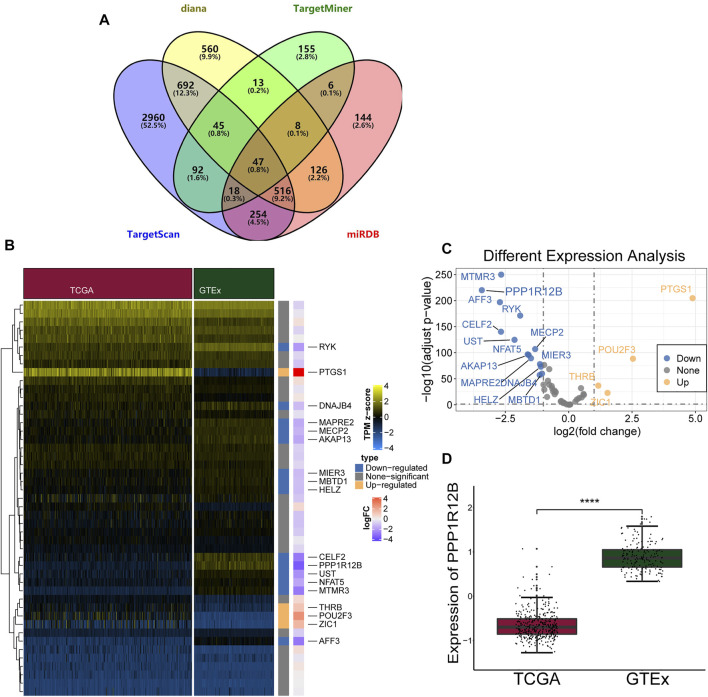
The predicted target mRNAs of miR-1299. **(A)** Candidate mRNAs predicted by four databases to target miR-1299. **(B,C)** Heatmap **(B)** and volcano plots **(C)** showed different expressed genes that bind to miR-1299 in TCGA and GTEx. **(D)** The different expression of PPP1R12B in TCGA and GTEx. These results were presented as the mean ± SDs. ****p < 0.0001.

**FIGURE 9:**
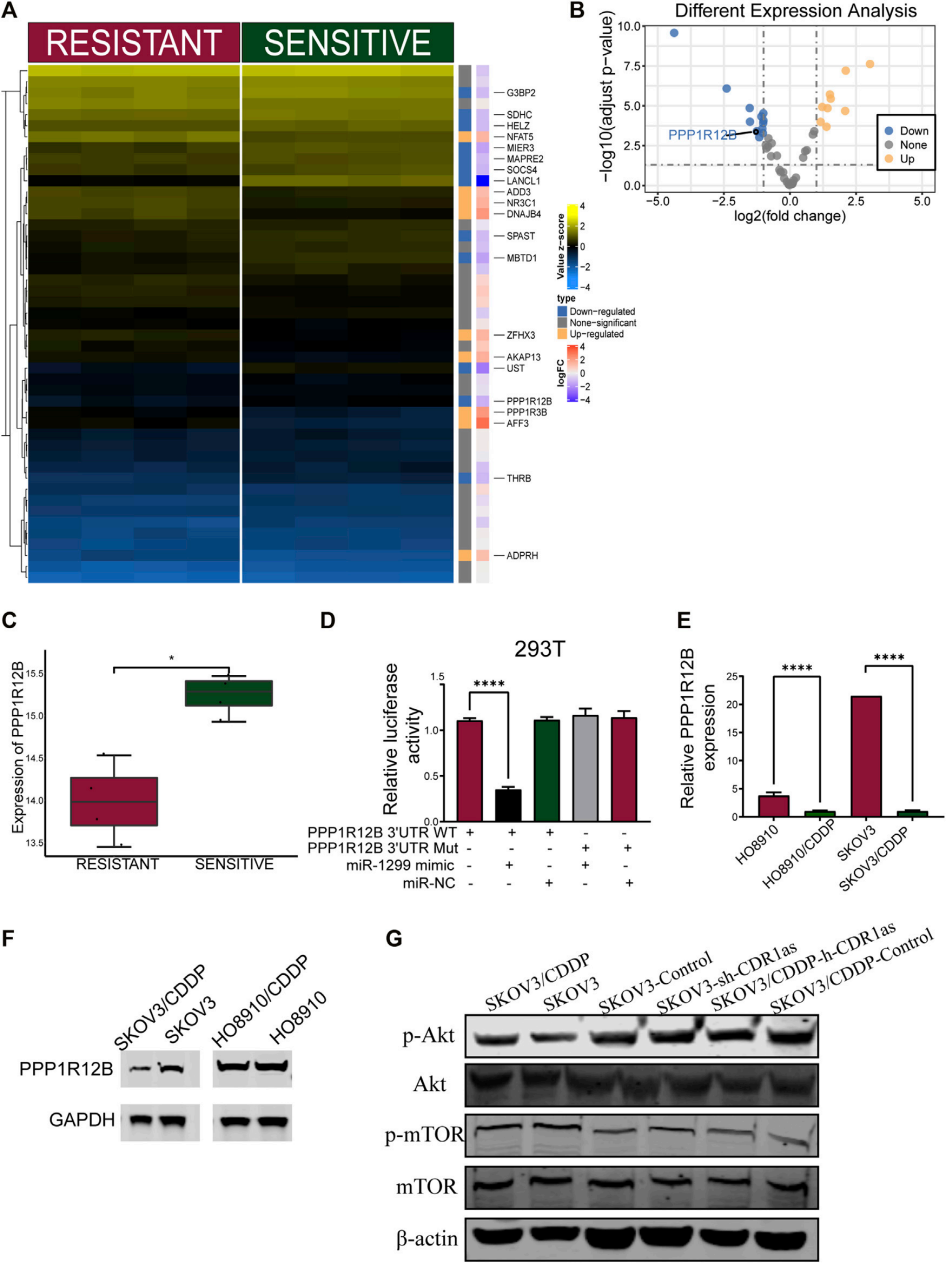
PPP1R12B is a direct target of miR-1299. **(A,B)** The heatmap **(A)** and volcano plot **(B)** revealed the mRNAs expression profiles in GSE45553. **(C)** The expression of PPP1R12B in CDDP-resistant and -sensitive cell lines by GSE45553. **(D)** The interaction between PPP1R12B and miR-1299 in 293T cells was verified using dual luciferase report assay. **(E)** PPP1R12B expression in cells lines. **(F)** The protein expression of PPP1R12B in cell lines by Western blotting. **(G)** Knockout CDR1as activates Akt/mTOR signaling pathway. These results were presented as the mean ± SDs. *p < 0.05 and ****p < 0.0001.

The downstream target genes of miR-1299 were further analyzed with four databases (Diana, TargetMiner, TargetScan, and miRDB) so that we could better identify the mode of molecular regulation. We identified 47 mRNAs (Figure 8A). Figure 8B shows the expression profiles of mRNAs obtained from the TCGA and GTEx databases. PPP1R12B showed the most significant downregulatory trend in the TCGA cohort compared with the normal tissue cohort (Figures 8C,D). Then, we further identified the target genes using the GSE45553 dataset and found that PPP1R12B was significantly downregulated in CDDP resistant cell lines (Figures 9A,B). Therefore, we hypothesized that PPP1R12B might be a direct target of miR-1299. Dual-luciferase reporter assays were performed to confirm the binding of PPP1R12B to miR-1299 in 293T cells. The luciferase reporter vector that contained the wild-type miR-1299-binding sites at the PPP1R12B 3′-UTR and miR-1299 were decreased relative to those containing mutated binding sites (Figure 9C). In addition, we investigated the expression of PPP1R12B in OC cells. As shown in Figure 9D, compared with CDDP-resistant OC cells, CDDP-sensitive OC cells showed an obvious increase in the expression of PPP1R12B mRNA by qRT-PCR. Western blotting also verified the protein expression of PPP1R12B in OC cells (Figure 9E). Results indicated that PPP1R12B was downregulated in CDDP-resistant cells and was directly targeted by miR-1299.

### CDR1as Inhibited the Akt/mTOR Signaling Pathway in OC

Western blotting analysis was used to confirm the involvement of the Akt/mTOR signaling pathway in CDDP-resistant OC. The expression levels of p-Akt and p-mTOR were increased in SKOV3/CDDP cells (Figure 9F). Furthermore, the roles of CDR1as in the Akt/mTOR pathway were also investigated. We discovered a significant trend in the expression of p-Akt and p-mTOR that were contrary to the level of CDR1as expression. The levels of CDR1as did not alter the expression of Akt and mTOR. These results suggested that the Akt/mTOR signaling pathway was activated by the knockdown of CDR1as.

## Discussion

CDDP is one of the most widely used drugs in the chemotherapy treatment of OC. Despite the improvements in chemoradiotherapy, targeted therapy, and immunotherapy, CDDP chemoresistance is one of the most significant factors that lead to treatment failure in OC patients and seriously threaten the survival rate of OC patients (Zhang et al., 2020). Consequently, understanding the pathogenesis of CDDP resistance in OC is vital if we are to develop better therapy and prognosis of patients. Recently, an increasing number of studies have verified the critical roles of circRNAs in tumor development and CDDP chemoresistance (Zhang et al., 2019). An increasing body of literature now supports the fact that CDR1as is deregulated in many types of cancers, such as bladder cancer, liver cancer, nasopharyngeal cancer, and esophageal squamous cell cancer (Zhong et al., 2019, Meng et al., 2020). Therefore, CDR1as plays a vital role in the progression and metastasis of CDDP chemoresistance in OC. However, the molecular mechanisms and underlying role of CDR1as remain obscure and need to be investigated in CDDP-resistant OC. In our research, multiple bioinformatics methods and datasets were employed and confirmed that CDR1as expression was downregulated in OC tissues and cells. Furthermore, compared with CDDP-sensitive OC cells, CDR1as expression was significantly reduced in CDDP-resistant OC cells. The downregulated expression of CDR1as suppressed OC tumorigenesis and predicted CDDP resistance and a poor prognosis in OC patients. We are the first to determine the role of circRNA CDR1as in CDDP resistance in OC. Next, we investigated the functions of CDR1as as a key regulator in cell proliferation, apoptosis, migration, and invasion *in vitro* and in nude mouse tumor xenografts *in vivo*.

To investigate the effect of CDR1as on CDDP chemoresistance in OC, we performed CCK-8 and EdU assays. We found that the upregulation of CDR1as inhibited the proliferation of CDDP resistance OC cells in response to CDDP treatment. Then, wound healing and Transwell assays were performed; we found that the overexpression of CDR1as promoted cell migration and invasion in CDDP-resistant OC cells. Furthermore, flow cytometry analysis showed that the upregulation of CDR1as could promote cell apoptosis in CDDP-resistant OC cells. Moreover, a nude mouse tumor xenograft model was established to further investigate the clinical relevance of CDR1as on CDDP chemoresistance OC. The tumor xenograft data indicated that the knockdown of CDR1as increased tumor growth and enhanced the cell resistance to CDDP treatment. Based on these experiments, CDR1as acts as a tumor suppressor in OC and could suppress CDDP chemoresistance.

It is well known that circRNAs can act as sponges of miRNAs to modulate gene expression in cancer. For example, circFAM13B can sponge miR-212 to promote the proliferation of hepatocellular carcinoma (Xie et al., 2021). CircCUL2 regulates gastric cancer malignant transformation by sponging miR-142-3p (Peng et al., 2020). In oral squamous carcinoma cells, circ-SCMH suppresses CDDP chemoresistance by sponging miR-3383p and regulating LIN28B (Qiu et al., 2021). In this study, we used three online databases for bioinformatics analysis to predict the potential downstream miRNAs of CDR1as. Interestingly, we observed that CDR1as was a sponge of miR-1299. By dual-luciferase reporter assays and RIP assays, we confirmed the direct interaction of miR-1299 and CDR1as. Furthermore, the abundance of miR-1299 was increased in CDPP-resistant OC cells compared with their parent cells. We also carried out rescue experiments. miR-1299 was shown to contribute to CDDP resistance by CCK-8, EdU assays, and flow cytometric analysis.

In light of the ceRNA hypothesis, circRNAs can form a new complex regulatory network to regulate miRNA target gene expression (Thomson and dinger, 2016). By analyzing four databases, we found that PPP1R12B was the target gene of miR-1299 and was significantly downregulated in CDDP-resistant OC. Dual-luciferase reporter assays demonstrated that miR-1299 could target the 3′UTR of PPP1R12B. qRT-PCR was also used to demonstrate the expression of PPP1R12B in OC cells. Western blotting also confirmed the protein expression of PPP1R12B. This was the first evidence to indicate that PPP1R12B participates in CDDP chemoresistance in OC. However, the underlying upstream mechanism of PPP1R12B in CDDP chemoresistance in OC has yet to be fully investigated.

The Akt/mammalian target of rapamycin (mTOR) signaling pathway is a classic intracellular pathway and plays an important role in various tumors (Deng et al., 2019). Irregularities in the Akt/mTOR signaling pathway are reported to be a significant therapeutic target in OC (Brabec and Kasparkova, 2005). In osteoarthritis, the downregulation of CDR1as can activate the AKT/mTOR signaling pathway (Zhou et al., 2020). Currently, the relationship between CDR1as and the Akt/mTOR signaling pathway has not been explored in CDDP chemoresistance OC. In this study, we confirmed that the Akt/mTOR pathway was activated in CDDP-resistant cells in OC. The data also showed that the downregulation of CDR1as could enhance the expression of p-Akt and p-mTOR. However, there were no significant changes in the expression of total Akt or total mTOR. However, the mechanisms underlying the action of CDR1as on the Akt/mTOR signaling pathway in CDDP chemoresistance OC has yet to be investigated.

Based on these findings, we carried out a series of studies to demonstrate the role of the CDR1as/miR-1299/PPP1R12B axis in OC. We found that the levels of CDR1as were decreased in OC tissues and cells. Compared with CDDP-sensitive OC cells, the expression of CDR1as was downregulated in CDDP-resistant OC cells. CDR1as could bind with miR-1299 to target PPP1R12B mRNA. CDR1as enhanced CDDP chemotherapy sensitivity in OC. The downregulation of CDR1as activated the Akt/mTOR signaling pathway. Together, our findings reveal the relationship between CDR1as, miR-1299, and PPP1R12B mRNA in CDDP chemoresistance in OC. Our study also provides novel evidence and sheds light on highlighting a therapeutic target for CDDP chemoresistance in OC patients.

## Data Availability

The datasets presented in this study can be found in online repositories. The names of the repository/repositories and accession number(s) can be found in the article/Supplementary Material.
