# Supplementary figures and images for: Circular RNA CDR1as Alleviates Cisplatin-Based Chemoresistance by Suppressing MiR-1299 in Ovarian Cancer
Source: Front Genet. 2022 Jan 26;12:815448. doi: 10.3389/fgene.2021.815448 (PMC8826532; doi:10.3389/fgene.2021.815448)

9G-p-AKT

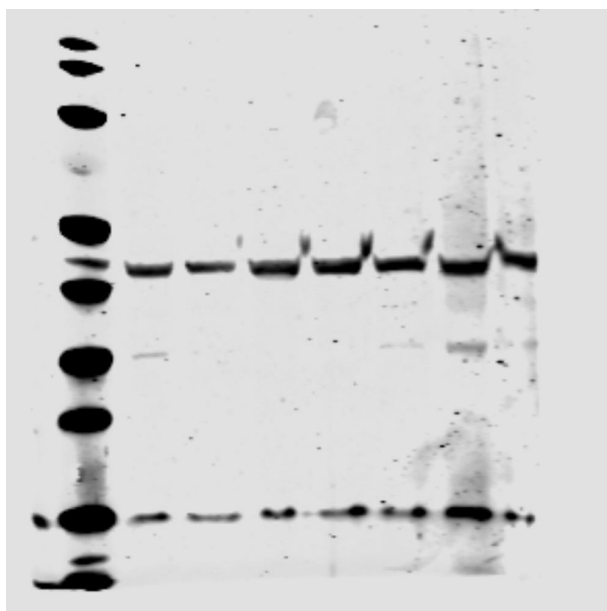

9G-mtor

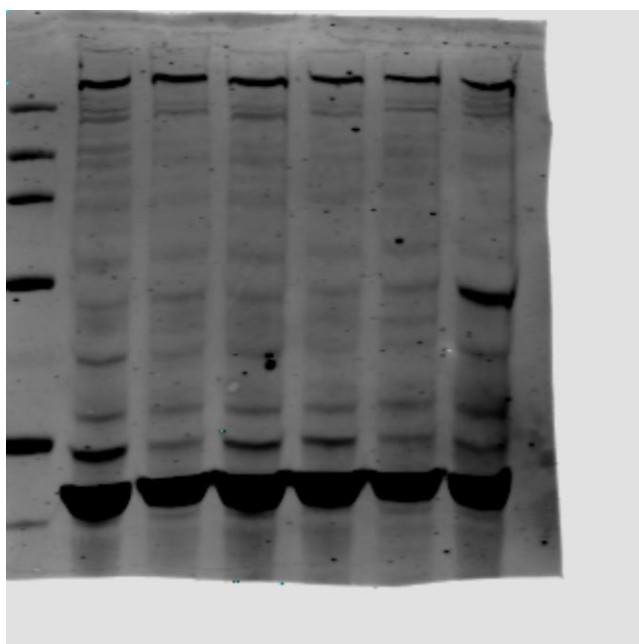

9G-Akt

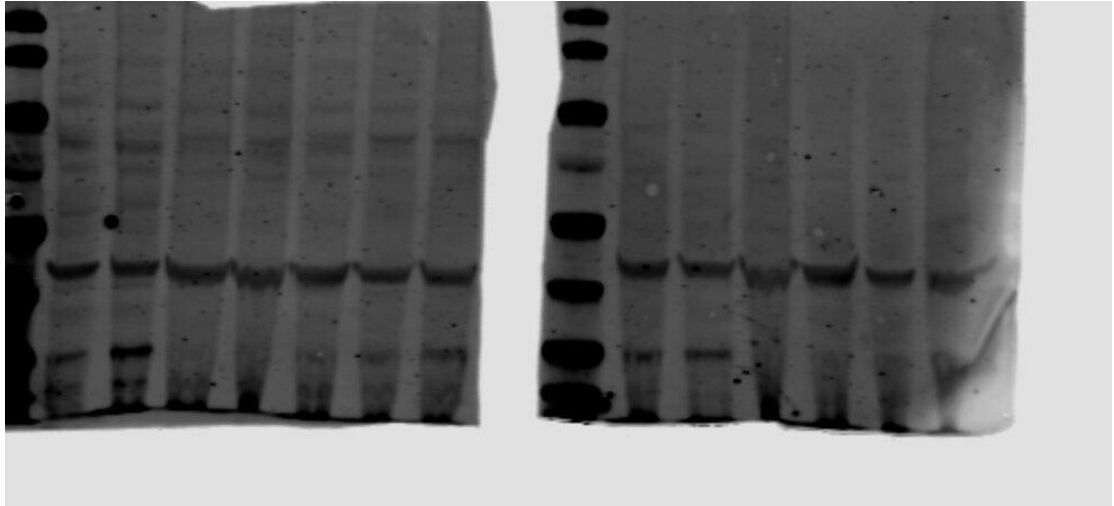

9E

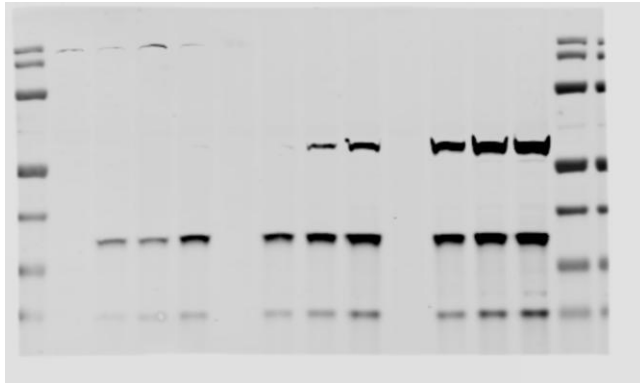

9G-mtor and actin

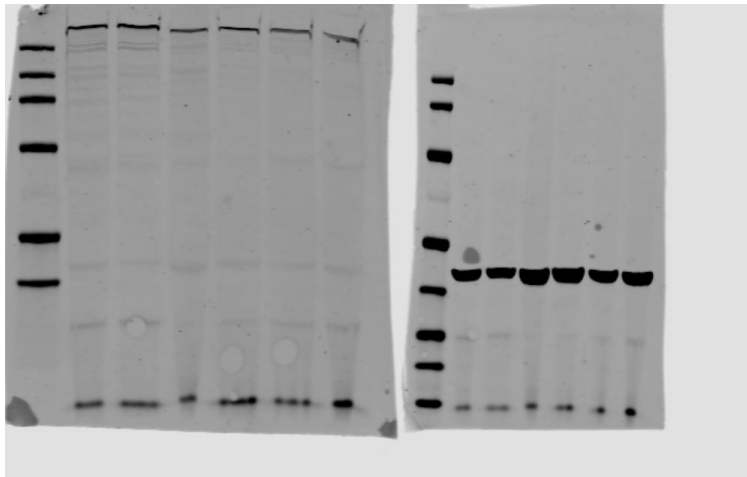

Supplement: Supplementary file 1 [file DataSheet7.PDF]
