# Supplementary material for: Circular RNA CDR1as Alleviates Cisplatin-Based Chemoresistance by Suppressing MiR-1299 in Ovarian Cancer
Source: Front Genet. 2022 Jan 26;12:815448. doi: 10.3389/fgene.2021.815448 (PMC8826532; doi:10.3389/fgene.2021.815448)
Supplement: Supplementary file 2 [file DataSheet2.PDF]

Apogee Flow Cytometry Report
Apogee Flow Cytometer

Acquisition Date:	19 May 2021 15:59:32
Filename:	Sample_210519_1399_0.fcs
Sample ID:	Sample_210519_1399
Operator:	A0149\ApogeeFlow
Protocol:	

SALS-LALS [Peak-Peak]

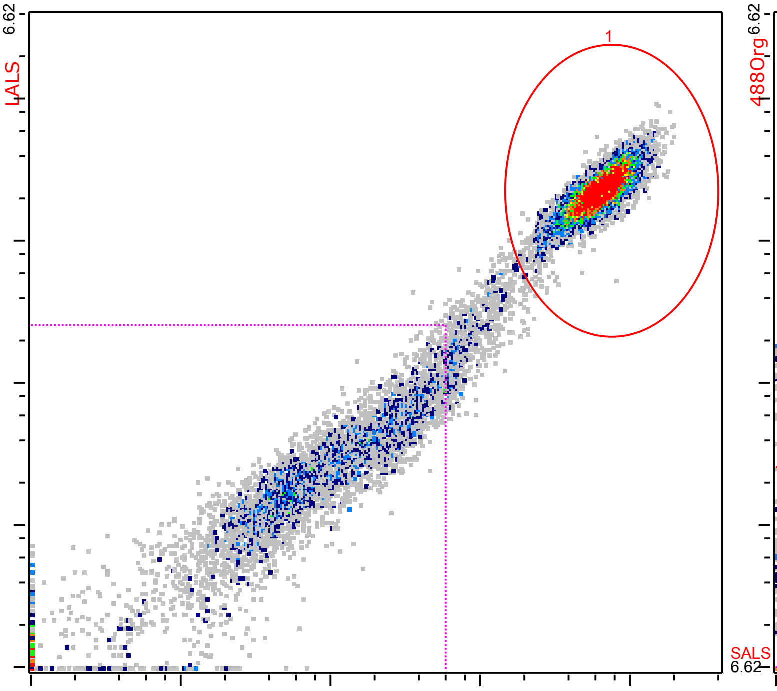

488Grn-488Org [Peak-Peak]

Gated by 1

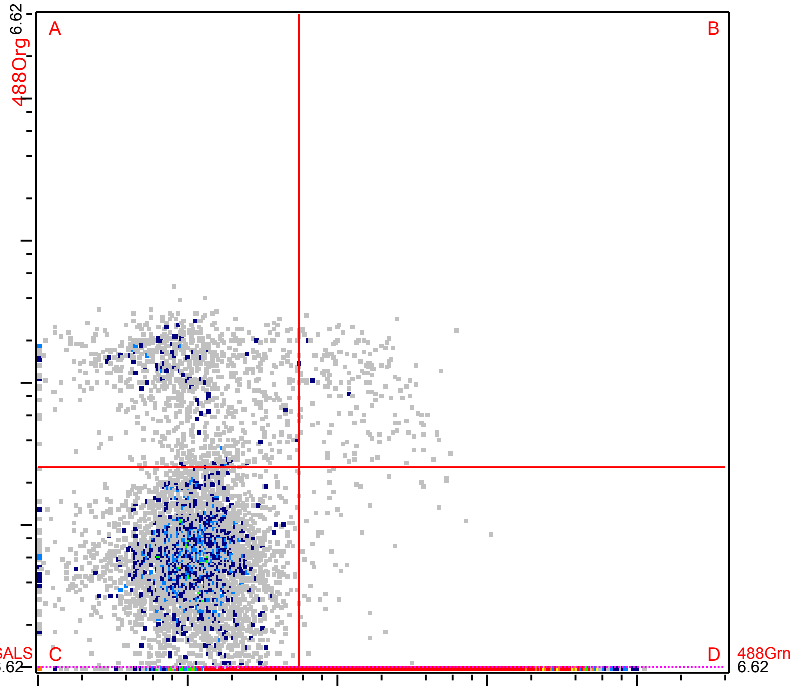

488Grn-638Red [Peak-Peak]

Gated by 1

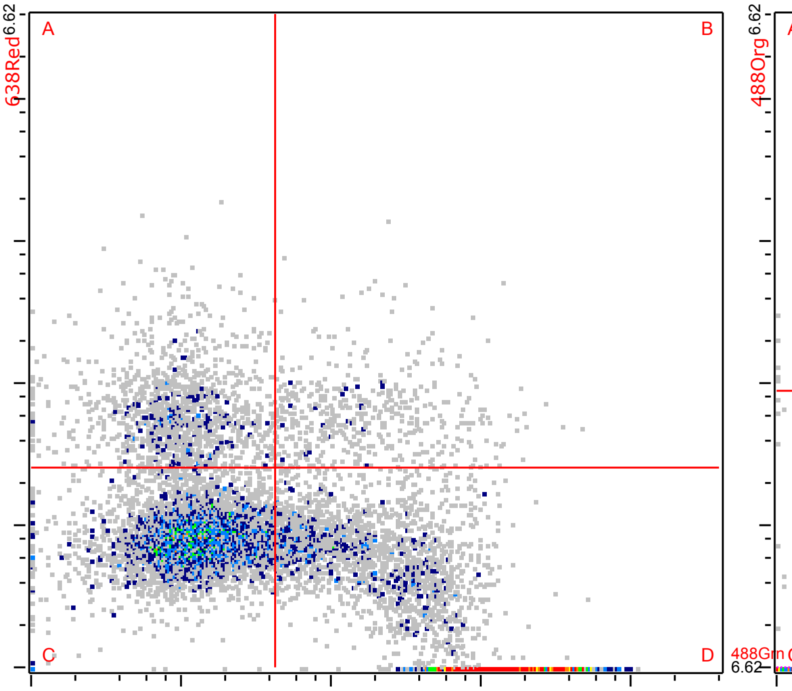

638Red-488Org [Peak-Peak]

Gated by 1

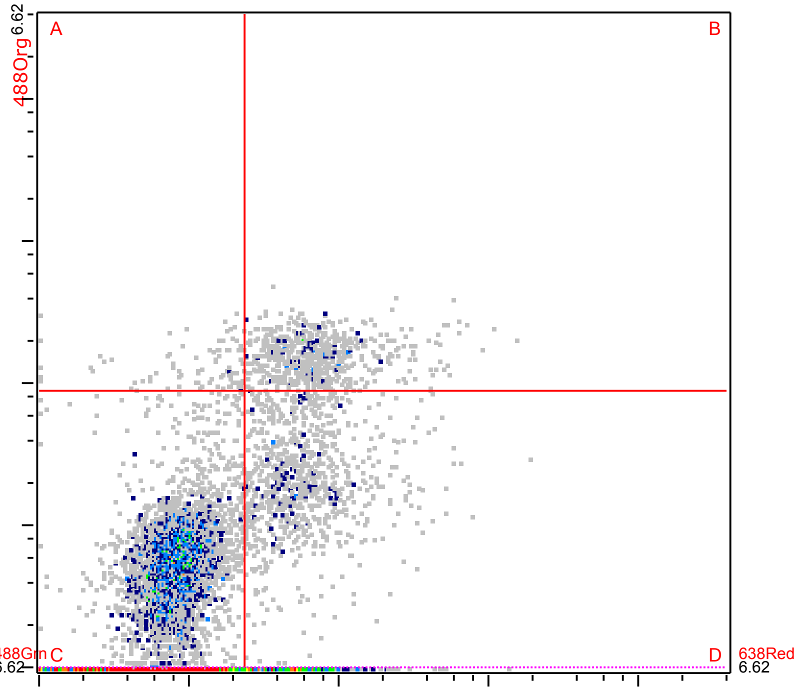

Apogee Flow Cytometry Report
Apogee Flow Cytometer

Acquisition Date:	19 May 2021 15:59:32
Filename:	Sample_210519_1399_0.fcs
Sample ID:	Sample_210519_1399
Operator:	A0149\ApogeeFlow
Protocol:	

Cytogram ROI Statistics

ROI ID	Events	Events/µl	%	Ratio	Mean X	Mean Y
1	10082	1493.6	62.7%		673144	237539
488Grn--A	1031	152.7	10.2%			
488Grn--B	171	25.3	1.7%			
488Grn--C	5447	807.0	54.0%			
488Grn--D	3433	508.6	34.1%			
488Grn--A	1323	196.0	13.1%			
488Grn--B	495	73.3	4.9%			
488Grn--C	4812	712.9	47.7%			
488Grn--D	3452	511.4	34.2%			
638Red--A	89	13.2	0.9%			
638Red--B	671	99.4	6.7%			
638Red--C	8113	1201.9	80.5%			
638Red--D	1209	179.1	12.0%			

Acquisition Parameters

Channel	PMT	Gain	Thresh (OR)	Subtraction
SALS	330	1.00	954	
LALS	350	1.00	414	
488Grn	285	1.00		0.00%, 0.00%, 0.00%
488Org	340	1.00	1	28.00%, 0.00%, 0.00%
488Red	520	1.00		0.00%, 0.00%, 0.00%
488DpRed	500	1.00		0.00%, 0.00%, 0.00%

Instrument Settings

Pressure	Dilution	Sample Flow	Acquisition Time
75 counts	factor of 1	15.00 ul/min	27 secs

CYT: Universal
CYTSN: 0149
Apogee Flow Systems Limited

APOGEE FLOW SYSTEMS

Apogee Flow Cytometry Report
Apogee Flow Cytometer

Acquisition Date:	19 May 2021 16:08:24
Filename:	Sample_210519_1403_0.fcs
Sample ID:	Sample_210519_1403
Operator:	A0149\ApogeeFlow
Protocol:	

SALS-LALS [Peak-Peak]

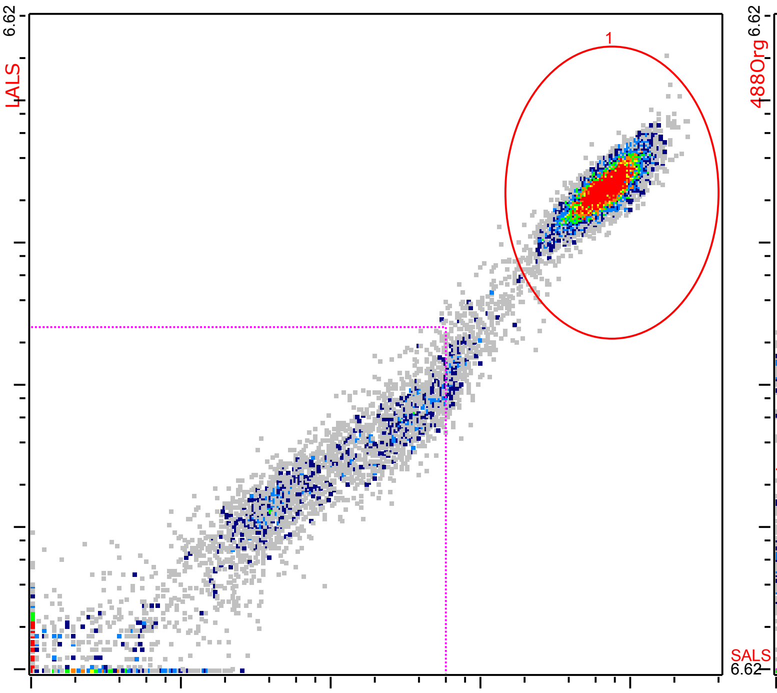

488Grn-488Org [Peak-Peak]

Gated by 1

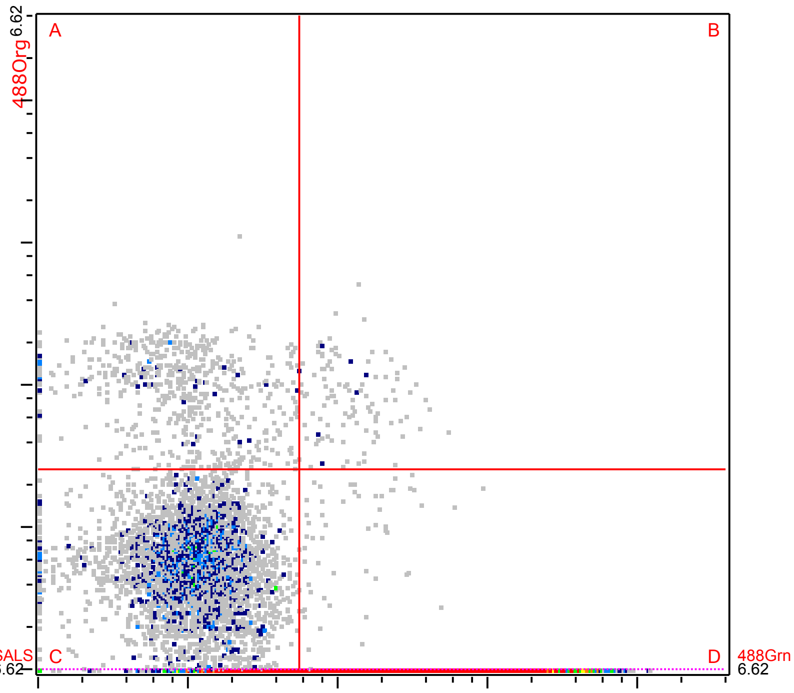

488Grn-638Red [Peak-Peak]

Gated by 1

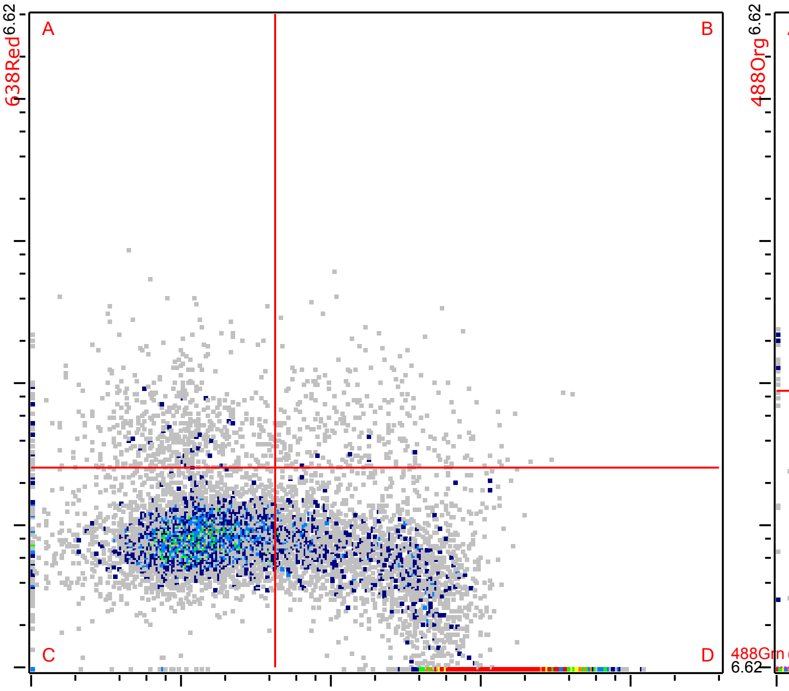

638Red-488Org [Peak-Peak]

Gated by 1

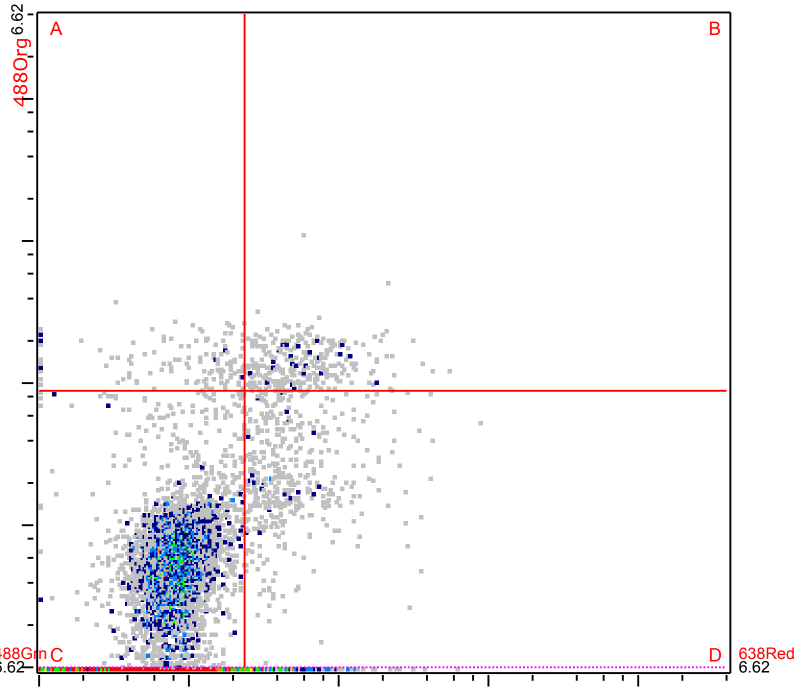

Apogee Flow Cytometry Report
Apogee Flow Cytometer

Acquisition Date: 19 May 2021 16:08:24
Filename: Sample_210519_1403_0.fcs
Sample ID: Sample_210519_1403
Operator: A0149\ApogeeFlow
Protocol:

Cytogram ROI Statistics

ROI ID	Events	Events/µl	%	Ratio	Mean X	Mean Y
1	10016	1430.9	66.9%		733603	257262
488Grn--A	633	90.4	6.3%			
488Grn--B	109	15.6	1.1%			
488Grn--C	5391	770.1	53.8%			
488Grn--D	3883	554.7	38.8%			
488Grn--A	585	83.6	5.8%			
488Grn--B	291	41.6	2.9%			
488Grn--C	5112	730.3	51.0%			
488Grn--D	4028	575.4	40.2%			
638Red--A	130	18.6	1.3%			
638Red--B	308	44.0	3.1%			
638Red--C	8970	1281.4	89.6%			
638Red--D	608	86.9	6.1%			

Acquisition Parameters

Channel	PMT	Gain	Thresh (OR)	Subtraction
SALS	330	1.00	954	
LALS	350	1.00	414	
488Grn	285	1.00		0.00%, 0.00%, 0.00%
488Org	340	1.00	1	28.00%, 0.00%, 0.00%
488Red	520	1.00		0.00%, 0.00%, 0.00%
488DpRed	500	1.00		0.00%, 0.00%, 0.00%

Instrument Settings

Pressure	Dilution	Sample Flow	Acquisition Time
75 counts	factor of 1	15.00 ul/min	28 secs

CYT: Universal
CYTSN: 0149
Apogee Flow Systems Limited

Apogee Flow Cytometry Report
Apogee Flow Cytometer

Acquisition Date:	17 May 2021 15:31:56
Filename:	Sample_210517_1357_0.fcs
Sample ID:	Sample_210517_1357
Operator:	A0149\ApogeeFlow
Protocol:	

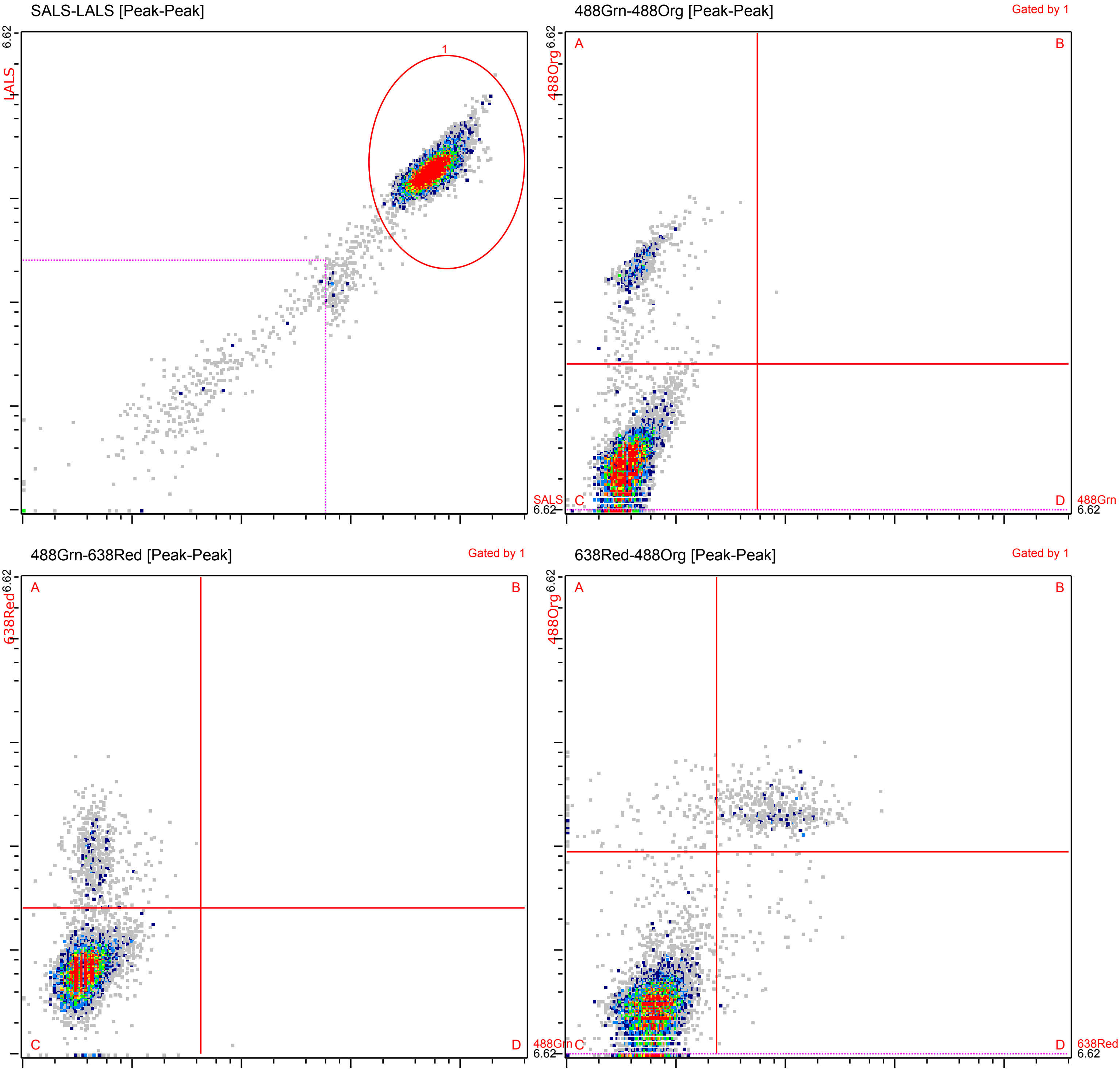

Apogee Flow Cytometry Report
Apogee Flow Cytometer

Acquisition Date:	17 May 2021 15:31:56
Filename:	Sample_210517_1357_0.fcs
Sample ID:	Sample_210517_1357
Operator:	A0149\ApogeeFlow
Protocol:	

Cytogram ROI Statistics

ROI ID	Events	Events/µl	%	Ratio	Mean X	Mean Y
1	10110	3370.0	94.8%		581152	196156
488Grn--A	710	236.7	7.0%			
488Grn--B	1	0.3	0.0%			
488Grn--C	9399	3133.0	93.0%			
488Grn--D	0	0.0	0.0%			
488Grn--A	583	194.3	5.8%			
488Grn--B	0	0.0	0.0%			
488Grn--C	9526	3175.3	94.2%			
488Grn--D	1	0.3	0.0%			
638Red--A	116	38.7	1.1%			
638Red--B	513	171.0	5.1%			
638Red--C	9399	3133.0	93.0%			
638Red--D	82	27.3	0.8%			

Acquisition Parameters

Channel	PMT	Gain	Thresh (OR)	Subtraction
SALS	330	1.00	954	
LALS	350	1.00	414	
488Grn	285	1.00		0.00%, 0.00%, 0.00%
488Org	340	1.00	1	28.00%, 0.00%, 0.00%
488Red	520	1.00		0.00%, 0.00%, 0.00%
488DpRed	500	1.00		0.00%, 0.00%, 0.00%

Instrument Settings

Pressure	Dilution	Sample Flow	Acquisition Time
75 counts	factor of 1	15.00 ul/min	12 secs

CYT: Universal
CYTSN: 0149
Apogee Flow Systems Limited

APOGEE FLOW SYSTEMS

Apogee Flow Cytometry Report
Apogee Flow Cytometer

Acquisition Date:	18 May 2021 15:00:19
Filename:	Sample_210518_1373_0.fcs
Sample ID:	Sample_210518_1373
Operator:	A0149\ApogeeFlow
Protocol:	

SALS-LALS [Peak-Peak]

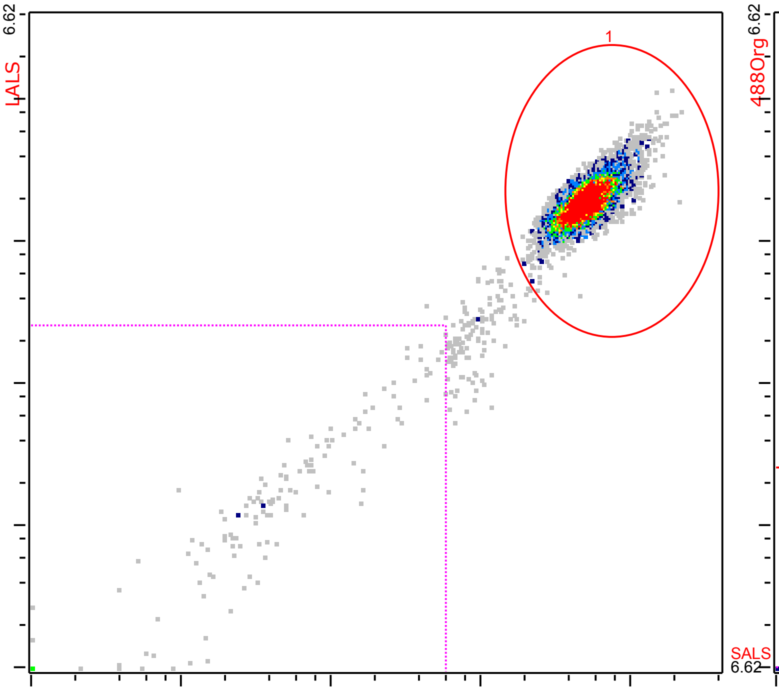

488Grn-488Org [Peak-Peak] Gated by 1

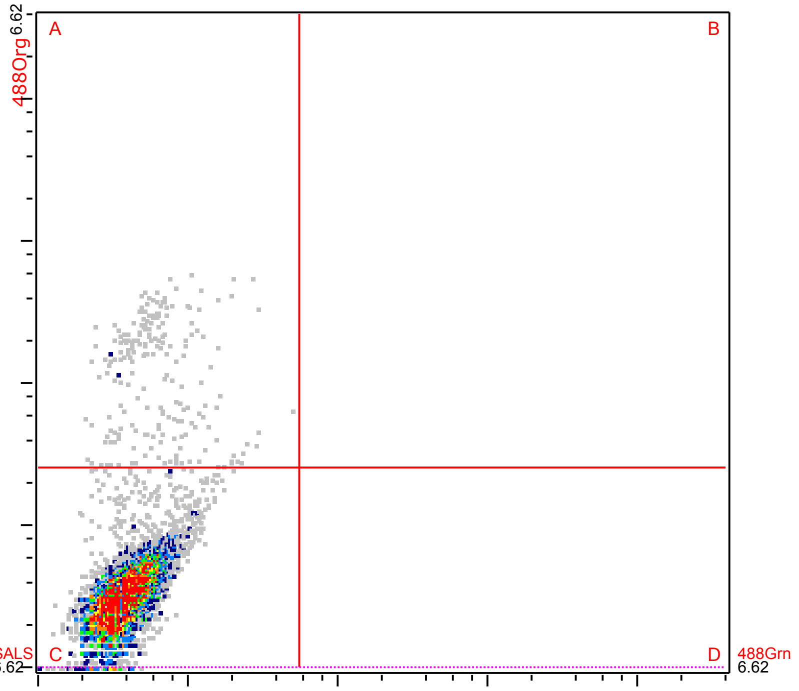

488Grn-638Red [Peak-Peak] Gated by 1

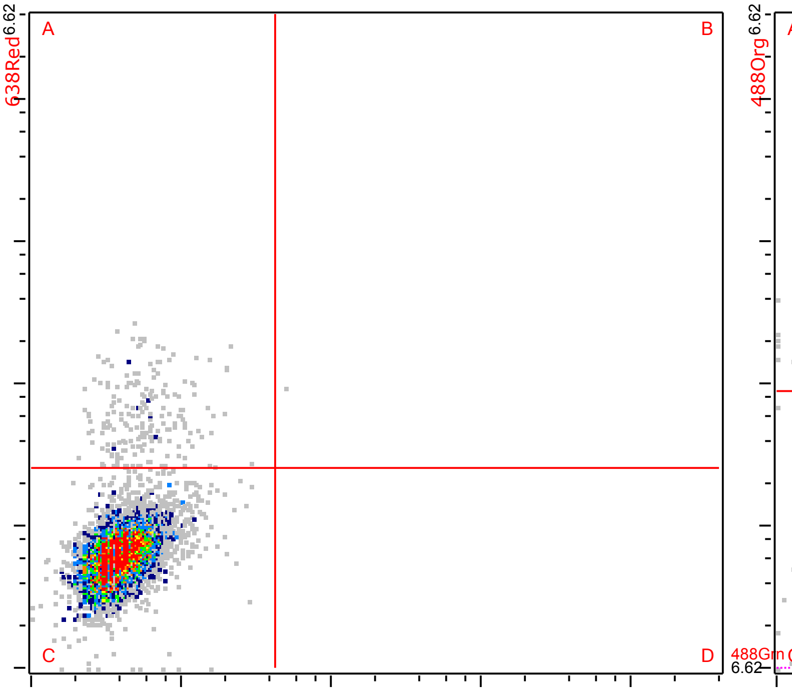

638Red-488Org [Peak-Peak] Gated by 1

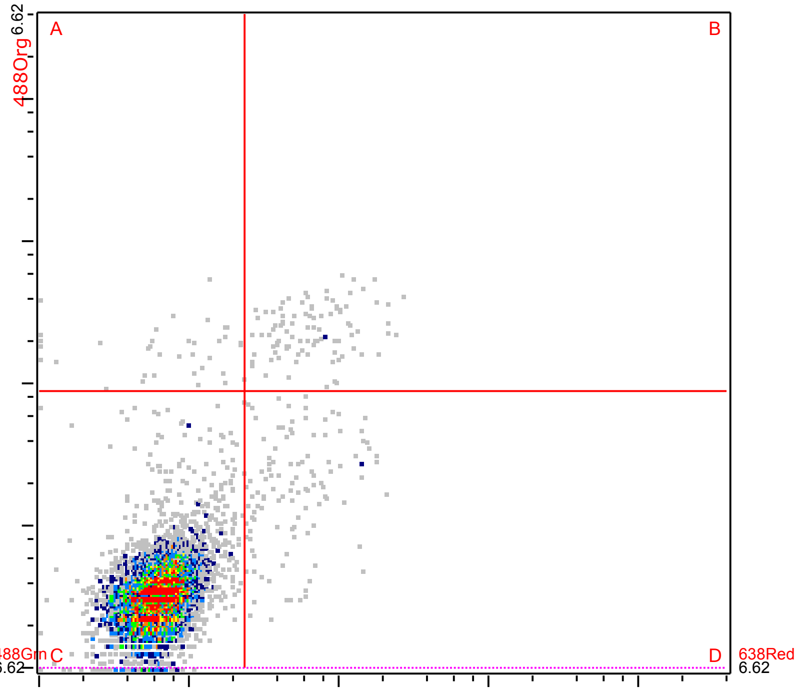

Apogee Flow Cytometry Report
Apogee Flow Cytometer

Acquisition Date:	18 May 2021 15:00:19
Filename:	Sample_210518_1373_0.fcs
Sample ID:	Sample_210518_1373
Operator:	A0149\ApogeeFlow
Protocol:	

Cytogram ROI Statistics

ROI ID	Events	Events/µl	%	Ratio	Mean X	Mean Y
1	10097	3365.7	97.6%		540330	198480
488Grn--A	213	71.0	2.1%			
488Grn--B	0	0.0	0.0%			
488Grn--C	9884	3294.7	97.9%			
488Grn--D	0	0.0	0.0%			
488Grn--A	200	66.7	2.0%			
488Grn--B	1	0.3	0.0%			
488Grn--C	9896	3298.7	98.0%			
488Grn--D	0	0.0	0.0%			
638Red--A	36	12.0	0.4%			
638Red--B	95	31.7	0.9%			
638Red--C	9854	3284.7	97.6%			
638Red--D	112	37.3	1.1%			

Acquisition Parameters

Channel	PMT	Gain	Thresh (OR)	Subtraction
SALS	330	1.00	954	
LALS	350	1.00	414	
488Grn	285	1.00		0.00%, 0.00%, 0.00%
488Org	340	1.00	1	28.00%, 0.00%, 0.00%
488Red	520	1.00		0.00%, 0.00%, 0.00%
488DpRed	500	1.00		0.00%, 0.00%, 0.00%

Instrument Settings

Pressure	Dilution	Sample Flow	Acquisition Time
75 counts	factor of 1	15.00 ul/min	12 secs

CYT: Universal
CYTSN: 0149
Apogee Flow Systems Limited

Apogee Flow Cytometry Report
Apogee Flow Cytometer

Acquisition Date: 17 May 2021 15:18:28
Filename: Sample_210517_1351_0.fcs
Sample ID: Sample_210517_1351
Operator: A0149\ApogeeFlow
Protocol:

SALS-LALS [Peak-Peak]

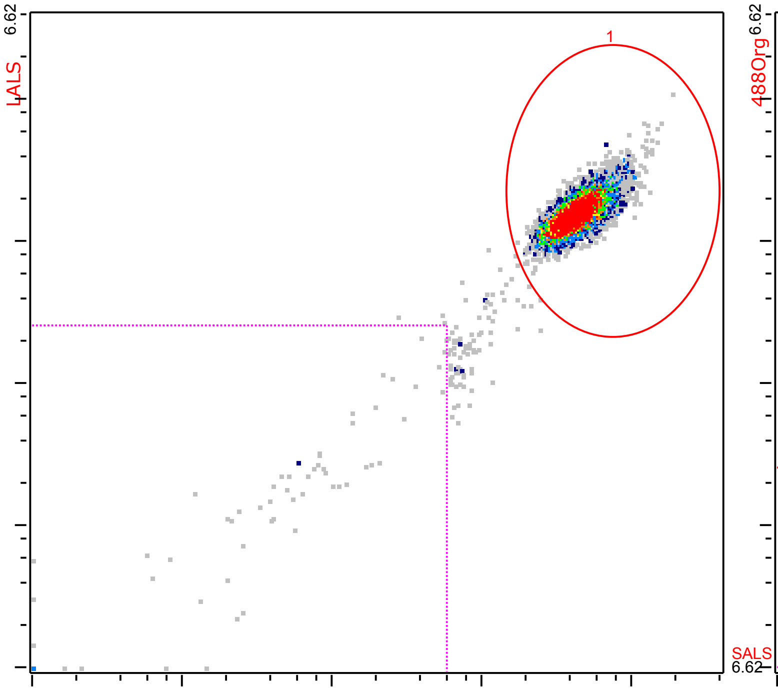

488Grn-488Org [Peak-Peak]

Gated by 1

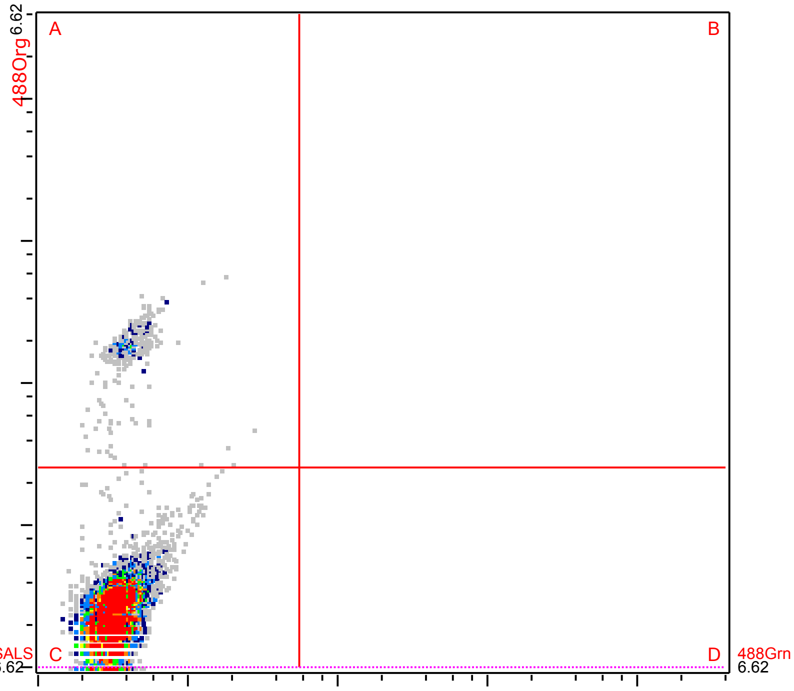

488Grn-638Red [Peak-Peak]

Gated by 1

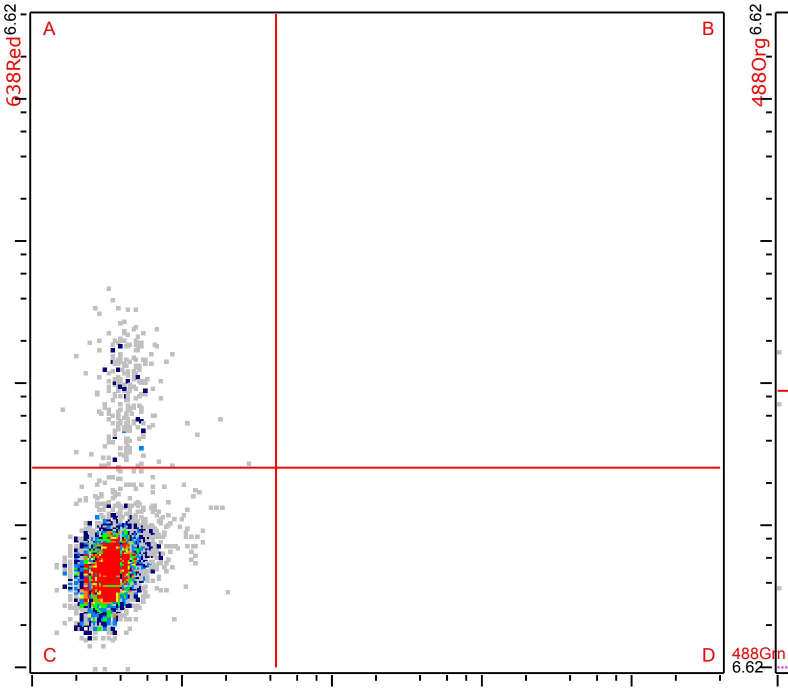

638Red-488Org [Peak-Peak]

Gated by 1

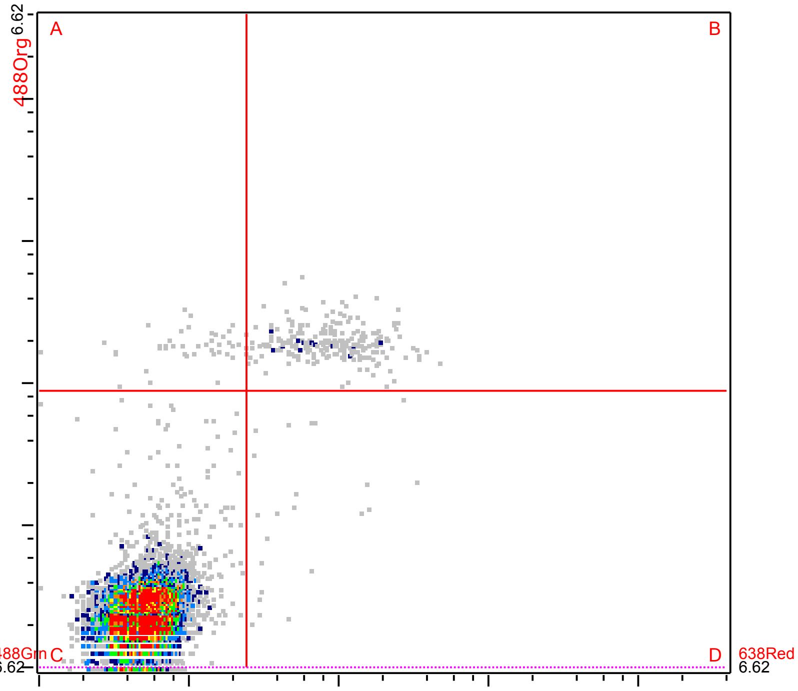

Apogee Flow Cytometry Report
Apogee Flow Cytometer

Acquisition Date:	17 May 2021 15:18:28
Filename:	Sample_210517_1351_0.fcs
Sample ID:	Sample_210517_1351
Operator:	A0149\ApogeeFlow
Protocol:	

Cytogram ROI Statistics

ROI ID	Events	Events/µl	%	Ratio	Mean X	Mean Y
1	10089	5044.5	98.5%		457249	162499
488Grn--A	319	159.5	3.2%			
488Grn--B	0	0.0	0.0%			
488Grn--C	9770	4885.0	96.8%			
488Grn--D	0	0.0	0.0%			
488Grn--A	265	132.5	2.6%			
488Grn--B	0	0.0	0.0%			
488Grn--C	9824	4912.0	97.4%			
488Grn--D	0	0.0	0.0%			
638Red--A	43	21.5	0.4%			
638Red--B	244	122.0	2.4%			
638Red--C	9780	4890.0	96.9%			
638Red--D	22	11.0	0.2%			

Acquisition Parameters

Channel	PMT	Gain	Thresh (OR)	Subtraction
SALS	330	1.00	954	
LALS	350	1.00	414	
488Grn	285	1.00		0.00%, 0.00%, 0.00%
488Org	340	1.00	1	28.00%, 0.00%, 0.00%
488Red	520	1.00		0.00%, 0.00%, 0.00%
488DpRed	500	1.00		0.00%, 0.00%, 0.00%

Instrument Settings

Pressure	Dilution	Sample Flow	Acquisition Time
75 counts	factor of 1	15.00 ul/min	8 secs

CYT: Universal
CYTSN: 0149
Apogee Flow Systems Limited

APOGEE FLOW SYSTEMS

Apogee Flow Cytometry Report
Apogee Flow Cytometer

Acquisition Date: 18 May 2021 15:46:39
Filename: Sample_210518_1391_0.fcs
Sample ID: Sample_210518_1391
Operator: A0149\ApogeeFlow
Protocol:

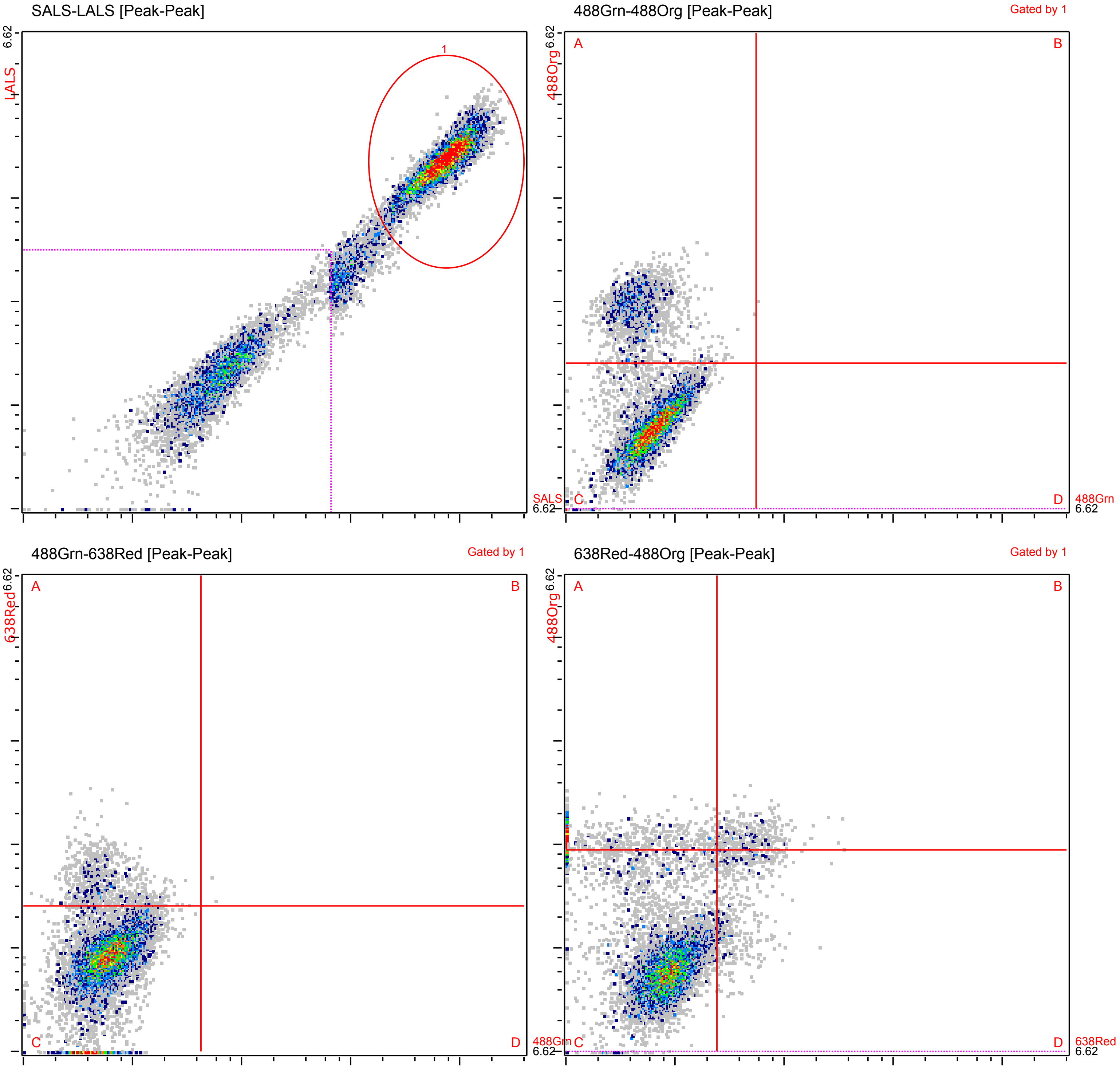

Apogee Flow Cytometry Report
Apogee Flow Cytometer

Acquisition Date:	18 May 2021 15:46:39
Filename:	Sample_210518_1391_0.fcs
Sample ID:	Sample_210518_1391
Operator:	A0149\ApogeeFlow
Protocol:	

Cytogram ROI Statistics

ROI ID	Events	Events/µl	%	Ratio	Mean X	Mean Y
1	9288	441.7	59.2%		807121	256412
488Grn--A	1748	83.1	18.8%			
488Grn--B	1	0.0	0.0%			
488Grn--C	7539	358.5	81.2%			
488Grn--D	0	0.0	0.0%			
488Grn--A	734	34.9	7.9%			
488Grn--B	2	0.1	0.0%			
488Grn--C	8552	406.7	92.1%			
488Grn--D	0	0.0	0.0%			
638Red--A	622	29.6	6.7%			
638Red--B	303	14.4	3.3%			
638Red--C	7896	375.5	85.0%			
638Red--D	467	22.2	5.0%			

Acquisition Parameters

Channel	PMT	Gain	Thresh (OR)	Subtraction
SALS	330	1.00	1075	
LALS	350	1.00	510	
488Grn	285	1.00		0.00%, 0.00%, 0.00%
488Org	340	1.00	1	28.00%, 0.00%, 0.00%
488Red	520	1.00		0.00%, 0.00%, 0.00%
488DpRed	500	1.00		0.00%, 0.00%, 0.00%

Instrument Settings

Pressure	Dilution	Sample Flow	Acquisition Time
75 counts	factor of 1	7.51 ul/min	168 secs

CYT: Universal
CYTSN: 0149
Apogee Flow Systems Limited

APOGEE FLOW SYSTEMS

Apogee Flow Cytometry Report
Apogee Flow Cytometer

Acquisition Date:	19 May 2021 16:13:01
Filename:	Sample_210519_1405_0.fcs
Sample ID:	Sample_210519_1405
Operator:	A0149\ApogeeFlow
Protocol:	

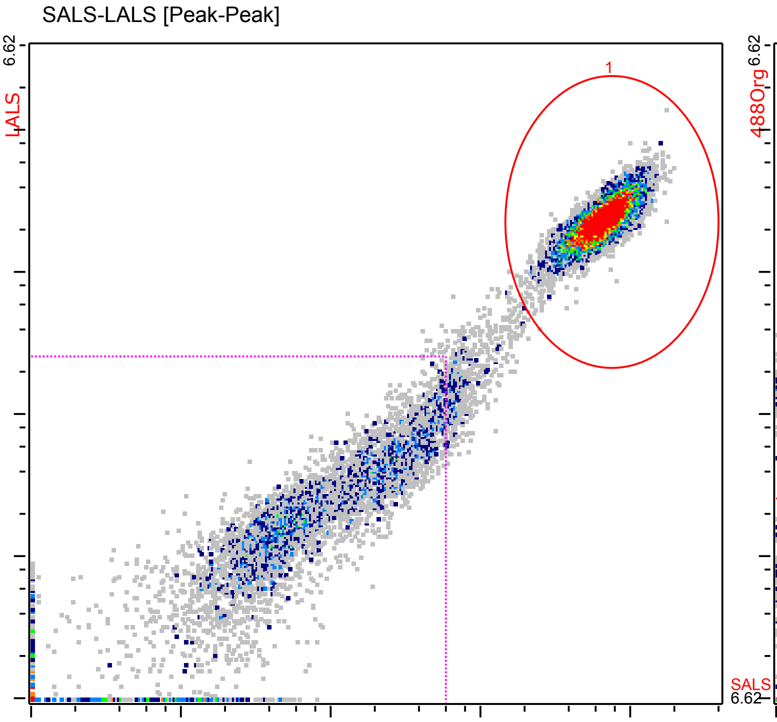

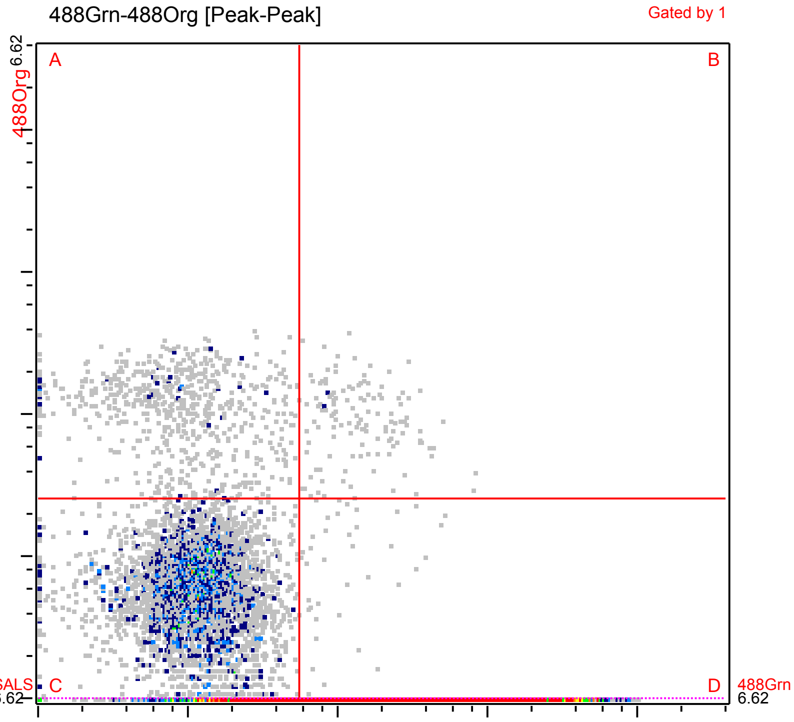

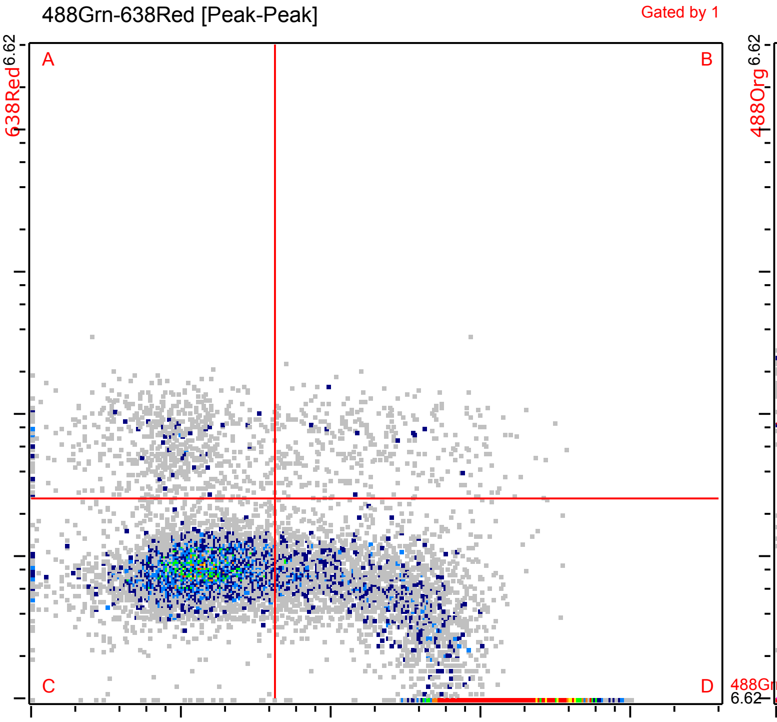

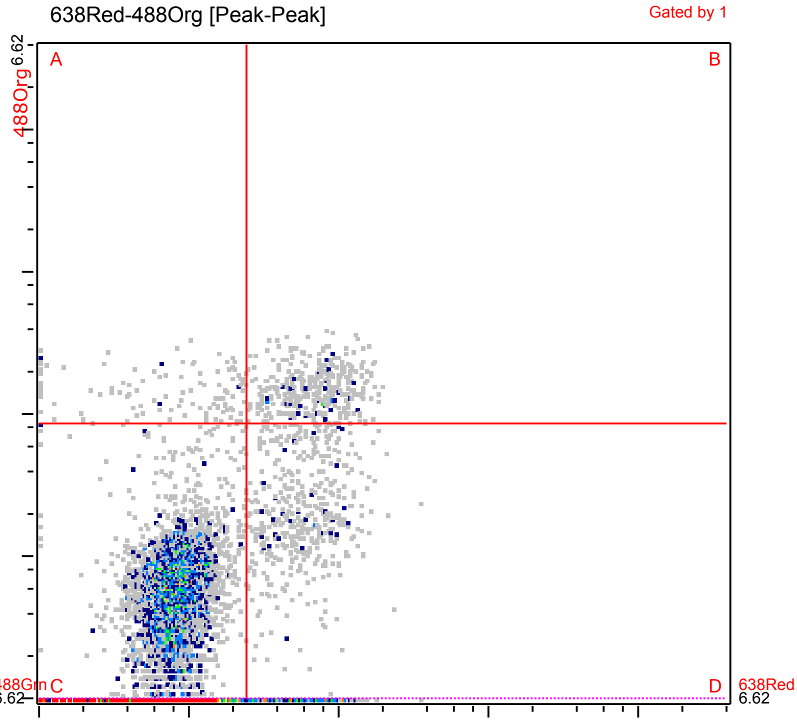

Apogee Flow Cytometry Report
Apogee Flow Cytometer

Acquisition Date:	19 May 2021 16:13:01
Filename:	Sample_210519_1405_0.fcs
Sample ID:	Sample_210519_1405
Operator:	A0149\ApogeeFlow
Protocol:	

Cytogram ROI Statistics

ROI ID	Events	Events/µl	%	Ratio	Mean X	Mean Y
1	10251	2928.9	39.8%		692909	245210
488Grn--A	651	186.0	6.4%			
488Grn--B	129	36.9	1.3%			
488Grn--C	5491	1568.9	53.6%			
488Grn--D	3980	1137.1	38.8%			
488Grn--A	720	205.7	7.0%			
488Grn--B	334	95.4	3.3%			
488Grn--C	5108	1459.4	49.8%			
488Grn--D	4089	1168.3	39.9%			
638Red--A	117	33.4	1.1%			
638Red--B	399	114.0	3.9%			
638Red--C	9068	2590.9	88.5%			
638Red--D	667	190.6	6.5%			

Acquisition Parameters

Channel	PMT	Gain	Thresh (OR)	Subtraction
SALS	330	1.00	954	
LALS	350	1.00	414	
488Grn	285	1.00		0.00%, 0.00%, 0.00%
488Org	340	1.00	1	28.00%, 0.00%, 0.00%
488Red	520	1.00		0.00%, 0.00%, 0.00%
488DpRed	500	1.00		0.00%, 0.00%, 0.00%

Instrument Settings

Pressure	Dilution	Sample Flow	Acquisition Time
75 counts	factor of 1	15.00 ul/min	14 secs

CYT: Universal
CYTSN: 0149
Apogee Flow Systems Limited

APOGEE FLOW SYSTEMS

Apogee Flow Cytometry Report
Apogee Flow Cytometer

Acquisition Date: 16 April 2021 16:06:07
Filename: Sample_210416_1215_0.fcs
Sample ID: Sample_210416_1215
Operator: A0149\ApogeeFlow
Protocol:

SALS-LALS [Peak-Peak]

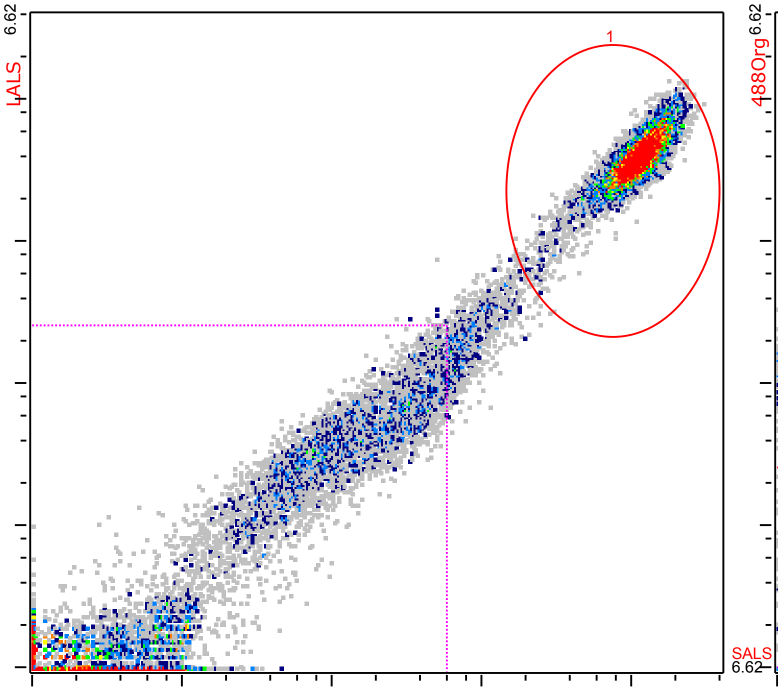

488Grn-488Org [Peak-Peak]

Gated by 1

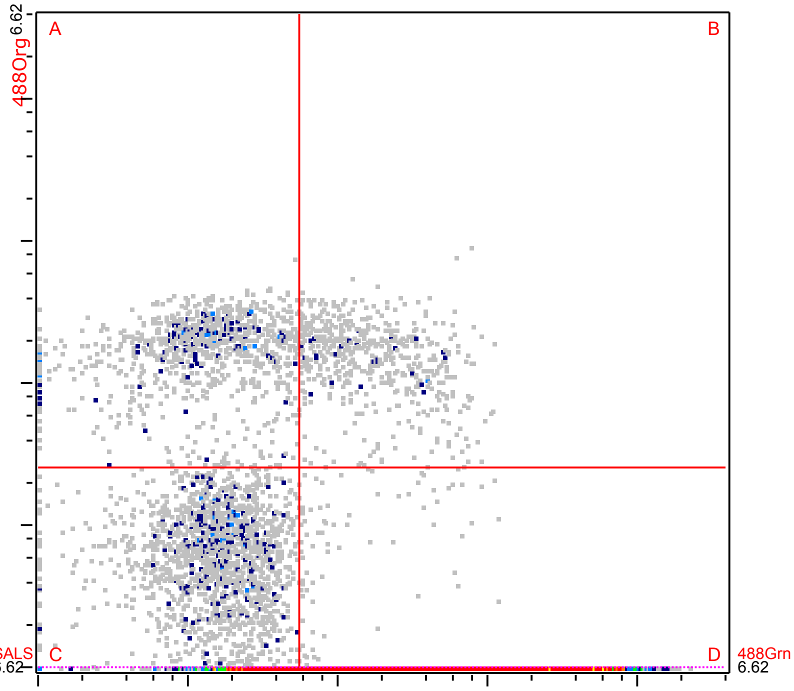

488Grn-638Red [Peak-Peak]

Gated by 1

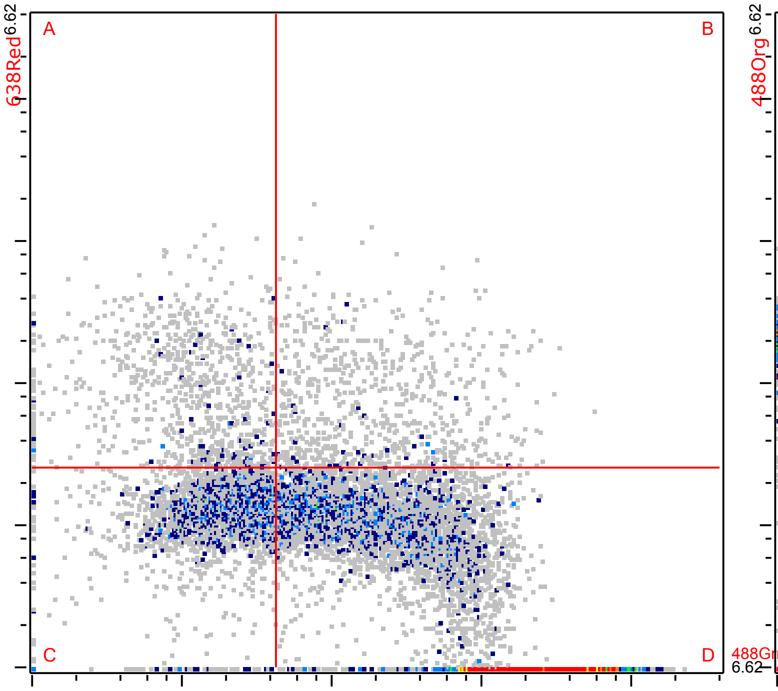

638Red-488Org [Peak-Peak]

Gated by 1

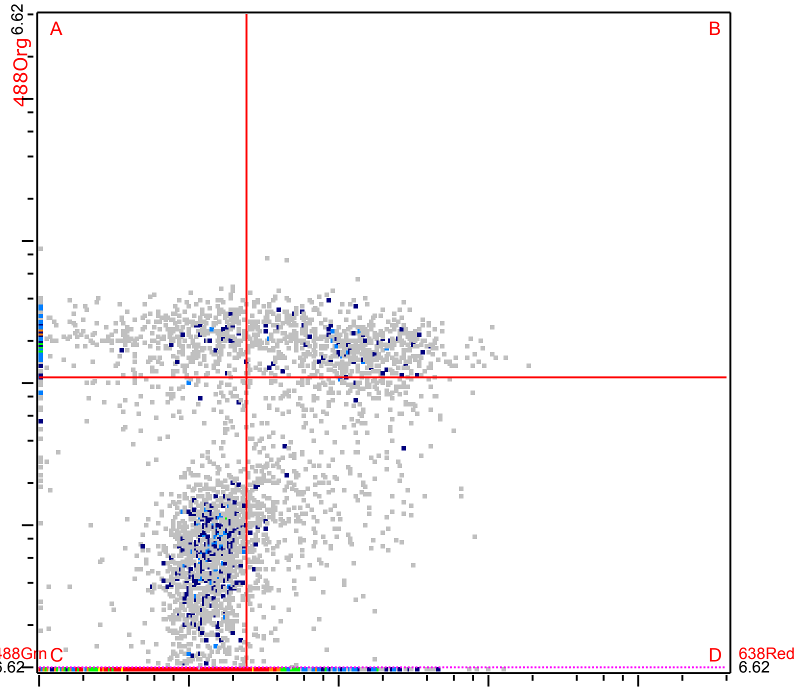

Apogee Flow Cytometry Report
Apogee Flow Cytometer

Acquisition Date:	16 April 2021 16:06:07
Filename:	Sample_210416_1215_0.fcs
Sample ID:	Sample_210416_1215
Operator:	A0149\ApogeeFlow
Protocol:	

Cytogram ROI Statistics

ROI ID	Events	Events/µl	%	Ratio	Mean X	Mean Y
1	10049	788.2	26.9%		1186549	415659
488Grn--A	1020	80.0	10.2%			
488Grn--B	562	44.1	5.6%			
488Grn--C	2763	216.7	27.5%			
488Grn--D	5704	447.4	56.8%			
488Grn--A	897	70.4	8.9%			
488Grn--B	764	59.9	7.6%			
488Grn--C	2426	190.3	24.1%			
488Grn--D	5962	467.6	59.3%			
638Red--A	534	41.9	5.3%			
638Red--B	695	54.5	6.9%			
638Red--C	7810	612.5	77.7%			
638Red--D	1010	79.2	10.1%			

Acquisition Parameters

Channel	PMT	Gain	Thresh (OR)	Subtraction
SALS	330	1.00	954	
LALS	350	1.00	414	
488Grn	285	1.00		0.00%, 0.00%, 0.00%
488Org	340	1.00	1	28.00%, 0.00%, 0.00%
488Red	520	1.00		0.00%, 0.00%, 0.00%
488DpRed	500	1.00		0.00%, 0.00%, 0.00%

Instrument Settings

Pressure	Dilution	Sample Flow	Acquisition Time
75 counts	factor of 1	15.00 ul/min	51 secs

CYT: Universal

CYTSN: 0149

Apogee Flow Systems Limited
