# Supplementary material for: Circular RNA CDR1as Alleviates Cisplatin-Based Chemoresistance by Suppressing MiR-1299 in Ovarian Cancer
Source: Front Genet. 2022 Jan 26;12:815448. doi: 10.3389/fgene.2021.815448 (PMC8826532; doi:10.3389/fgene.2021.815448)
Supplement: Supplementary file 3 [file DataSheet4.PDF]

Apogee Flow Cytometry Report
Apogee Flow Cytometer

Acquisition Date: 17 May 2021 14:32:10
Filename: Sample_210517_1336_0.fcs
Sample ID: Sample_210517_1336
Operator: A0149\ApogeeFlow
Protocol:
AutoSaved Original: TRUE

SALS-LALS [Peak-Peak]

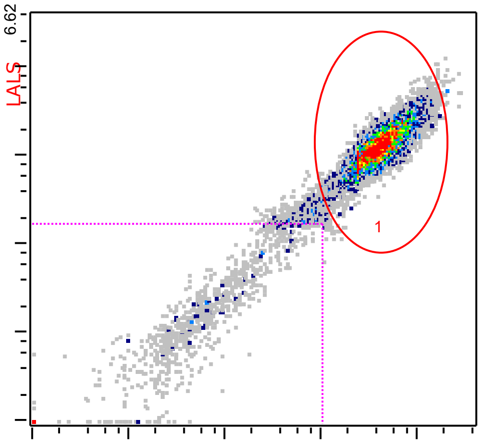

638Red-488Org [Peak-Peak]

Gated by 1

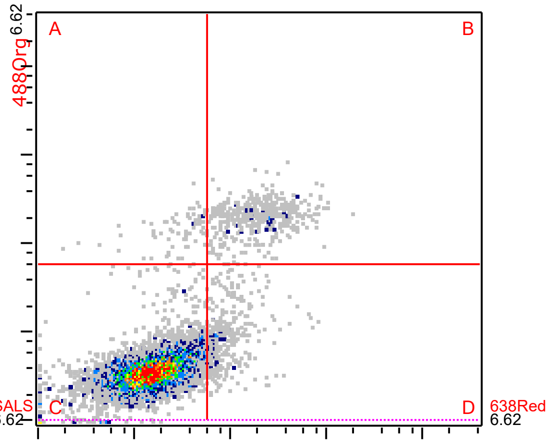

Cytogram ROI Statistics

ROI ID	Events	Events/µl	%	Ratio	Mean X	Mean Y
1	12303	2046.0	89.7%		471373 (Ari)	144129 (Ari)
638Red--A	75	12.5	0.6%			
638Red--B	439	73.0	3.6%			
638Red--C	11428	1900.4	92.9%			
638Red--D	361	60.0	2.9%			

Acquisition Parameters

Channel	PMT	Gain	Thresh (OR)	Subtraction
SALS	330	1.00	1731	
LALS	350	1.00	265	
488Grn	285	1.00		0.00%, 0.00%, 0.00%
488Org	340	1.00	1	0.00%, 0.00%, 0.00%
488Red	520	1.00		0.00%, 0.00%, 0.00%
488DpRed	500	1.00		0.00%, 0.00%, 0.00%

Instrument Settings

Pressure	Dilution	Sample Flow	Acquisition Time
50 counts	factor of 1	9.02 ul/min	40 secs

CYTOMETER UNCALIBRATED

CYT: Universal
CYTSN: 0149
Apogee Flow Systems Limited

APOGEE FLOW SYSTEMS

Apogee Flow Cytometry Report
Apogee Flow Cytometer

Acquisition Date:	17 May 2021 14:37:32
Filename:	Sample_210517_1338_0.fcs
Sample ID:	Sample_210517_1338
Operator:	A0149\ApogeeFlow
Protocol:	
AutoSaved Original:	TRUE

SALS-LALS [Peak-Peak]

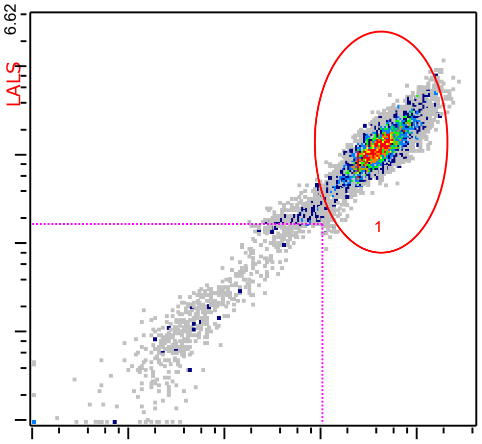

638Red-488Org [Peak-Peak]

Gated by 1

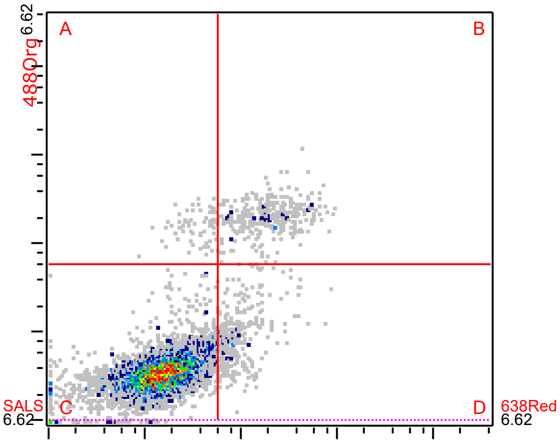

Cytogram ROI Statistics

ROI ID	Events	Events/µl	%	Ratio	Mean X	Mean Y
1	10026	1905.5	90.7%		466160 (Ari)	140991 (Ari)
638Red--A	69	13.1	0.7%			
638Red--B	316	60.1	3.2%			
638Red--C	9326	1772.4	93.0%			
638Red--D	315	59.9	3.1%			

Acquisition Parameters

Channel	PMT	Gain	Thresh (OR)	Subtraction
SALS	330	1.00	1731	
LALS	350	1.00	265	
488Grn	285	1.00		0.00%, 0.00%, 0.00%
488Org	340	1.00	1	0.00%, 0.00%, 0.00%
488Red	520	1.00		0.00%, 0.00%, 0.00%
488DpRed	500	1.00		0.00%, 0.00%, 0.00%

Instrument Settings

Pressure	Dilution	Sample Flow	Acquisition Time
50 counts	factor of 1	9.02 ul/min	35 secs

CYTOMETER UNCALIBRATED

CYT: Universal

CYTSN: 0149

Apogee Flow Systems Limited

Apogee Flow Cytometry Report
Apogee Flow Cytometer

Acquisition Date:	17 May 2021 14:40:04
Filename:	Sample_210517_1339_0.fcs
Sample ID:	Sample_210517_1339
Operator:	A0149\ApogeeFlow
Protocol:	
AutoSaved Original:	TRUE

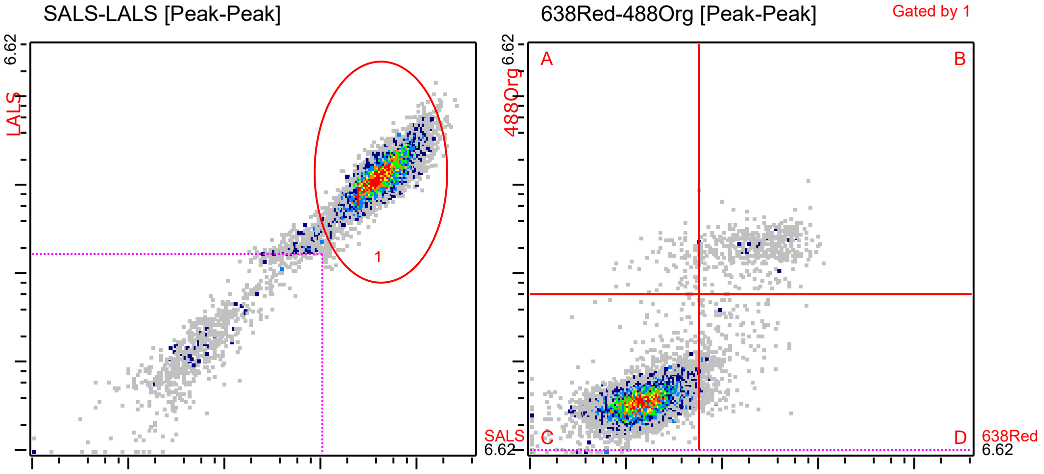

Cytogram ROI Statistics

ROI ID	Events	Events/µl	%	Ratio	Mean X	Mean Y
1	10001	1900.7	90.5%		474141 (Ari)	144498 (Ari)
638Red--A	73	13.9	0.7%			
638Red--B	389	73.9	3.9%			
638Red--C	9276	1762.9	92.8%			
638Red--D	263	50.0	2.6%			

Acquisition Parameters

Channel	PMT	Gain	Thresh (OR)	Subtraction
SALS	330	1.00	1731	
LALS	350	1.00	265	
488Grn	285	1.00		0.00%, 0.00%, 0.00%
488Org	340	1.00	1	0.00%, 0.00%, 0.00%
488Red	520	1.00		0.00%, 0.00%, 0.00%
488DpRed	500	1.00		0.00%, 0.00%, 0.00%

Instrument Settings

Pressure	Dilution	Sample Flow	Acquisition Time
50 counts	factor of 1	9.02 ul/min	35 secs

CYTOMETER UNCALIBRATED

CYT: Universal
CYTSN: 0149
Apogee Flow Systems Limited

APOGEE FLOW SYSTEMS

Apogee Flow Cytometry Report
Apogee Flow Cytometer

Acquisition Date:	17 May 2021 14:42:31
Filename:	Sample_210517_1340_0.fcs
Sample ID:	Sample_210517_1340
Operator:	A0149\ApogeeFlow
Protocol:	
AutoSaved Original:	TRUE

SALS-LALS [Peak-Peak]

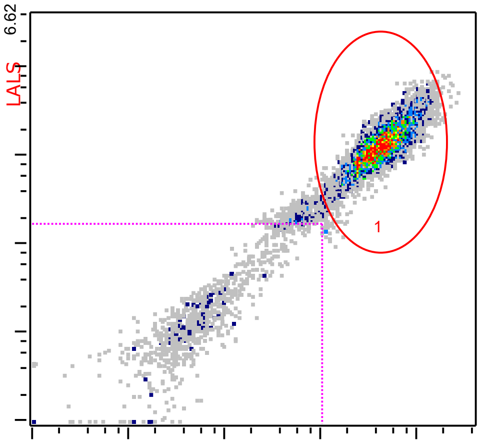

638Red-488Org [Peak-Peak]

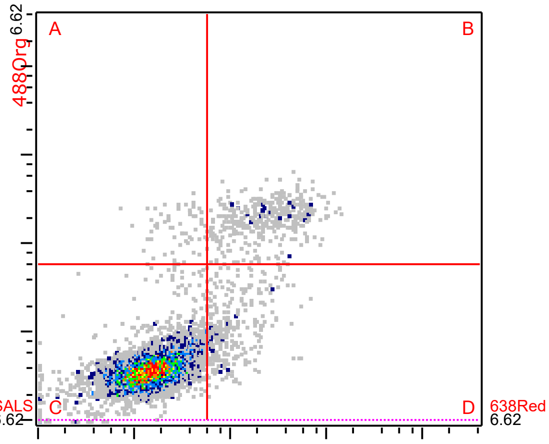

Cytogram ROI Statistics

ROI ID	Events	Events/µl	%	Ratio	Mean X	Mean Y
1	10002	1386.1	88.4%		482113 (Ari)	147028 (Ari)
638Red--A	63	8.7	0.6%			
638Red--B	397	55.0	4.0%			
638Red--C	9241	1280.6	92.4%			
638Red--D	301	41.7	3.0%			

Acquisition Parameters

Channel	PMT	Gain	Thresh (OR)	Subtraction
SALS	330	1.00	1731	
LALS	350	1.00	265	
488Grn	285	1.00		0.00%, 0.00%, 0.00%
488Org	340	1.00	1	0.00%, 0.00%, 0.00%
488Red	520	1.00		0.00%, 0.00%, 0.00%
488DpRed	500	1.00		0.00%, 0.00%, 0.00%

Instrument Settings

Pressure	Dilution	Sample Flow	Acquisition Time
50 counts	factor of 1	9.02 ul/min	48 secs

CYTOMETER UNCALIBRATED

CYT: Universal
CYTSN: 0149
Apogee Flow Systems Limited

Apogee Flow Cytometry Report
Apogee Flow Cytometer

Acquisition Date:	17 May 2021 14:58:25
Filename:	Sample_210517_1346_0.fcs
Sample ID:	Sample_210517_1346
Operator:	A0149\ApogeeFlow
Protocol:	
AutoSaved Original:	TRUE

SALS-LALS [Peak-Peak]

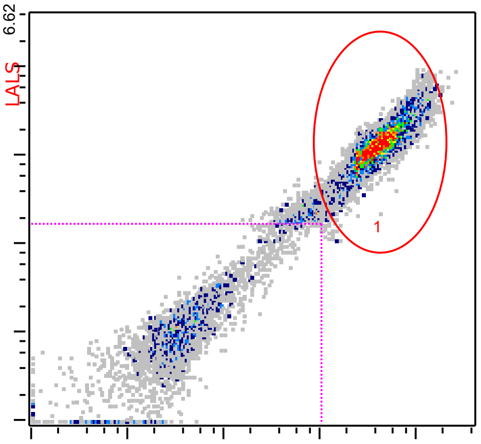

638Red-488Org [Peak-Peak]

Gated by 1

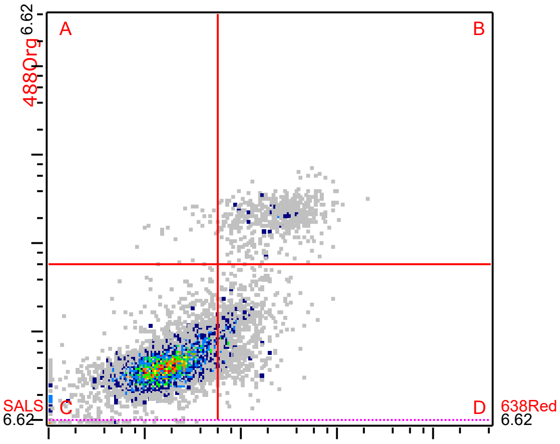

Cytogram ROI Statistics

ROI ID	Events	Events/µl	%	Ratio	Mean X	Mean Y
1	10019	1211.7	73.1%		469495 (Ari)	152902 (Ari)
638Red--A	27	3.3	0.3%			
638Red--B	466	56.4	4.7%			
638Red--C	8787	1062.7	87.7%			
638Red--D	739	89.4	7.4%			

Acquisition Parameters

Channel	PMT	Gain	Thresh (OR)	Subtraction
SALS	330	1.00	1731	
LALS	350	1.00	265	
488Grn	285	1.00		0.00%, 0.00%, 0.00%
488Org	340	1.00	1	0.00%, 0.00%, 0.00%
488Red	520	1.00		0.00%, 0.00%, 0.00%
488DpRed	500	1.00		0.00%, 0.00%, 0.00%

Instrument Settings

Pressure	Dilution	Sample Flow	Acquisition Time
50 counts	factor of 1	9.02 ul/min	55 secs

CYTOMETER UNCALIBRATED

CYT: Universal
CYTSN: 0149
Apogee Flow Systems Limited

APOGEE FLOW SYSTEMS

Apogee Flow Cytometry Report
Apogee Flow Cytometer

Acquisition Date:	19 May 2021 16:17:22
Filename:	Sample_210519_1407_0.fcs
Sample ID:	Sample_210519_1407
Operator:	A0149\ApogeeFlow
Protocol:	
AutoSaved Original:	TRUE

SALS-LALS [Peak-Peak]

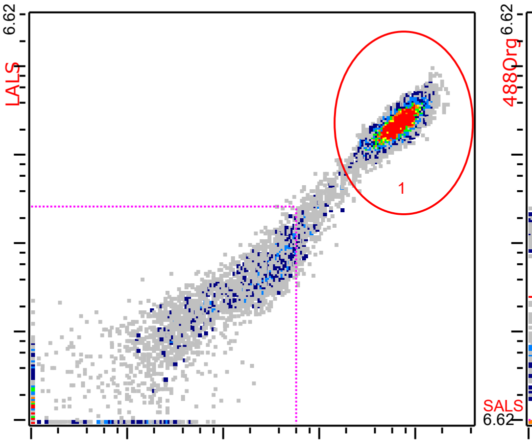

488Grn-488Org [Peak-Peak] Gated by 1

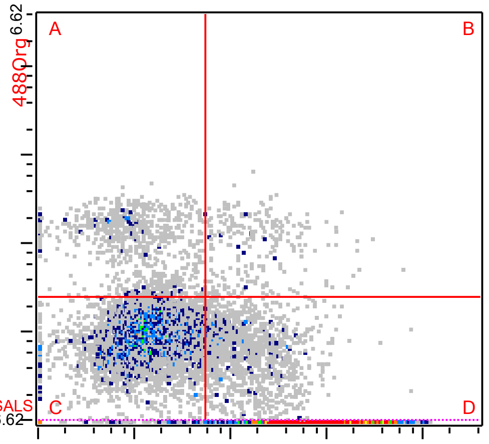

488Grn-638Red [Peak-Peak] Gated by 1

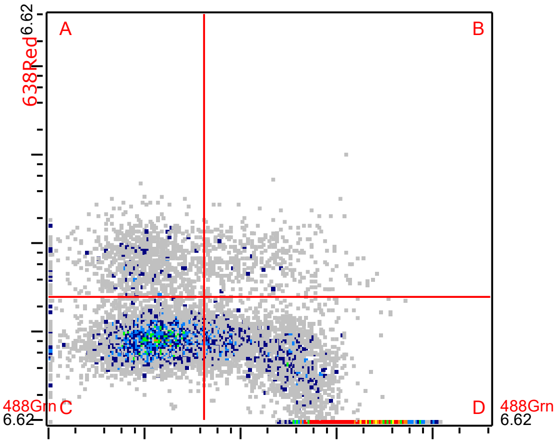

638Red-488Org [Peak-Peak] Gated by 1

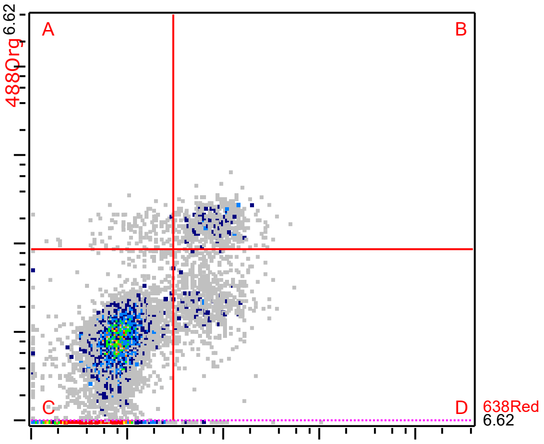

Cytogram ROI Statistics

ROI ID	Events	Events/µl	%	Ratio	Mean X	Mean Y
1	10131	4052.4	45.0%		709907 (Ari)	243970 (Ari)
488Grn--A	824	329.6	8.1%			
488Grn--B	241	96.4	2.4%			
488Grn--C	5470	2188.0	54.0%			
488Grn--D	3596	1438.4	35.5%			
488Grn--A	935	374.0	9.2%			
488Grn--B	386	154.4	3.8%			
488Grn--C	4992	1996.8	49.3%			
488Grn--D	3818	1527.2	37.7%			
638Red--A	124	49.6	1.2%			
638Red--B	538	215.2	5.3%			
638Red--C	8792	3516.8	86.8%			
638Red--D	677	270.8	6.7%			

Acquisition Parameters

Channel	PMT	Gain	Thresh (OR)	Subtraction
SALS	330	1.00	954	
LALS	350	1.00	414	
488Grn	285	1.00		0.00%, 0.00%, 0.00%
488Org	340	1.00	1	28.00%, 0.00%, 0.00%
488Red	520	1.00		0.00%, 0.00%, 0.00%
488DpRed	500	1.00		0.00%, 0.00%, 0.00%

Instrument Settings

Pressure	Dilution	Sample Flow	Acquisition Time
75 counts	factor of 1	15.00 ul/min	10 secs

CYTOMETER UNCALIBRATED

CYT: Universal
CYTSN: 0149
Apogee Flow Systems Limited

APOGEE FLOW SYSTEMS

Apogee Flow Cytometry Report
Apogee Flow Cytometer

Acquisition Date:	17 May 2021 15:20:24
Filename:	Sample_210517_1352_0.fcs
Sample ID:	Sample_210517_1352
Operator:	A0149\ApogeeFlow
Protocol:	

SALS-LALS [Peak-Peak]

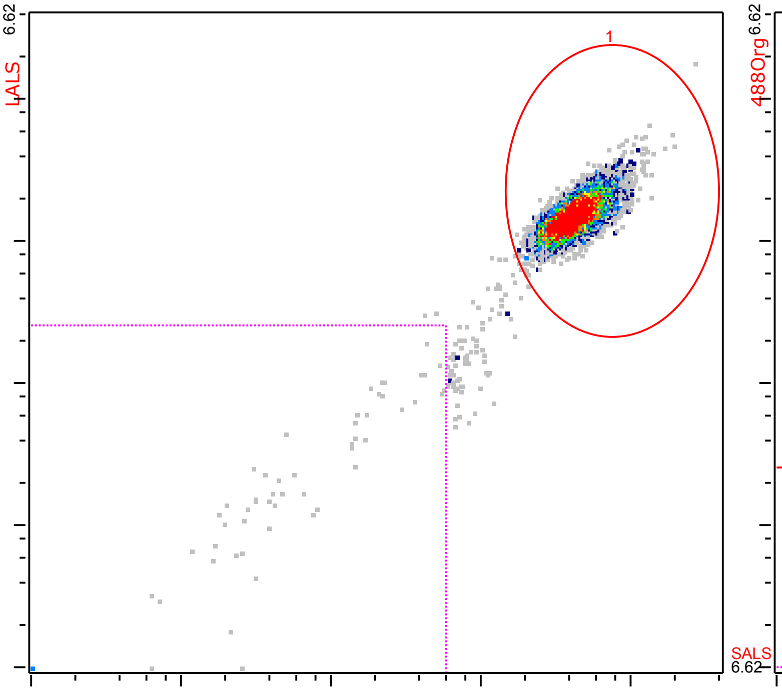

488Grn-488Org [Peak-Peak]

Gated by 1

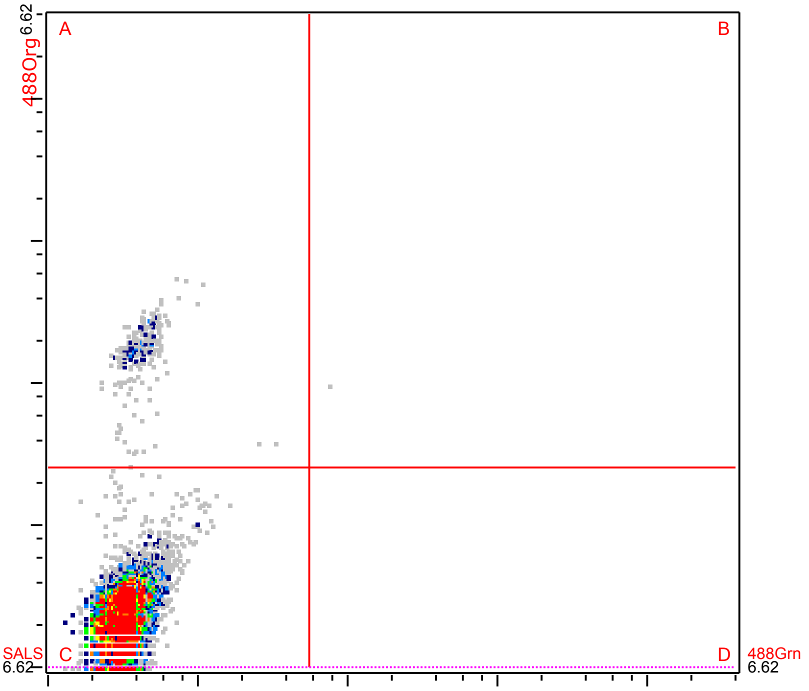

488Grn-638Red [Peak-Peak]

Gated by 1

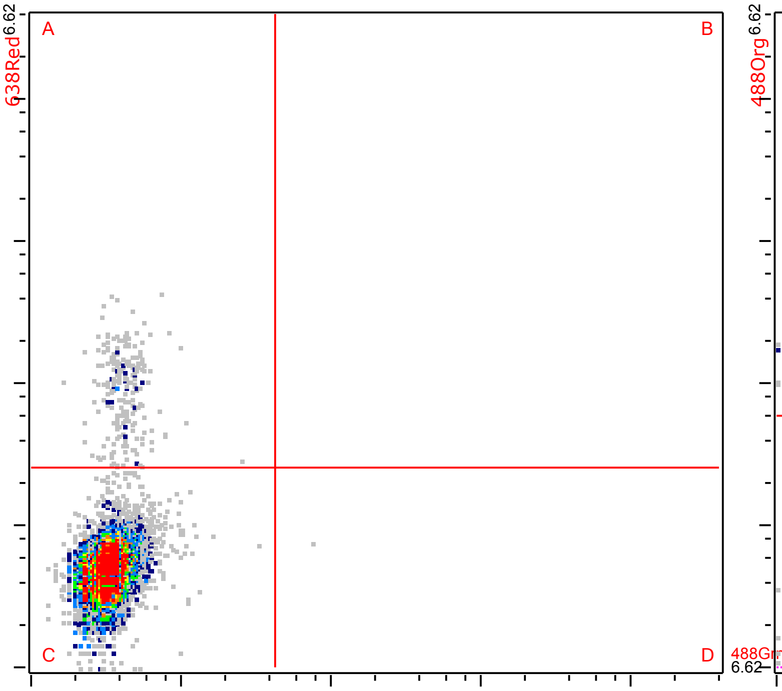

638Red-488Org [Peak-Peak]

Gated by 1

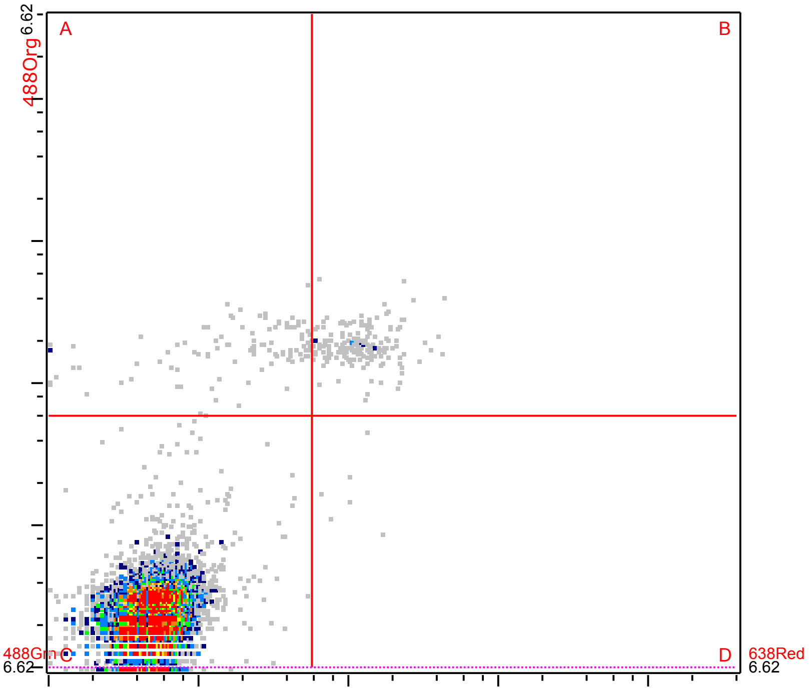

Apogee Flow Cytometry Report
Apogee Flow Cytometer

Acquisition Date:	17 May 2021 15:20:24
Filename:	Sample_210517_1352_0.fcs
Sample ID:	Sample_210517_1352
Operator:	A0149\ApogeeFlow
Protocol:	

Cytogram ROI Statistics

ROI ID	Events	Events/µl	%	Ratio	Mean X	Mean Y
1	10094	5768.0	98.6%		448779	157106
488Grn--A	284	162.3	2.8%			
488Grn--B	1	0.6	0.0%			
488Grn--C	9809	5605.1	97.2%			
488Grn--D	0	0.0	0.0%			
488Grn--A	238	136.0	2.4%			
488Grn--B	0	0.0	0.0%			
488Grn--C	9855	5631.4	97.6%			
488Grn--D	1	0.6	0.0%			
638Red--A	100	57.1	1.0%			
638Red--B	170	97.1	1.7%			
638Red--C	9818	5610.3	97.3%			
638Red--D	6	3.4	0.1%			

Acquisition Parameters

Channel	PMT	Gain	Thresh (OR)	Subtraction
SALS	330	1.00	954	
LALS	350	1.00	414	
488Grn	285	1.00		0.00%, 0.00%, 0.00%
488Org	340	1.00	1	28.00%, 0.00%, 0.00%
488Red	520	1.00		0.00%, 0.00%, 0.00%
488DpRed	500	1.00		0.00%, 0.00%, 0.00%

Instrument Settings

Pressure	Dilution	Sample Flow	Acquisition Time
75 counts	factor of 1	15.00 ul/min	7 secs

CYT: Universal
CYTSN: 0149
Apogee Flow Systems Limited

Apogee Flow Cytometry Report
Apogee Flow Cytometer

Acquisition Date:	18 May 2021 14:55:20
Filename:	Sample_210518_1371_0.fcs
Sample ID:	Sample_210518_1371
Operator:	A0149\ApogeeFlow
Protocol:	

SALS-LALS [Peak-Peak]

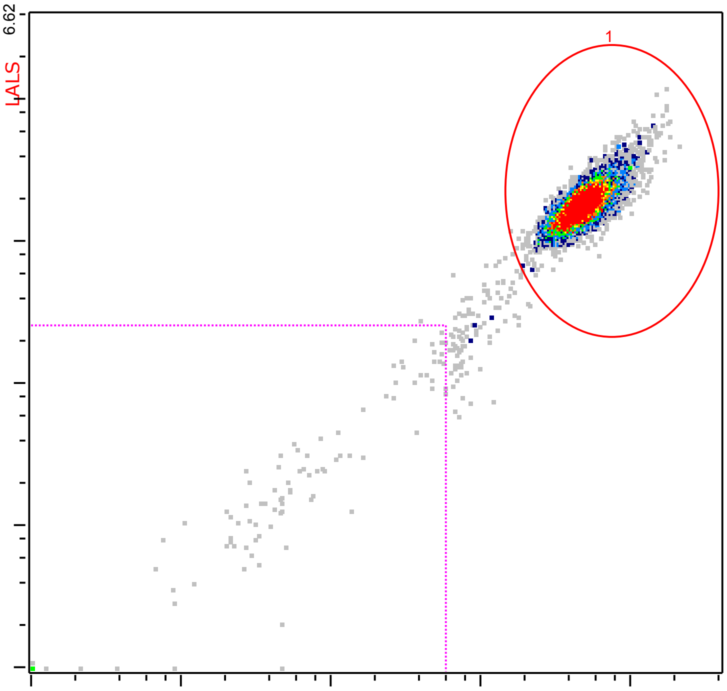

488Grn-488Org [Peak-Peak]

Gated by 1

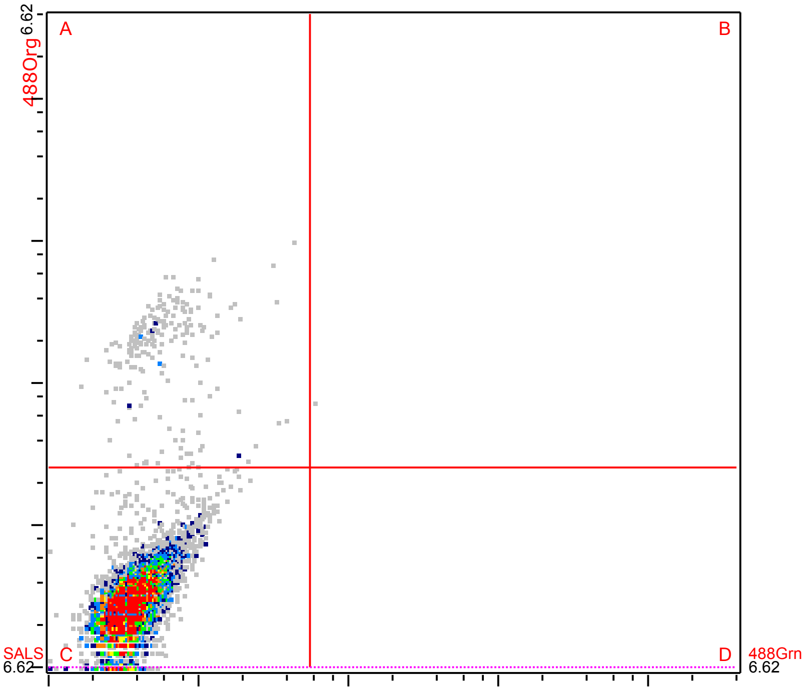

488Grn-638Red [Peak-Peak]

Gated by 1

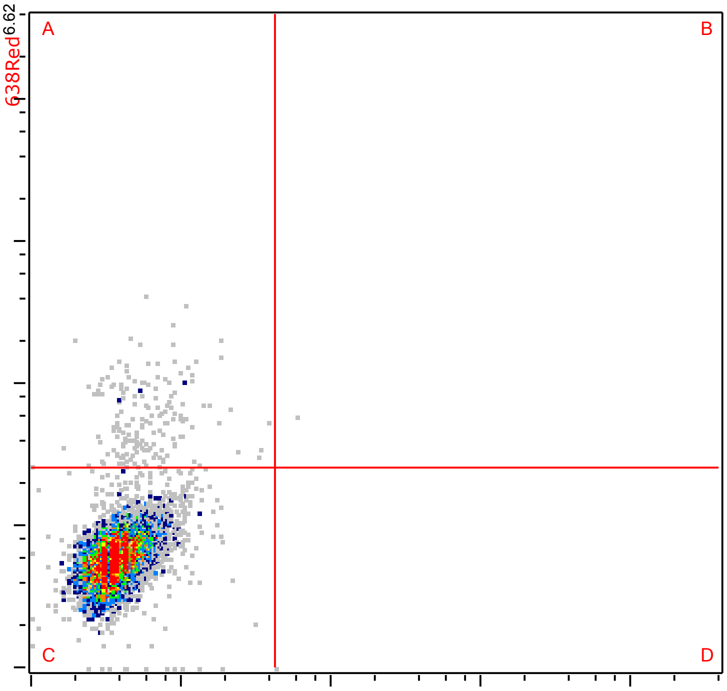

638Red-488Org [Peak-Peak]

Gated by 1

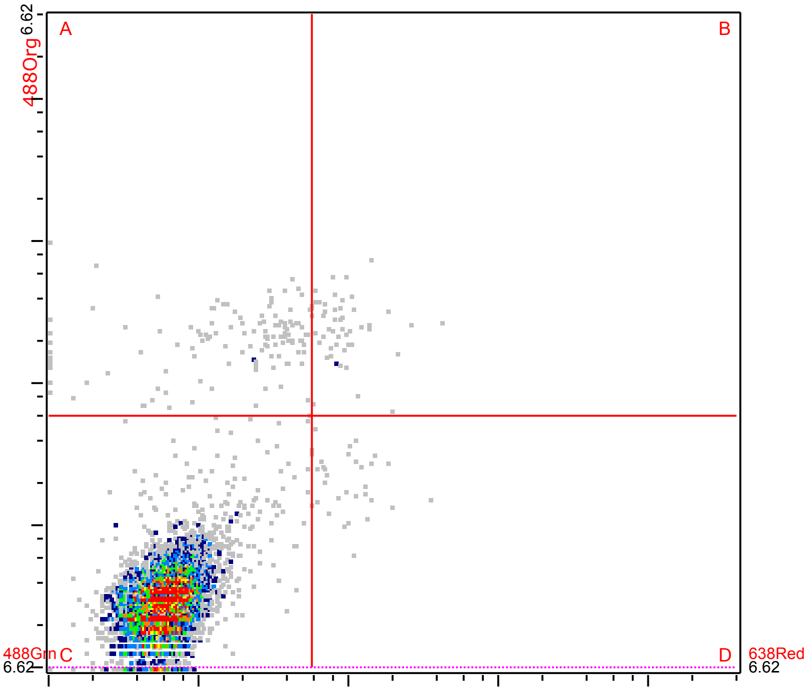

Apogee Flow Cytometry Report
Apogee Flow Cytometer

Acquisition Date:	18 May 2021 14:55:20
Filename:	Sample_210518_1371_0.fcs
Sample ID:	Sample_210518_1371
Operator:	A0149\ApogeeFlow
Protocol:	

Cytogram ROI Statistics

ROI ID	Events	Events/µl	%	Ratio	Mean X	Mean Y
1	10005	4002.0	98.0%		517147	190257
488Grn--A	204	81.6	2.0%			
488Grn--B	1	0.4	0.0%			
488Grn--C	9800	3920.0	98.0%			
488Grn--D	0	0.0	0.0%			
488Grn--A	170	68.0	1.7%			
488Grn--B	1	0.4	0.0%			
488Grn--C	9833	3933.2	98.3%			
488Grn--D	1	0.4	0.0%			
638Red--A	126	50.4	1.3%			
638Red--B	48	19.2	0.5%			
638Red--C	9802	3920.8	98.0%			
638Red--D	29	11.6	0.3%			

Acquisition Parameters

Channel	PMT	Gain	Thresh (OR)	Subtraction
SALS	330	1.00	954	
LALS	350	1.00	414	
488Grn	285	1.00		0.00%, 0.00%, 0.00%
488Org	340	1.00	1	28.00%, 0.00%, 0.00%
488Red	520	1.00		0.00%, 0.00%, 0.00%
488DpRed	500	1.00		0.00%, 0.00%, 0.00%

Instrument Settings

Pressure	Dilution	Sample Flow	Acquisition Time
75 counts	factor of 1	15.00 ul/min	10 secs

CYT: Universal
CYTSN: 0149
Apogee Flow Systems Limited

APOGEE
FLOW SYSTEMS
