# Supplementary material for: Circular RNA CDR1as Alleviates Cisplatin-Based Chemoresistance by Suppressing MiR-1299 in Ovarian Cancer
Source: Front Genet. 2022 Jan 26;12:815448. doi: 10.3389/fgene.2021.815448 (PMC8826532; doi:10.3389/fgene.2021.815448)

TRANSWELL

SKOV3

Sh-CDR1as control SC+CDDP s+cis

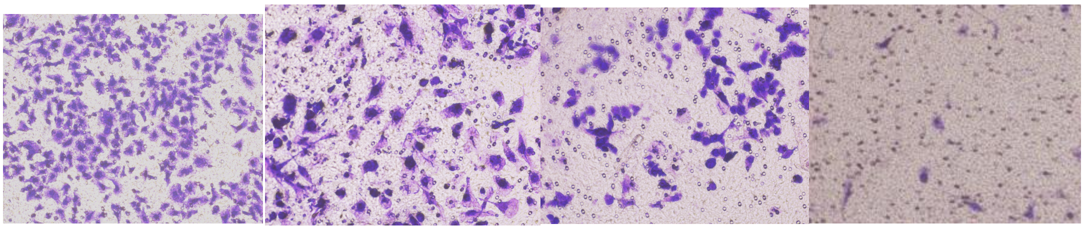

HO8910

Sh-CDR1as control SC+CDDP s+cis

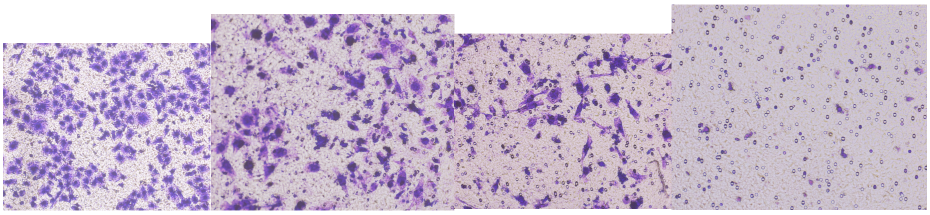

DDP

SKOV3DDP

DCTL DC D+CDDP DC+CDDP

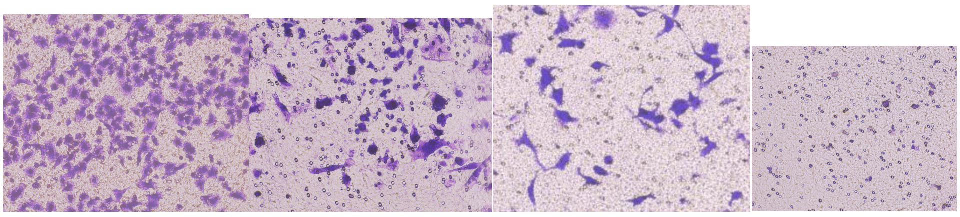

8910DDP

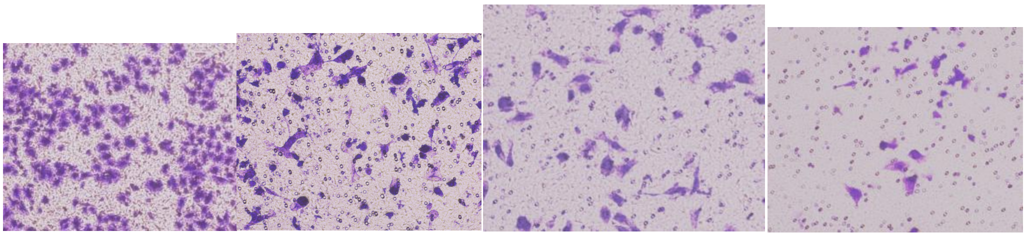

Supplement: Supplementary file 4 [file DataSheet6.PDF]
