# Supplementary material for: Circular RNA CDR1as Alleviates Cisplatin-Based Chemoresistance by Suppressing MiR-1299 in Ovarian Cancer
Source: Front Genet. 2022 Jan 26;12:815448. doi: 10.3389/fgene.2021.815448 (PMC8826532; doi:10.3389/fgene.2021.815448)
Supplement: Supplementary file 5 [file Table1.DOCX]

Supplementary Material

## 1.2 Supplementary Tables

**Supplementary Table 1** The sequences of CDR1as knock-down lentivirus.

| **Gene**  **sequences (5`-3`)** |
| --- |
| shRNA1 Top stand: GATCCGATATCCAGGGTTTCCGATGGCACCTTTCAAGAG  AAGGTGCCATCGGAAACCCTGGATATTTTTTTG  Bottom strand: AATTCAAAAAAATATCCAGGGTTTCCGATGGCACCTT  CTCTTGAAAGGTGCCATCGGAAACCCTGGATATCG  shRNA2 Top stand: GATCCGTGTCTGCAATATCCAGGGTTTCCGTTCAAGAG  ACGGAAACCCTGGATATTGCAGACACTTTTTTG  Bottom strand: AATTCAAAAAAGTGTCTGCAATATCCAGGGTTTCCGT  CTCTTGAACGGAAACCCTGGATATTGCAGACACG  shRNA3 Top stand: GATCCGTGCAATATCCAGGGTTTCCGATGGCTTCAAGAG  AGCCATCGGAAACCCTGGATATTGCATTTTTTG  Bottom strand: AATTCAAAAAATGCAATATCCAGGGTTTCCGATGGCT  CTCTTGAAGCCATCGGAAACCCTGGATATTGCACG |
