# Supplementary material for: Circular RNA CDR1as Alleviates Cisplatin-Based Chemoresistance by Suppressing MiR-1299 in Ovarian Cancer
Source: Front Genet. 2022 Jan 26;12:815448. doi: 10.3389/fgene.2021.815448 (PMC8826532; doi:10.3389/fgene.2021.815448)
Supplement: Supplementary file 6 [file DataSheet3.PDF]

Apogee Flow Cytometry Report
Apogee Flow Cytometer

Acquisition Date:	17 May 2021 15:41:41
Filename:	Sample_210517_1361_0.fcs
Sample ID:	Sample_210517_1361
Operator:	A0149\ApogeeFlow
Protocol:	

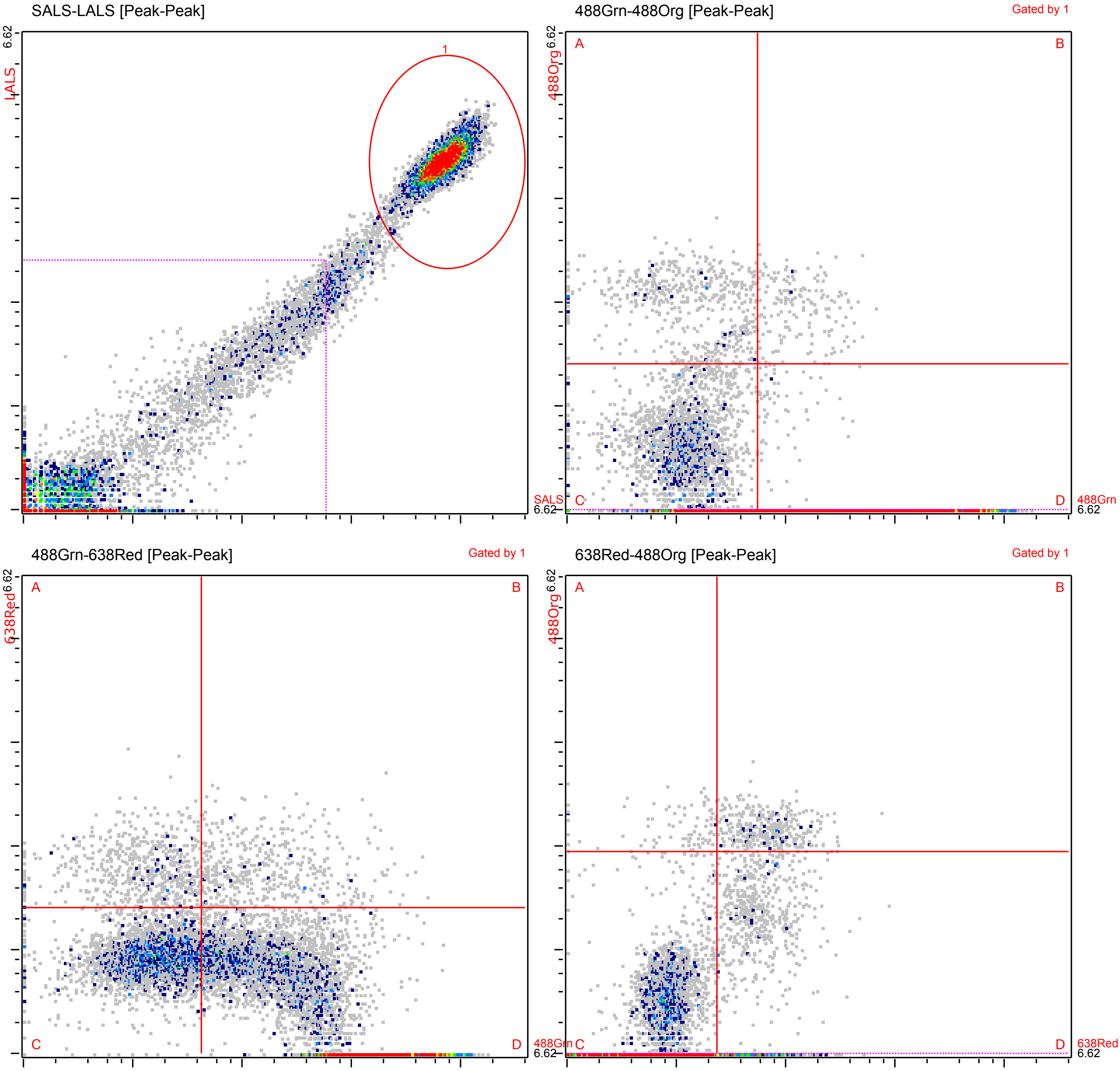

Apogee Flow Cytometry Report
Apogee Flow Cytometer

Acquisition Date:	17 May 2021 15:41:41
Filename:	Sample_210517_1361_0.fcs
Sample ID:	Sample_210517_1361
Operator:	A0149\ApogeeFlow
Protocol:	

Cytogram ROI Statistics

ROI ID	Events	Events/µl	%	Ratio	Mean X	Mean Y
1	10059	2514.8	16.8%		746238	246203
488Grn--A	550	137.5	5.5%			
488Grn--B	169	42.3	1.7%			
488Grn--C	3949	987.3	39.3%			
488Grn--D	5391	1347.8	53.6%			
488Grn--A	634	158.5	6.3%			
488Grn--B	471	117.8	4.7%			
488Grn--C	3440	860.0	34.2%			
488Grn--D	5514	1378.5	54.8%			
638Red--A	83	20.8	0.8%			
638Red--B	361	90.3	3.6%			
638Red--C	8835	2208.8	87.8%			
638Red--D	780	195.0	7.8%			

Acquisition Parameters

Channel	PMT	Gain	Thresh (OR)	Subtraction
SALS	330	1.00	954	
LALS	350	1.00	414	
488Grn	285	1.00		0.00%, 0.00%, 0.00%
488Org	340	1.00	1	28.00%, 0.00%, 0.00%
488Red	520	1.00		0.00%, 0.00%, 0.00%
488DpRed	500	1.00		0.00%, 0.00%, 0.00%

Instrument Settings

Pressure	Dilution	Sample Flow	Acquisition Time
75 counts	factor of 1	15.00 ul/min	16 secs

CYT: Universal

CYTSN: 0149

Apogee Flow Systems Limited

Apogee Flow Cytometry Report
Apogee Flow Cytometer

Acquisition Date:	17 May 2021 15:48:18
Filename:	Sample_210517_1364_0.fcs
Sample ID:	Sample_210517_1364
Operator:	A0149\ApogeeFlow
Protocol:	

SALS-LALS [Peak-Peak]

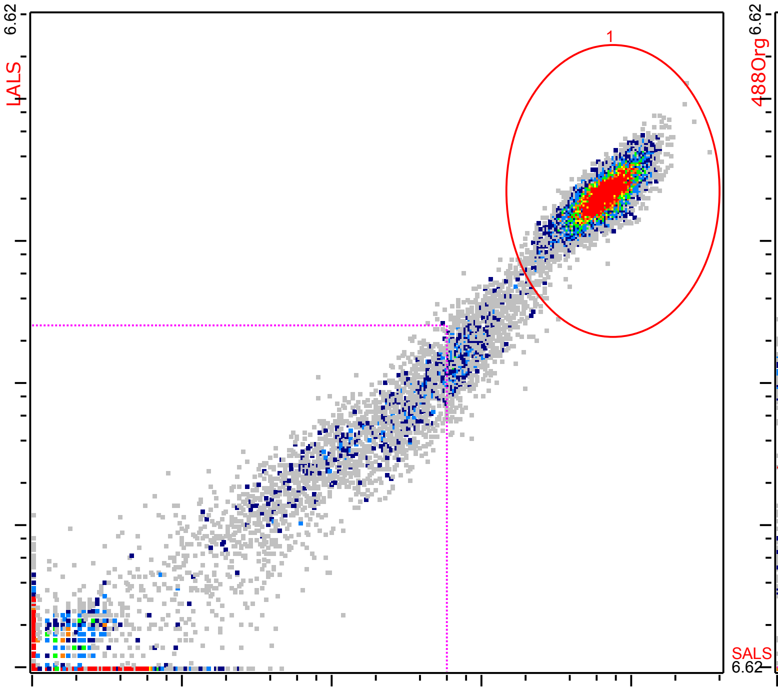

488Grn-488Org [Peak-Peak]

Gated by 1

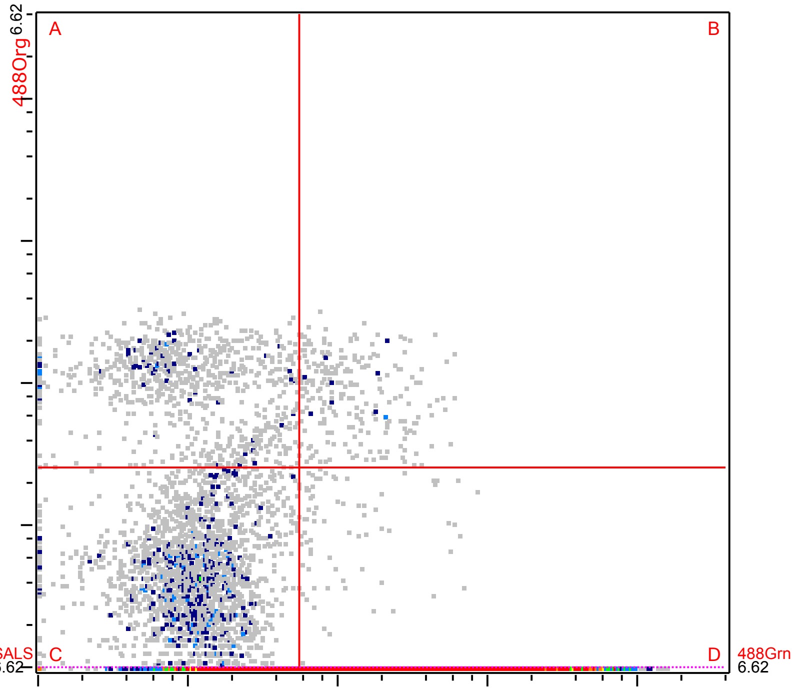

488Grn-638Red [Peak-Peak]

Gated by 1

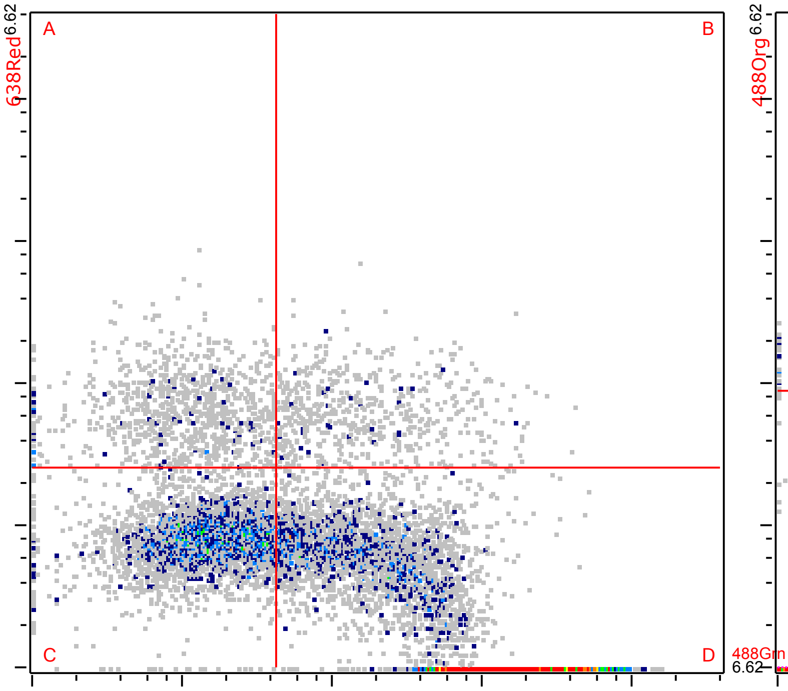

638Red-488Org [Peak-Peak]

Gated by 1

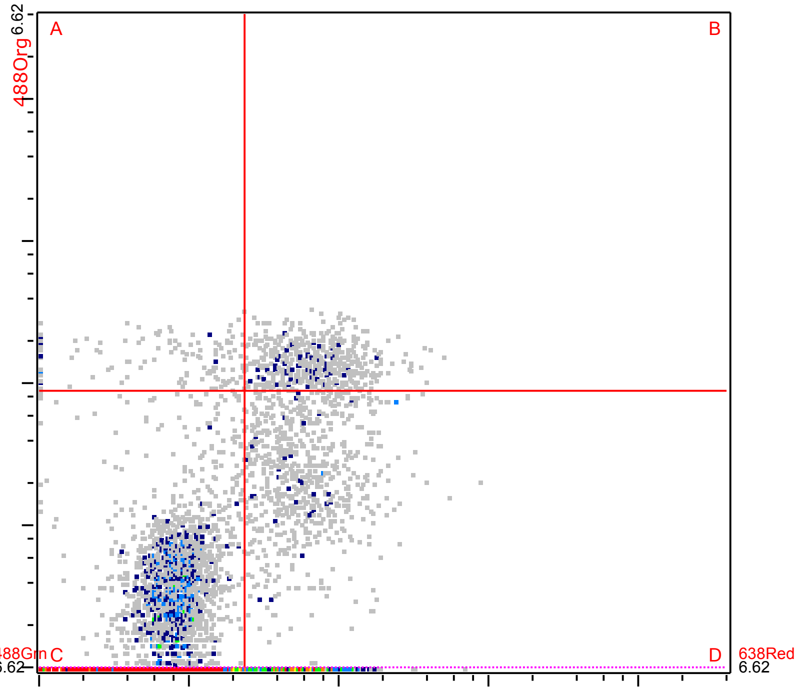

Apogee Flow Cytometry Report
Apogee Flow Cytometer

Acquisition Date:	17 May 2021 15:48:18
Filename:	Sample_210517_1364_0.fcs
Sample ID:	Sample_210517_1364
Operator:	A0149\ApogeeFlow
Protocol:	

Cytogram ROI Statistics

ROI ID	Events	Events/µl	%	Ratio	Mean X	Mean Y
1	10030	2111.6	43.6%		708214	223559
488Grn--A	844	177.7	8.4%			
488Grn--B	229	48.2	2.3%			
488Grn--C	4126	868.6	41.1%			
488Grn--D	4831	1017.1	48.2%			
488Grn--A	940	197.9	9.4%			
488Grn--B	607	127.8	6.1%			
488Grn--C	3600	757.9	35.9%			
488Grn--D	4883	1028.0	48.7%			
638Red--A	129	27.2	1.3%			
638Red--B	543	114.3	5.4%			
638Red--C	8319	1751.4	82.9%			
638Red--D	1039	218.7	10.4%			

Acquisition Parameters

Channel	PMT	Gain	Thresh (OR)	Subtraction
SALS	330	1.00	954	
LALS	350	1.00	414	
488Grn	285	1.00		0.00%, 0.00%, 0.00%
488Org	340	1.00	1	28.00%, 0.00%, 0.00%
488Red	520	1.00		0.00%, 0.00%, 0.00%
488DpRed	500	1.00		0.00%, 0.00%, 0.00%

Instrument Settings

Pressure	Dilution	Sample Flow	Acquisition Time
75 counts	factor of 1	15.00 ul/min	19 secs

CYT: Universal

CYTSN: 0149

Apogee Flow Systems Limited

Apogee Flow Cytometry Report
Apogee Flow Cytometer

Acquisition Date:	17 May 2021 15:14:07
Filename:	Sample_210517_1349_0.fcs
Sample ID:	Sample_210517_1349
Operator:	A0149\ApogeeFlow
Protocol:	

SALS-LALS [Peak-Peak]

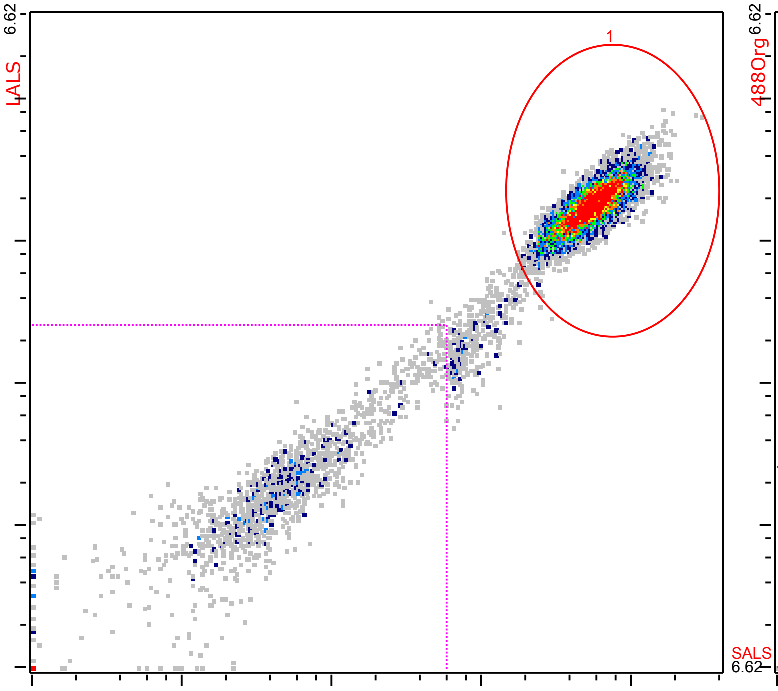

488Grn-488Org [Peak-Peak]

Gated by 1

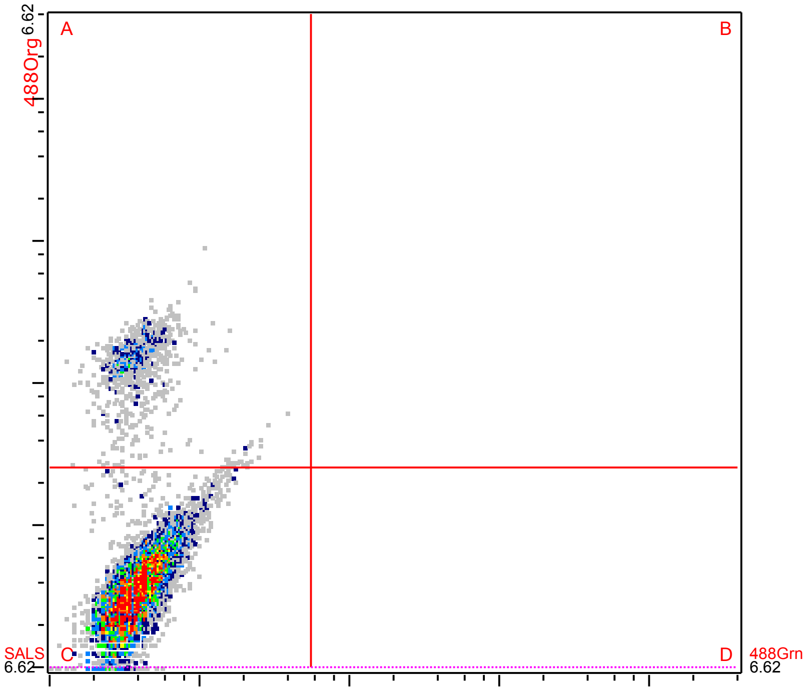

488Grn-638Red [Peak-Peak]

Gated by 1

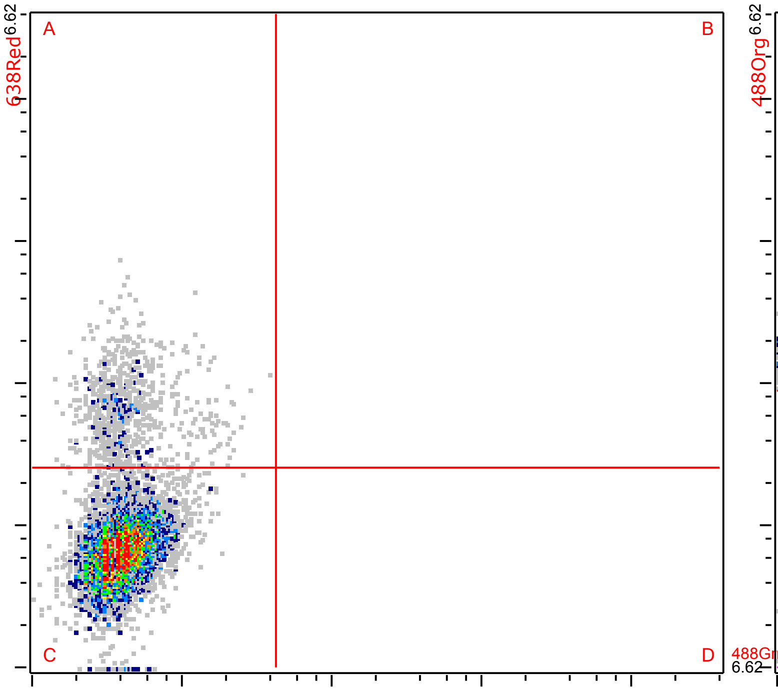

638Red-488Org [Peak-Peak]

Gated by 1

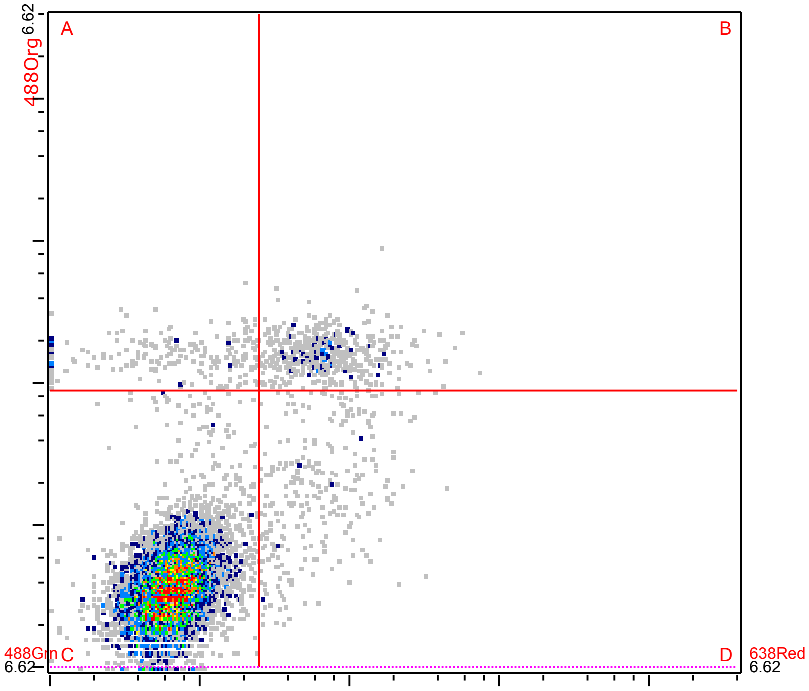

Apogee Flow Cytometry Report
Apogee Flow Cytometer

Acquisition Date:	17 May 2021 15:14:07
Filename:	Sample_210517_1349_0.fcs
Sample ID:	Sample_210517_1349
Operator:	A0149\ApogeeFlow
Protocol:	

Cytogram ROI Statistics

ROI ID	Events	Events/µl	%	Ratio	Mean X	Mean Y
1	10099	3107.4	84.5%		590164	186751
488Grn--A	869	267.4	8.6%			
488Grn--B	0	0.0	0.0%			
488Grn--C	9230	2840.0	91.4%			
488Grn--D	0	0.0	0.0%			
488Grn--A	774	238.2	7.7%			
488Grn--B	0	0.0	0.0%			
488Grn--C	9325	2869.2	92.3%			
488Grn--D	0	0.0	0.0%			
638Red--A	210	64.6	2.1%			
638Red--B	504	155.1	5.0%			
638Red--C	9115	2804.6	90.3%			
638Red--D	270	83.1	2.7%			

Acquisition Parameters

Channel	PMT	Gain	Thresh (OR)	Subtraction
SALS	330	1.00	954	
LALS	350	1.00	414	
488Grn	285	1.00		0.00%, 0.00%, 0.00%
488Org	340	1.00	1	28.00%, 0.00%, 0.00%
488Red	520	1.00		0.00%, 0.00%, 0.00%
488DpRed	500	1.00		0.00%, 0.00%, 0.00%

Instrument Settings

Pressure	Dilution	Sample Flow	Acquisition Time
75 counts	factor of 1	15.00 ul/min	13 secs

Apogee Flow Cytometry Report
Apogee Flow Cytometer

Acquisition Date:	17 May 2021 15:16:22
Filename:	Sample_210517_1350_0.fcs
Sample ID:	Sample_210517_1350
Operator:	A0149\ApogeeFlow
Protocol:	

SALS-LALS [Peak-Peak]

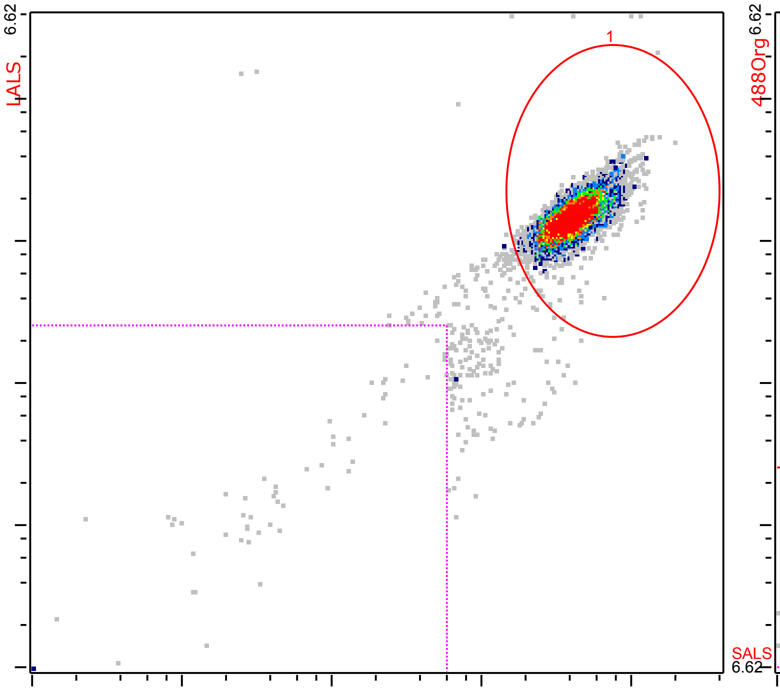

488Grn-488Org [Peak-Peak]

Gated by 1

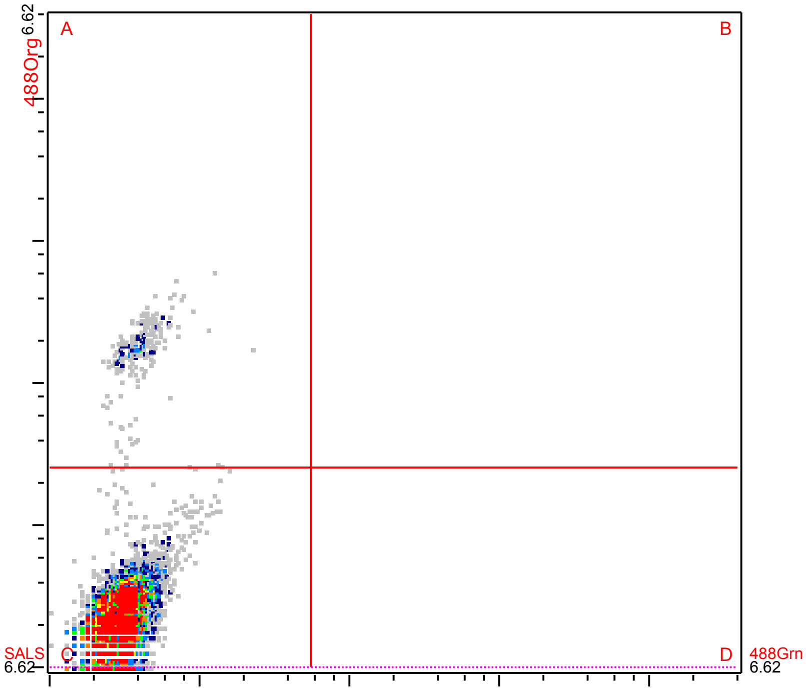

488Grn-638Red [Peak-Peak]

Gated by 1

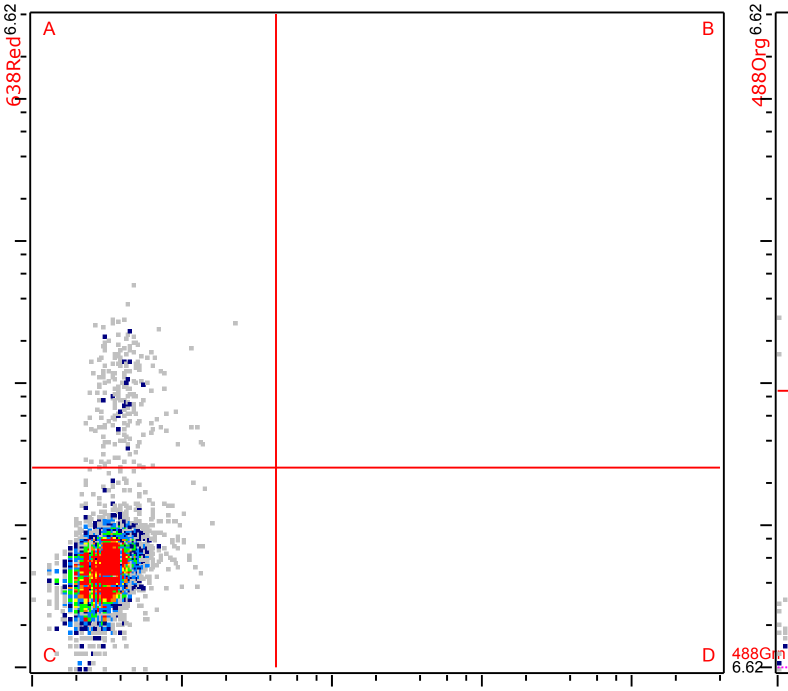

638Red-488Org [Peak-Peak]

Gated by 1

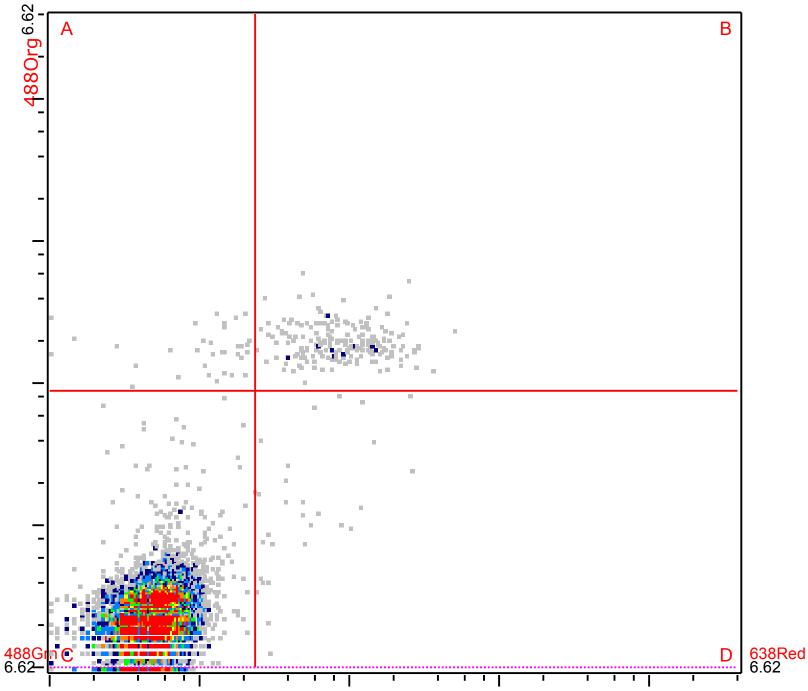

Apogee Flow Cytometry Report
Apogee Flow Cytometer

Acquisition Date:	17 May 2021 15:16:22
Filename:	Sample_210517_1350_0.fcs
Sample ID:	Sample_210517_1350
Operator:	A0149\ApogeeFlow
Protocol:	

Cytogram ROI Statistics

ROI ID	Events	Events/µl	%	Ratio	Mean X	Mean Y
1	10360	5920.0	97.2%		433260	153265
488Grn--A	261	149.1	2.5%			
488Grn--B	0	0.0	0.0%			
488Grn--C	10099	5770.9	97.5%			
488Grn--D	0	0.0	0.0%			
488Grn--A	229	130.9	2.2%			
488Grn--B	0	0.0	0.0%			
488Grn--C	10131	5789.1	97.8%			
488Grn--D	0	0.0	0.0%			
638Red--A	33	18.9	0.3%			
638Red--B	204	116.6	2.0%			
638Red--C	10095	5768.6	97.4%			
638Red--D	28	16.0	0.3%			

Acquisition Parameters

Channel	PMT	Gain	Thresh (OR)	Subtraction
SALS	330	1.00	954	
LALS	350	1.00	414	
488Grn	285	1.00		0.00%, 0.00%, 0.00%
488Org	340	1.00	1	28.00%, 0.00%, 0.00%
488Red	520	1.00		0.00%, 0.00%, 0.00%
488DpRed	500	1.00		0.00%, 0.00%, 0.00%

Instrument Settings

Pressure	Dilution	Sample Flow	Acquisition Time
75 counts	factor of 1	15.00 ul/min	7 secs

CYT: Universal
CYTSN: 0149
Apogee Flow Systems Limited

APOGEE FLOW SYSTEMS

Apogee Flow Cytometry Report
Apogee Flow Cytometer

Acquisition Date:	19 May 2021 16:27:57
Filename:	Sample_210519_1412_0.fcs
Sample ID:	Sample_210519_1412
Operator:	A0149\ApogeeFlow
Protocol:	

SALS-LALS [Peak-Peak]

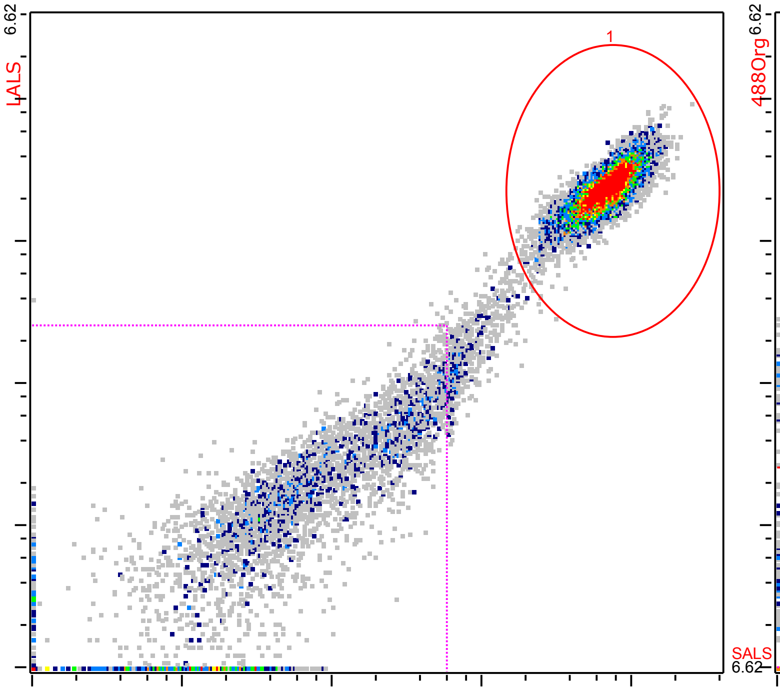

488Grn-488Org [Peak-Peak]

Gated by 1

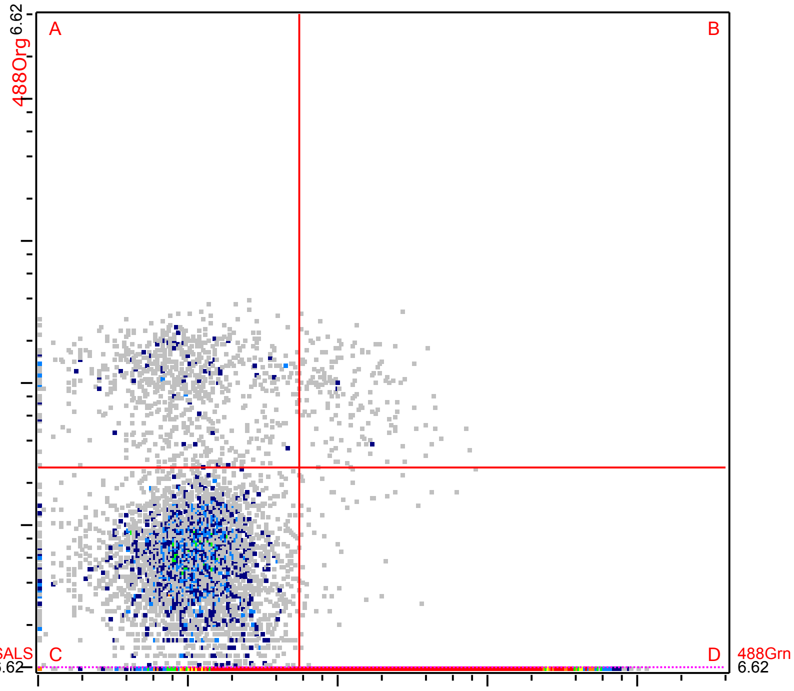

488Grn-638Red [Peak-Peak]

Gated by 1

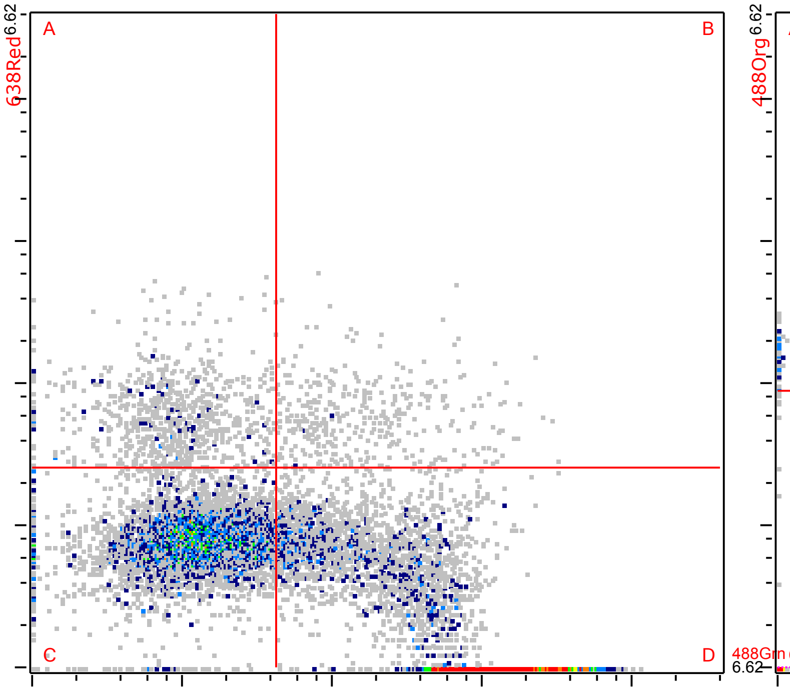

638Red-488Org [Peak-Peak]

Gated by 1

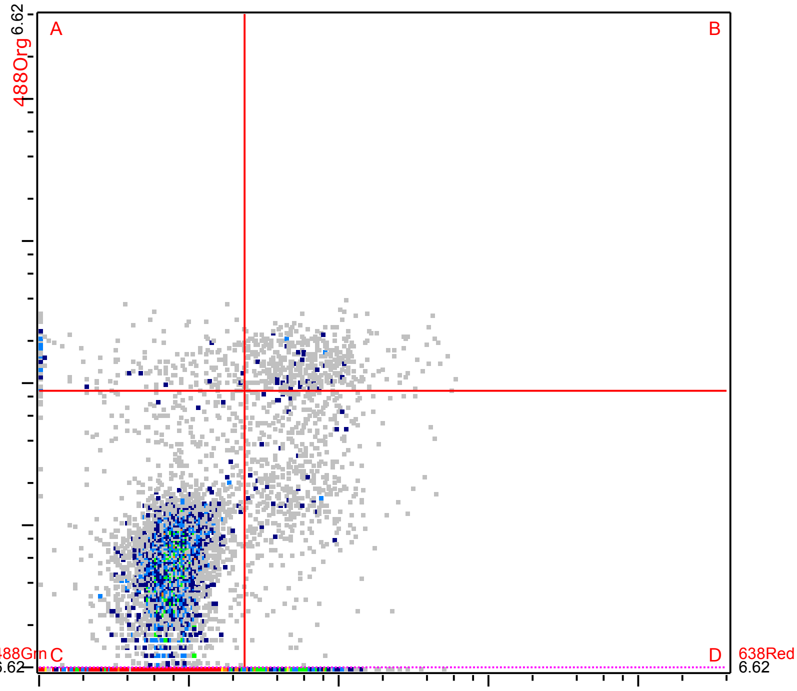

Apogee Flow Cytometry Report
Apogee Flow Cytometer

Acquisition Date:	19 May 2021 16:27:57
Filename:	Sample_210519_1412_0.fcs
Sample ID:	Sample_210519_1412
Operator:	A0149\ApogeeFlow
Protocol:	

Cytogram ROI Statistics

ROI ID	Events	Events/µl	%	Ratio	Mean X	Mean Y
1	10494	2798.4	44.3%		726836	246655
488Grn--A	866	230.9	8.3%			
488Grn--B	167	44.5	1.6%			
488Grn--C	5591	1490.9	53.3%			
488Grn--D	3870	1032.0	36.9%			
488Grn--A	817	217.9	7.8%			
488Grn--B	361	96.3	3.4%			
488Grn--C	5281	1408.3	50.3%			
488Grn--D	4035	1076.0	38.5%			
638Red--A	206	54.9	2.0%			
638Red--B	450	120.0	4.3%			
638Red--C	9061	2416.3	86.3%			
638Red--D	777	207.2	7.4%			

Acquisition Parameters

Channel	PMT	Gain	Thresh (OR)	Subtraction
SALS	330	1.00	954	
LALS	350	1.00	414	
488Grn	285	1.00		0.00%, 0.00%, 0.00%
488Org	340	1.00	1	28.00%, 0.00%, 0.00%
488Red	520	1.00		0.00%, 0.00%, 0.00%
488DpRed	500	1.00		0.00%, 0.00%, 0.00%

Instrument Settings

Pressure	Dilution	Sample Flow	Acquisition Time
75 counts	factor of 1	15.00 ul/min	15 secs

CYT: Universal
CYTSN: 0149
Apogee Flow Systems Limited

APOGEE FLOW SYSTEMS

Apogee Flow Cytometry Report
Apogee Flow Cytometer

Acquisition Date:	16 April 2021 15:54:27
Filename:	Sample_210416_1209_0.fcs
Sample ID:	Sample_210416_1209
Operator:	A0149\ApogeeFlow
Protocol:	

SALS-LALS [Peak-Peak]

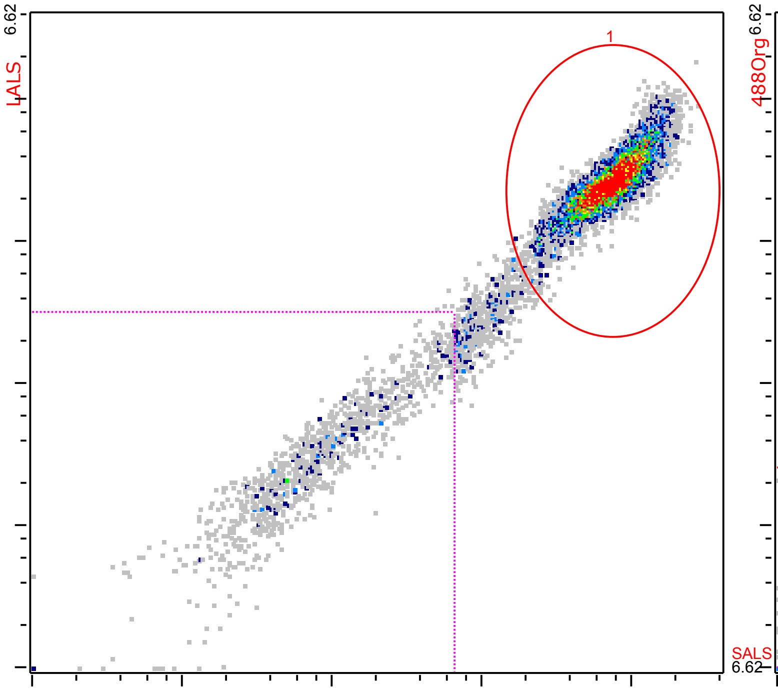

488Grn-488Org [Peak-Peak]

Gated by 1

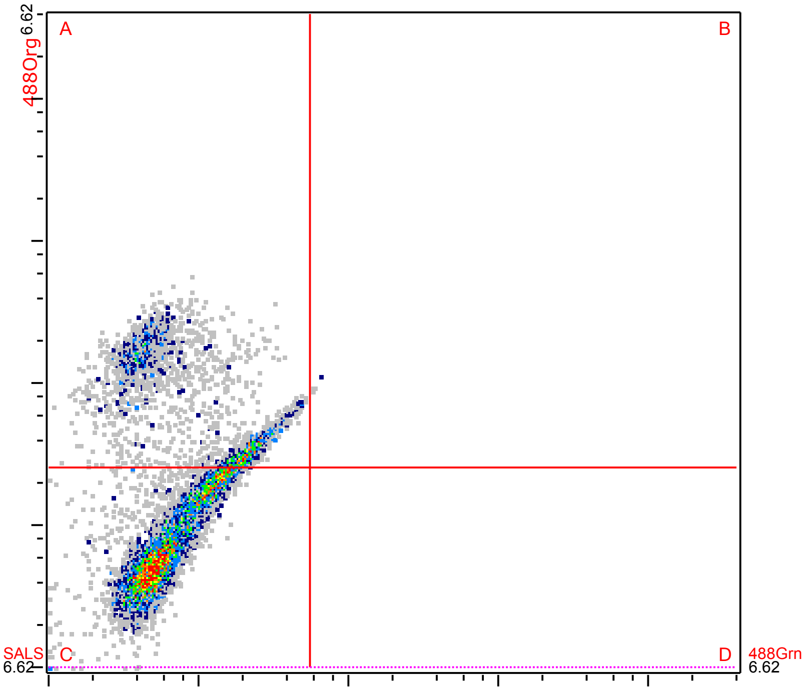

488Grn-638Red [Peak-Peak]

Gated by 1

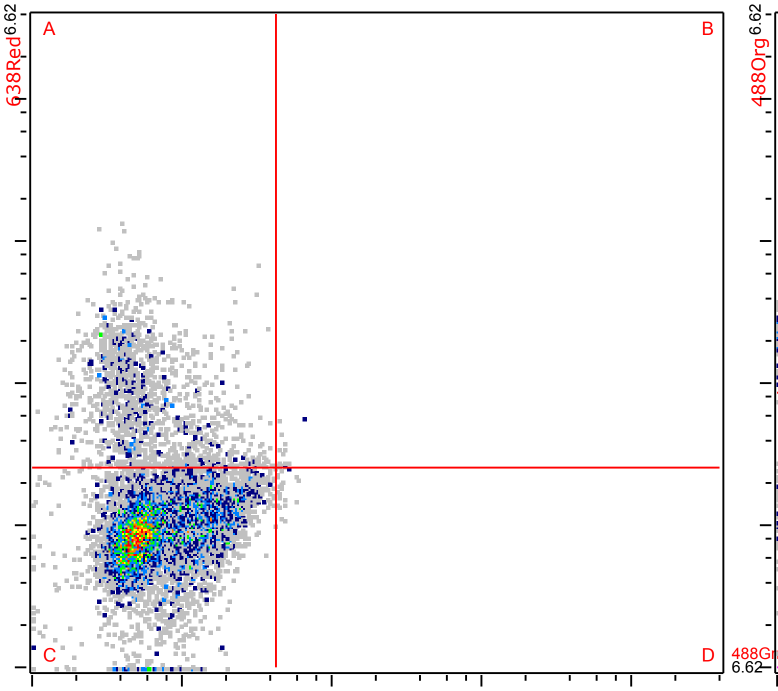

638Red-488Org [Peak-Peak]

Gated by 1

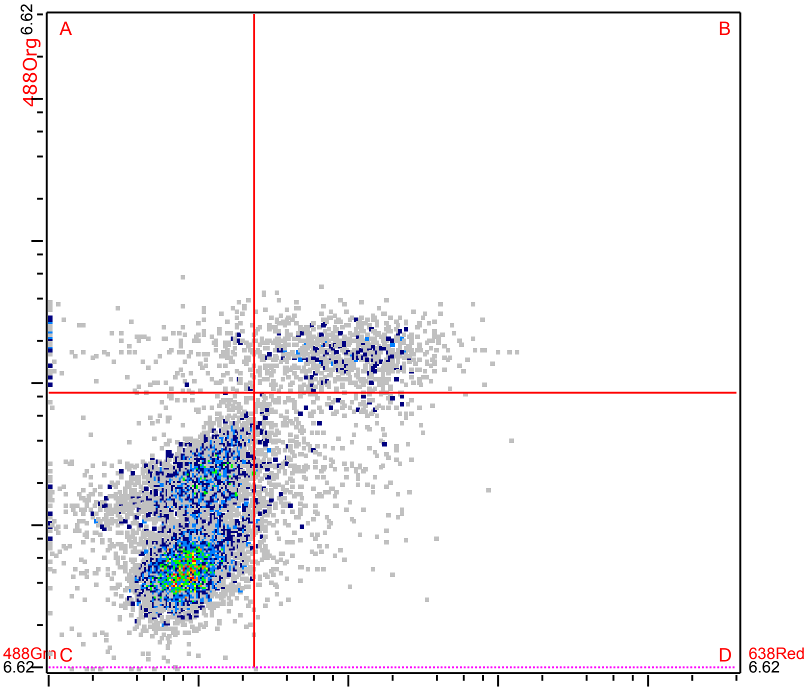

Apogee Flow Cytometry Report
Apogee Flow Cytometer

Acquisition Date:	16 April 2021 15:54:27
Filename:	Sample_210416_1209_0.fcs
Sample ID:	Sample_210416_1209
Operator:	A0149\ApogeeFlow
Protocol:	

Cytogram ROI Statistics

ROI ID	Events	Events/µl	%	Ratio	Mean X	Mean Y
1	10028	1161.1	85.6%		813064	289347
488Grn--A	2611	302.3	26.0%			
488Grn--B	5	0.6	0.0%			
488Grn--C	7412	858.2	73.9%			
488Grn--D	0	0.0	0.0%			
488Grn--A	1513	175.2	15.1%			
488Grn--B	17	2.0	0.2%			
488Grn--C	8477	981.5	84.5%			
488Grn--D	21	2.4	0.2%			
638Red--A	239	27.7	2.4%			
638Red--B	941	109.0	9.4%			
638Red--C	8181	947.3	81.6%			
638Red--D	667	77.2	6.7%			

Acquisition Parameters

Channel	PMT	Gain	Thresh (OR)	Subtraction
SALS	330	1.00	1075	
LALS	350	1.00	510	
488Grn	285	1.00		0.00%, 0.00%, 0.00%
488Org	340	1.00	1	28.00%, 0.00%, 0.00%
488Red	520	1.00		0.00%, 0.00%, 0.00%
488DpRed	500	1.00		0.00%, 0.00%, 0.00%

Instrument Settings

Pressure	Dilution	Sample Flow	Acquisition Time
75 counts	factor of 1	7.51 ul/min	69 secs

CYT: Universal
CYTSN: 0149
Apogee Flow Systems Limited

APOGEE FLOW SYSTEMS

Apogee Flow Cytometry Report
Apogee Flow Cytometer

Acquisition Date: 17 May 2021 15:34:01
Filename: Sample_210517_1358_0.fcs
Sample ID: Sample_210517_1358
Operator: A0149\ApogeeFlow
Protocol:

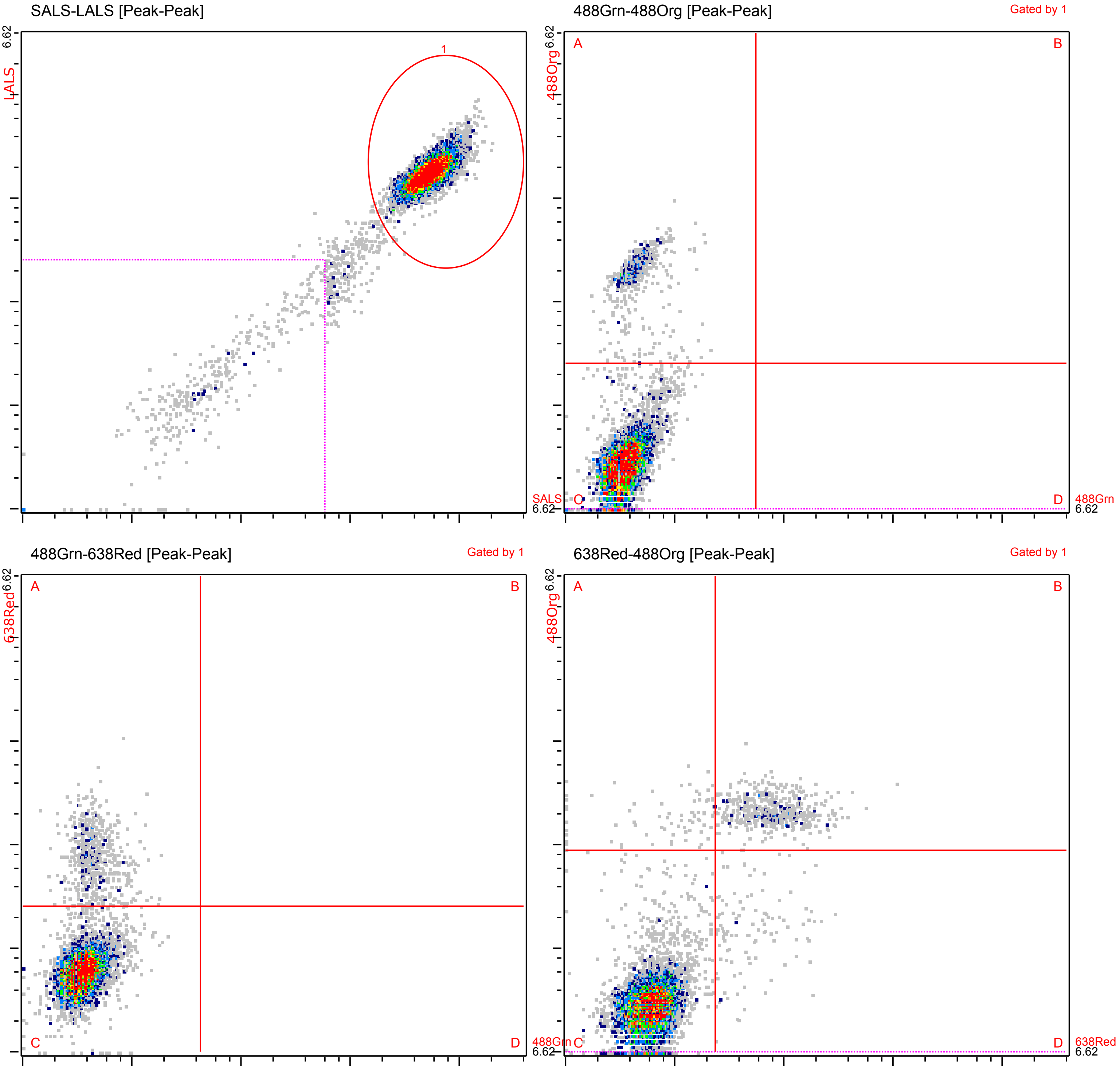

Apogee Flow Cytometry Report
Apogee Flow Cytometer

Acquisition Date:	17 May 2021 15:34:01
Filename:	Sample_210517_1358_0.fcs
Sample ID:	Sample_210517_1358
Operator:	A0149\ApogeeFlow
Protocol:	

Cytogram ROI Statistics

ROI ID	Events	Events/µl	%	Ratio	Mean X	Mean Y
1	10015	2356.5	94.1%		560590	183528
488Grn--A	671	157.9	6.7%			
488Grn--B	0	0.0	0.0%			
488Grn--C	9344	2198.6	93.3%			
488Grn--D	0	0.0	0.0%			
488Grn--A	617	145.2	6.2%			
488Grn--B	0	0.0	0.0%			
488Grn--C	9398	2211.3	93.8%			
488Grn--D	0	0.0	0.0%			
638Red--A	78	18.4	0.8%			
638Red--B	521	122.6	5.2%			
638Red--C	9306	2189.6	92.9%			
638Red--D	110	25.9	1.1%			

Acquisition Parameters

Channel	PMT	Gain	Thresh (OR)	Subtraction
SALS	330	1.00	954	
LALS	350	1.00	414	
488Grn	285	1.00		0.00%, 0.00%, 0.00%
488Org	340	1.00	1	28.00%, 0.00%, 0.00%
488Red	520	1.00		0.00%, 0.00%, 0.00%
488DpRed	500	1.00		0.00%, 0.00%, 0.00%

Instrument Settings

Pressure	Dilution	Sample Flow	Acquisition Time
75 counts	factor of 1	15.00 ul/min	17 secs

CYT: Universal

CYTSN: 0149

Apogee Flow Systems Limited

APOGEE FLOW SYSTEMS

Apogee Flow Cytometry Report
Apogee Flow Cytometer

Acquisition Date:	18 May 2021 15:41:35
Filename:	Sample_210518_1389_0.fcs
Sample ID:	Sample_210518_1389
Operator:	A0149\ApogeeFlow
Protocol:	

SALS-LALS [Peak-Peak]

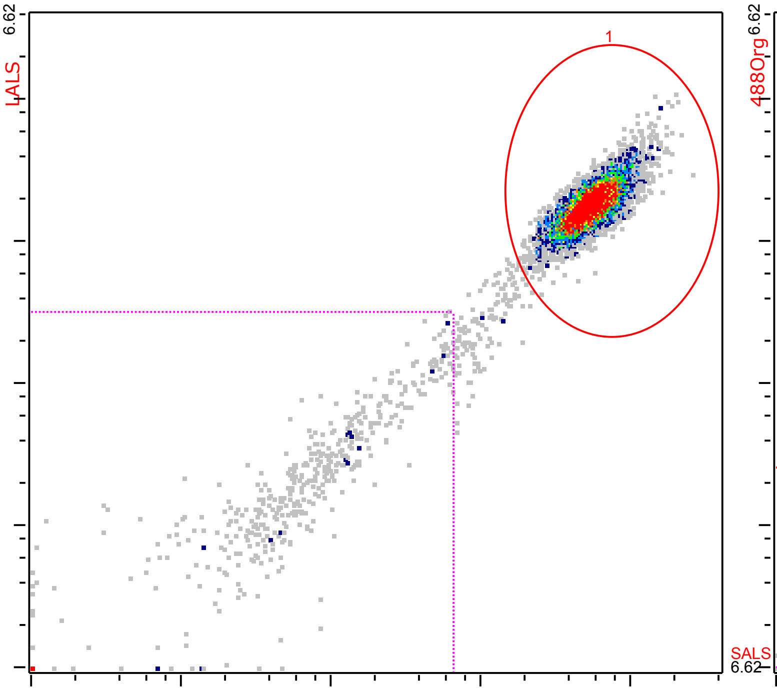

488Grn-488Org [Peak-Peak]

Gated by 1

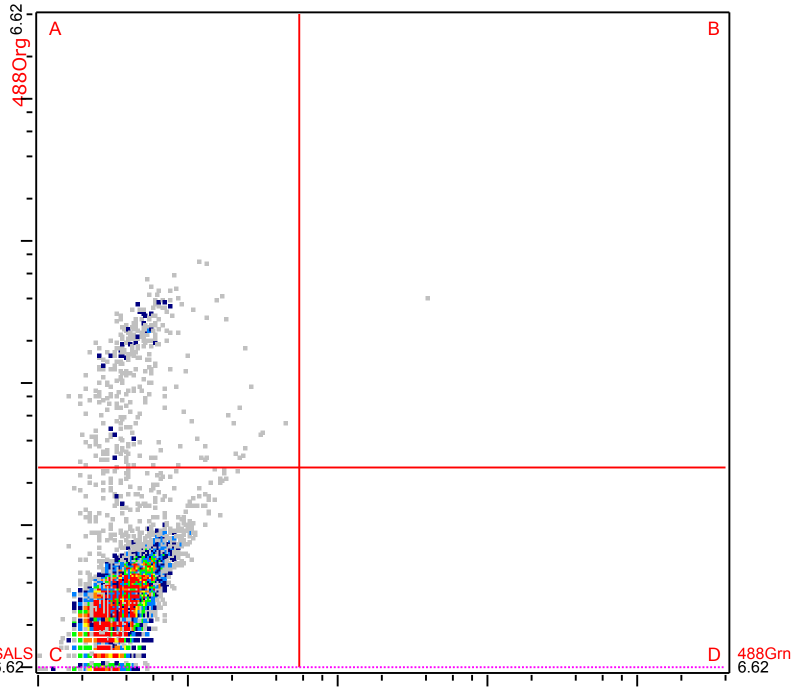

488Grn-638Red [Peak-Peak]

Gated by 1

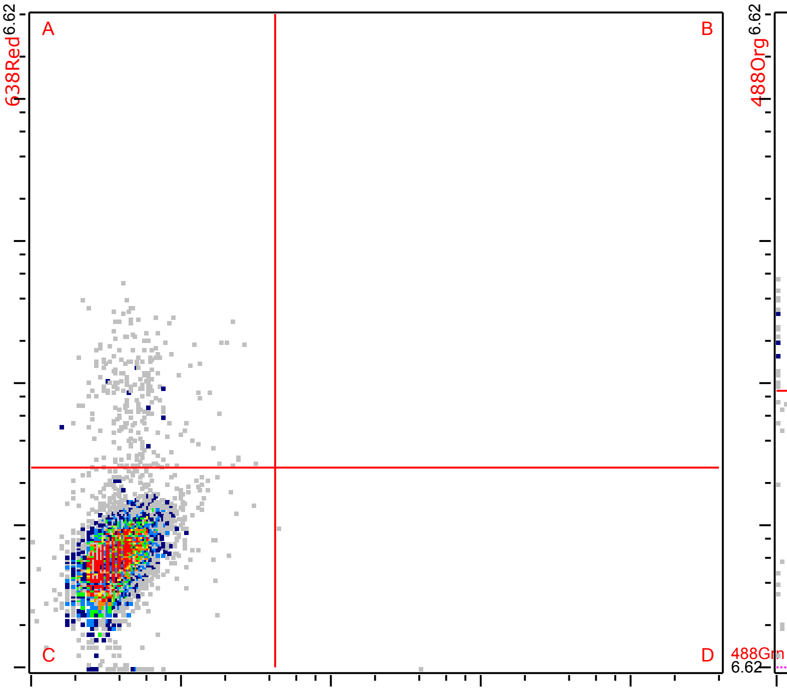

638Red-488Org [Peak-Peak]

Gated by 1

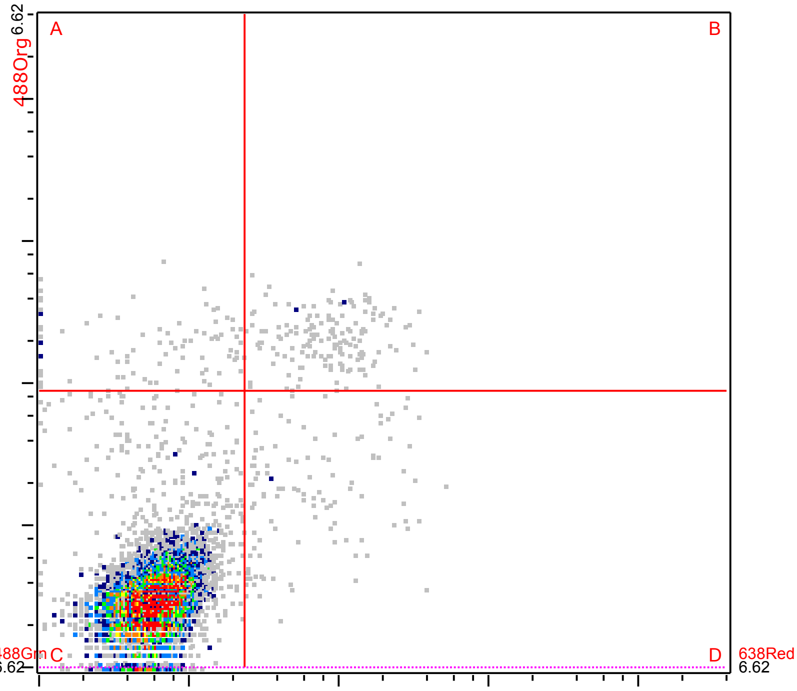

Apogee Flow Cytometry Report
Apogee Flow Cytometer

Acquisition Date:	18 May 2021 15:41:35
Filename:	Sample_210518_1389_0.fcs
Sample ID:	Sample_210518_1389
Operator:	A0149\ApogeeFlow
Protocol:	

Cytogram ROI Statistics

ROI ID	Events	Events/µl	%	Ratio	Mean X	Mean Y
1	10118	4755.1	94.6%		580440	188886
488Grn--A	359	168.7	3.5%			
488Grn--B	1	0.5	0.0%			
488Grn--C	9758	4585.9	96.4%			
488Grn--D	0	0.0	0.0%			
488Grn--A	259	121.7	2.6%			
488Grn--B	0	0.0	0.0%			
488Grn--C	9857	4632.4	97.4%			
488Grn--D	2	0.9	0.0%			
638Red--A	84	39.5	0.8%			
638Red--B	157	73.8	1.6%			
638Red--C	9768	4590.6	96.5%			
638Red--D	109	51.2	1.1%			

Acquisition Parameters

Channel	PMT	Gain	Thresh (OR)	Subtraction
SALS	330	1.00	1075	
LALS	350	1.00	510	
488Grn	285	1.00		0.00%, 0.00%, 0.00%
488Org	340	1.00	1	28.00%, 0.00%, 0.00%
488Red	520	1.00		0.00%, 0.00%, 0.00%
488DpRed	500	1.00		0.00%, 0.00%, 0.00%

Instrument Settings

Pressure	Dilution	Sample Flow	Acquisition Time
75 counts	factor of 1	7.51 ul/min	17 secs

CYT: Universal

CYTSN: 0149

Apogee Flow Systems Limited

APOGEE FLOW SYSTEMS
