# Supplementary material for: Circular RNA CDR1as Alleviates Cisplatin-Based Chemoresistance by Suppressing MiR-1299 in Ovarian Cancer
Source: Front Genet. 2022 Jan 26;12:815448. doi: 10.3389/fgene.2021.815448 (PMC8826532; doi:10.3389/fgene.2021.815448)
Supplement: Supplementary file 7 [file DataSheet1.PDF]

Supplementary Material

1.1 Supplementary Figure

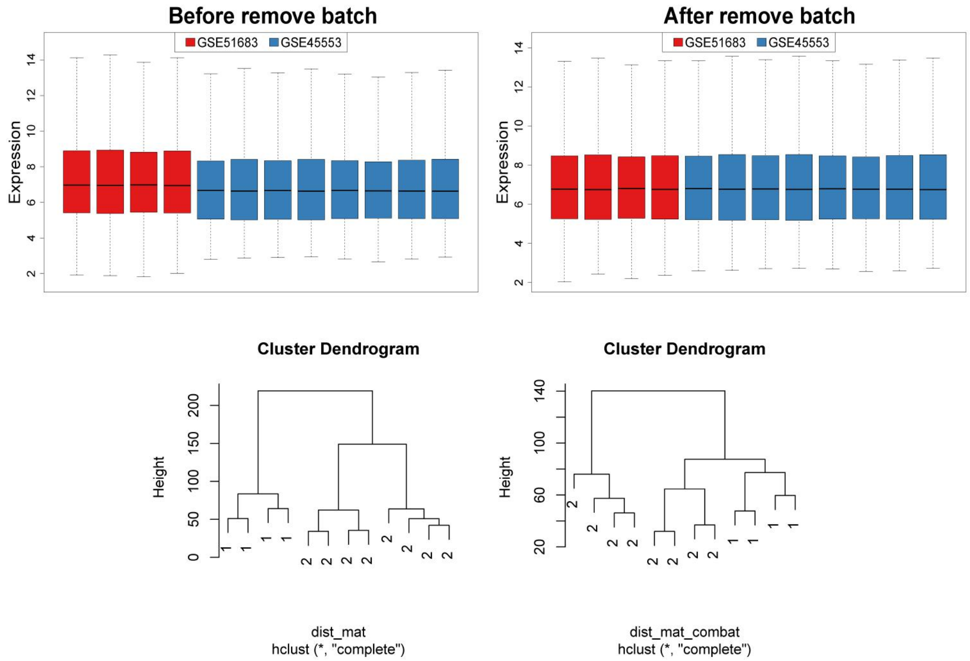

Supplementary Figure 1. Re-annotated probes and detected the expression of CDR1as in two microarray datasets.Cluster Dendrogram demonstrated the data was comparable.
