# Supplementary material for: Circular RNA CDR1as Alleviates Cisplatin-Based Chemoresistance by Suppressing MiR-1299 in Ovarian Cancer
Source: Front Genet. 2022 Jan 26;12:815448. doi: 10.3389/fgene.2021.815448 (PMC8826532; doi:10.3389/fgene.2021.815448)

4A

0-HO8910/DDP-CONTROL

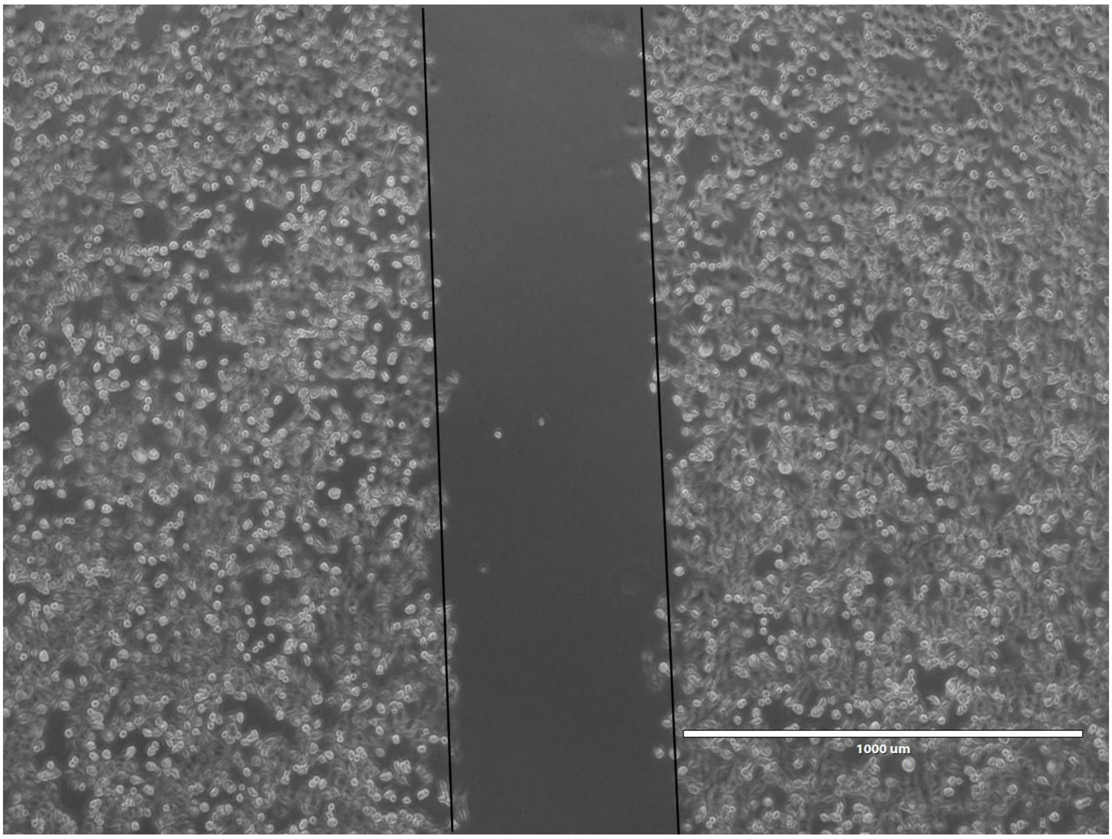

0-HO8910/DDP-h

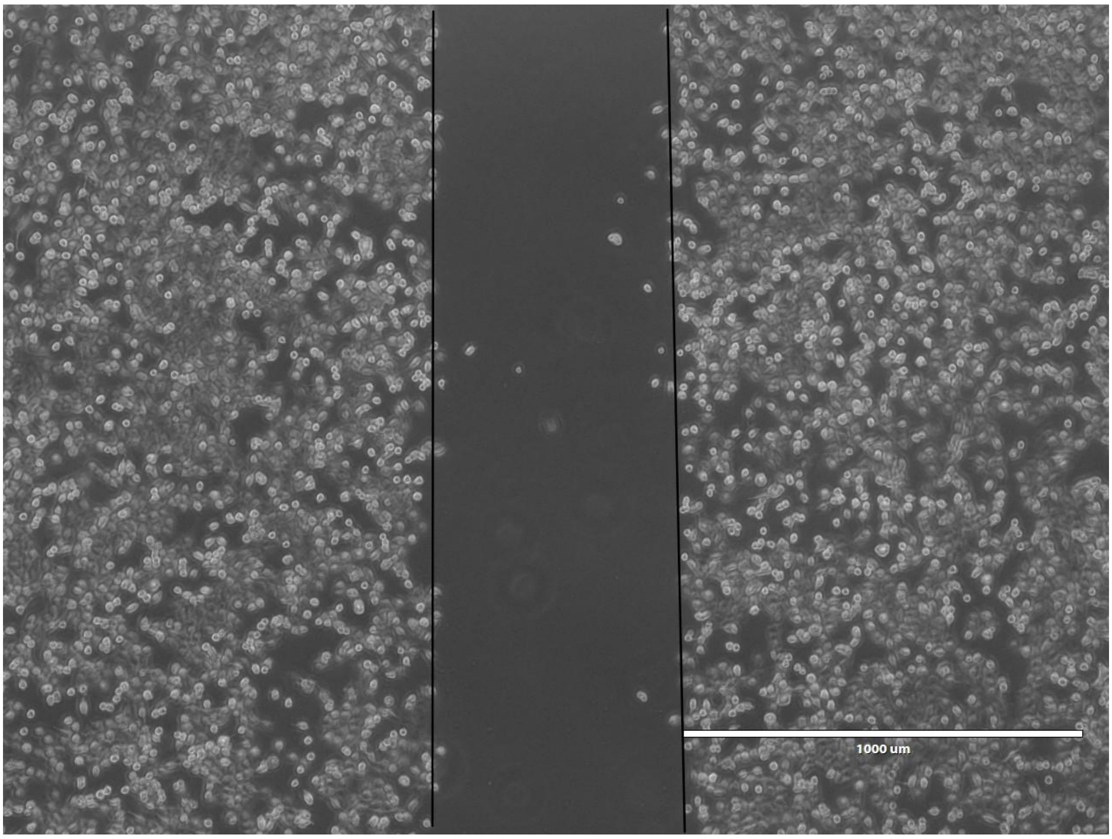

0-skov3cddp-control

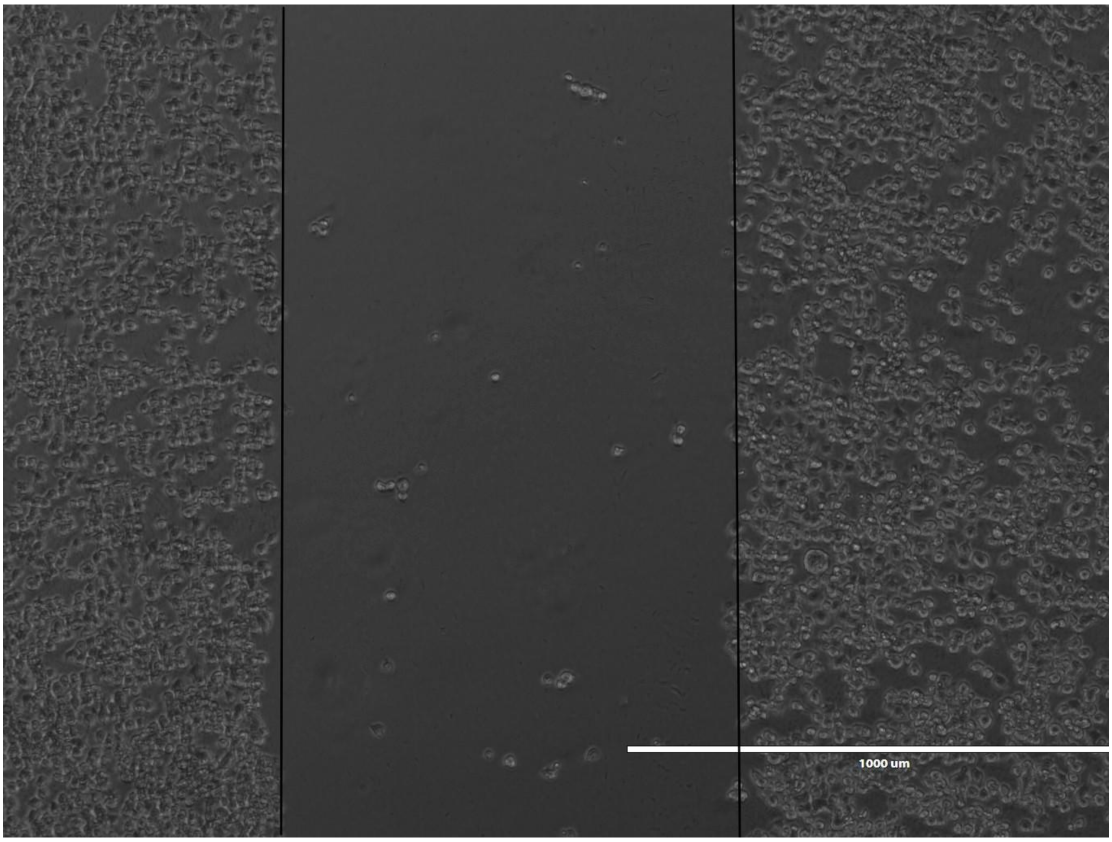

0-skov3/ddp-h

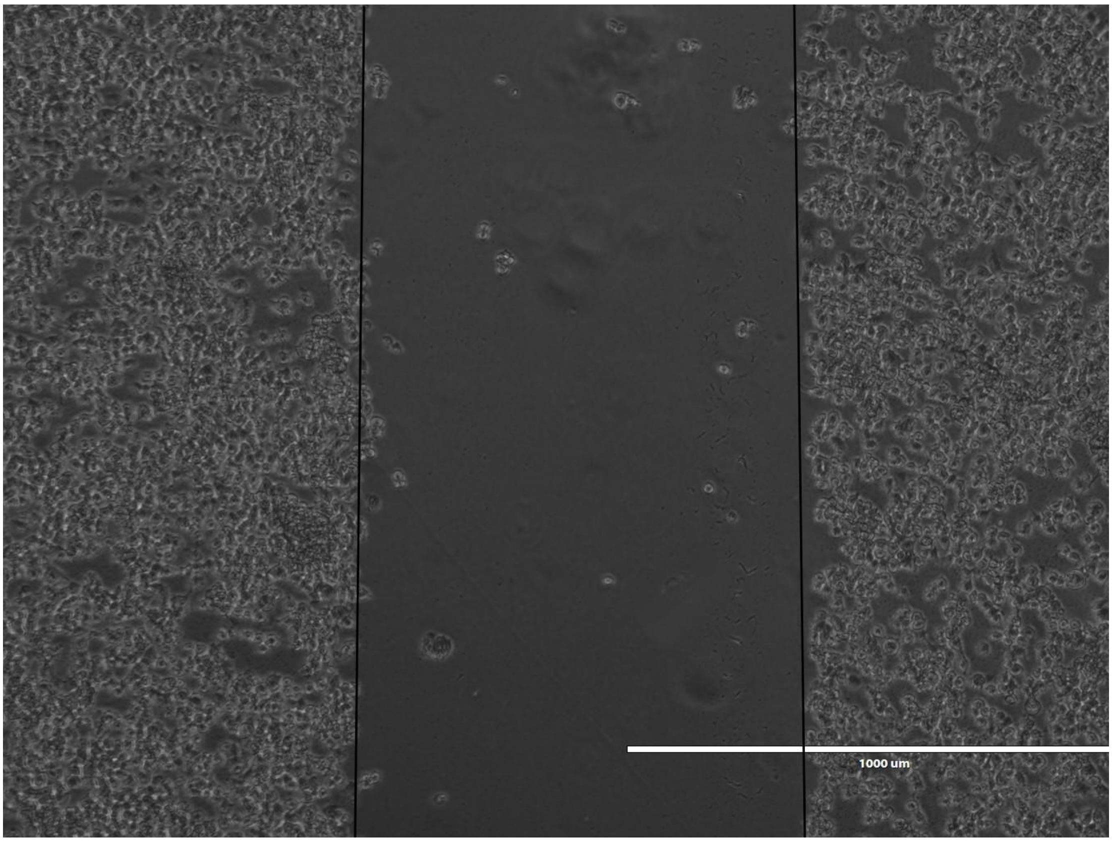

24-skov3/ddp-h

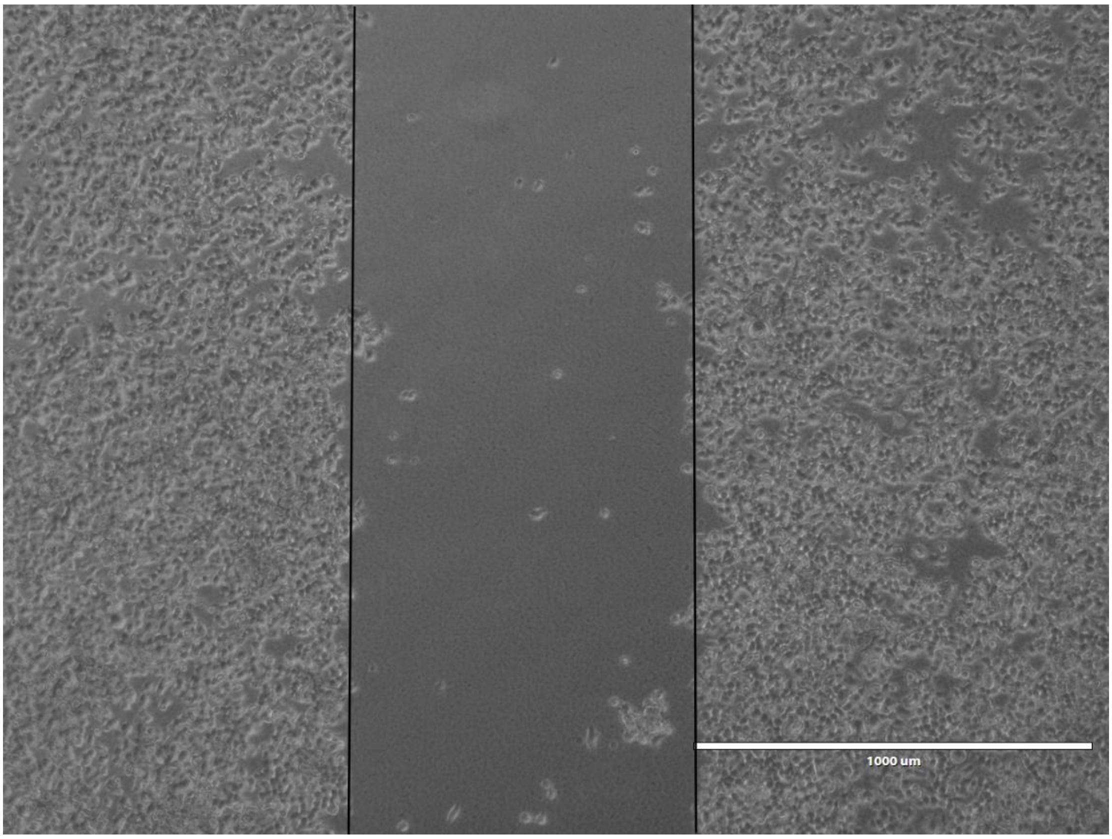

24 skov3ddp-control

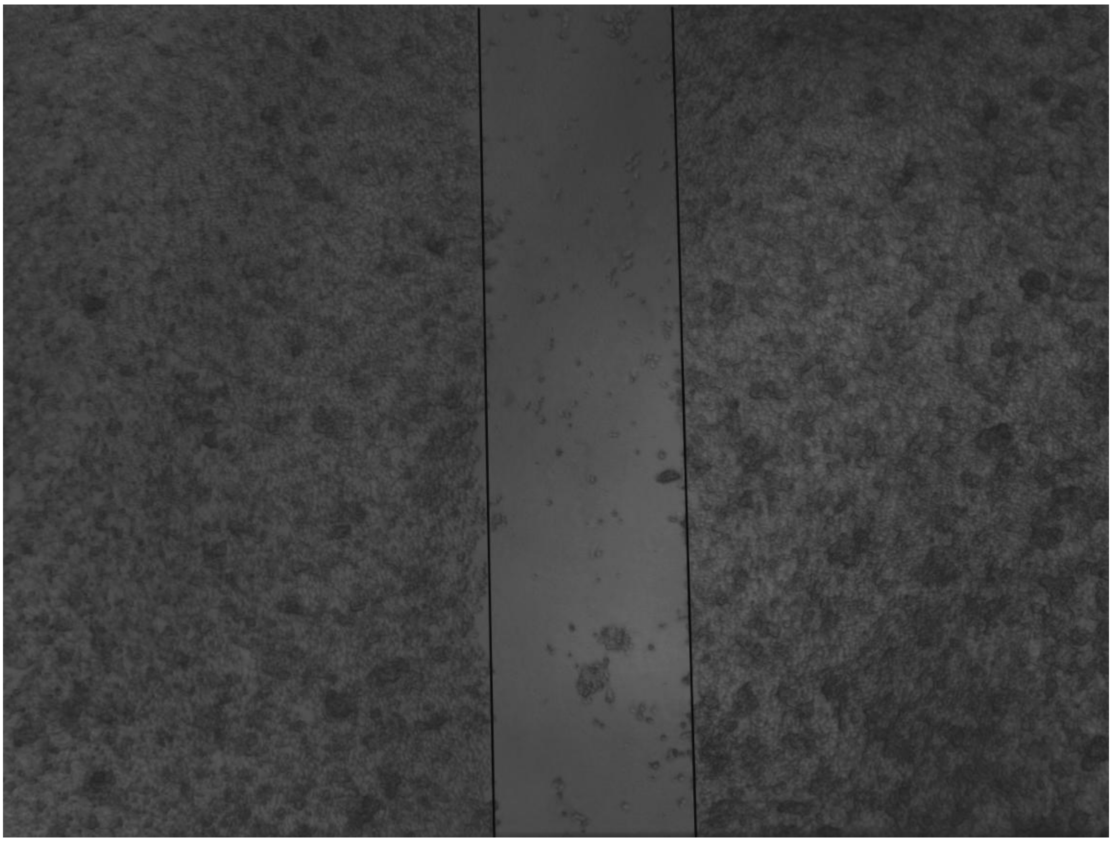

24-8910ddp-h

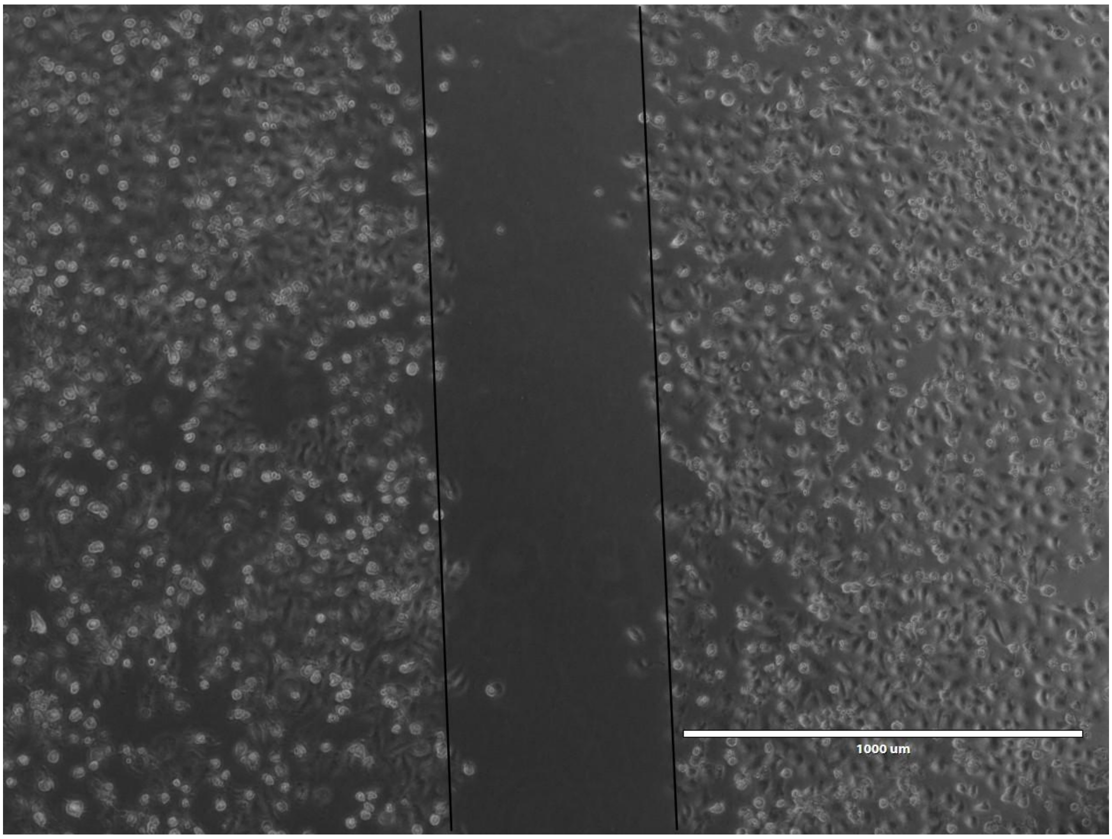

24-8910ddp-control

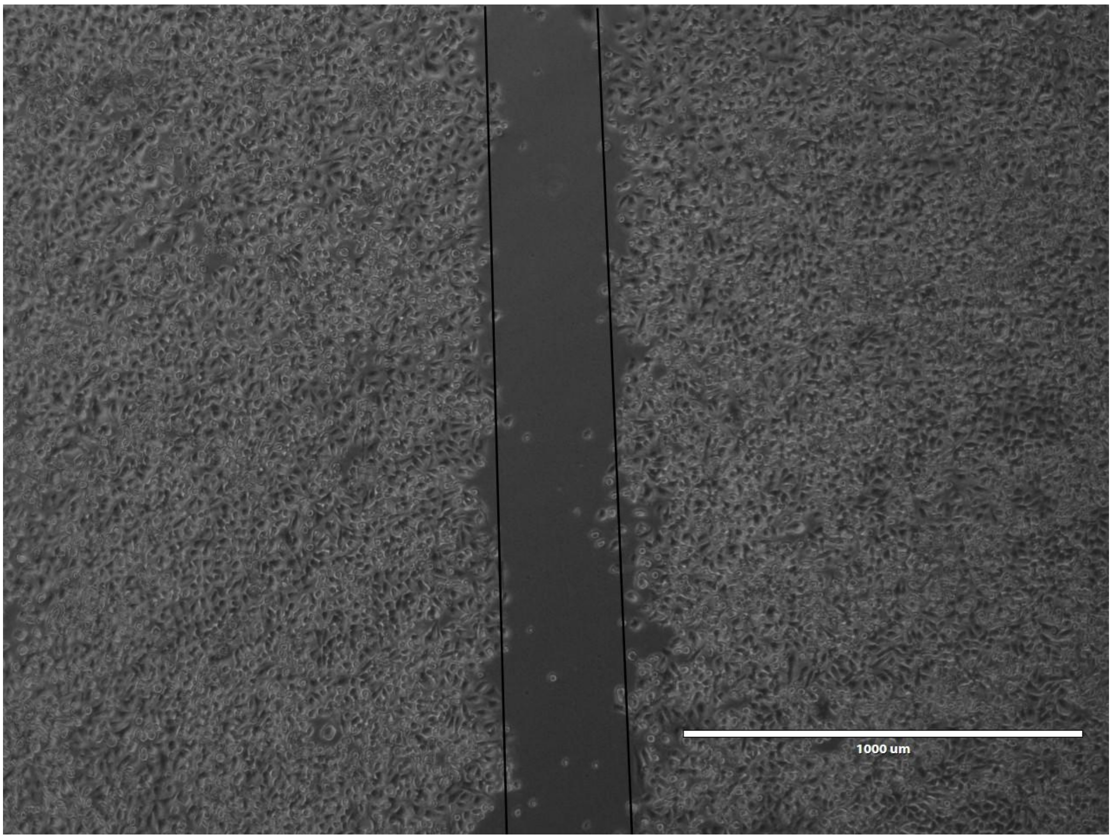

4B

0-skov3-control

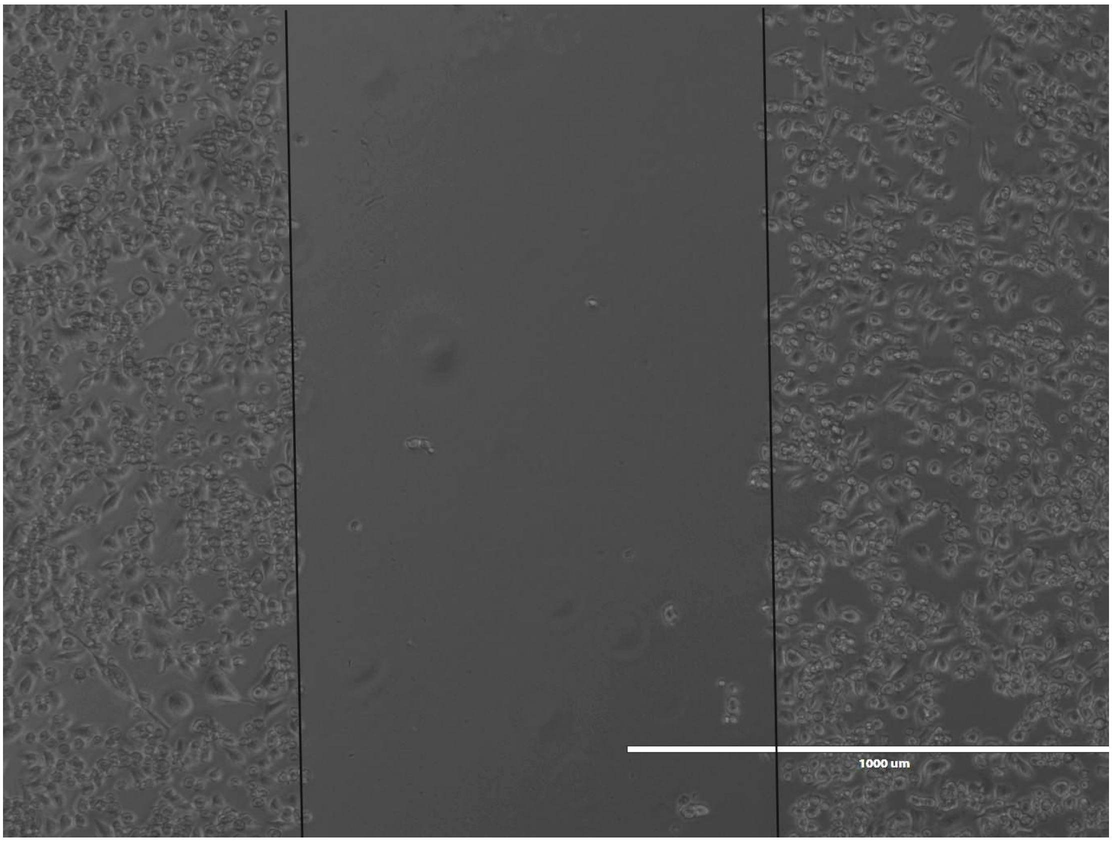

0-skov3-sh

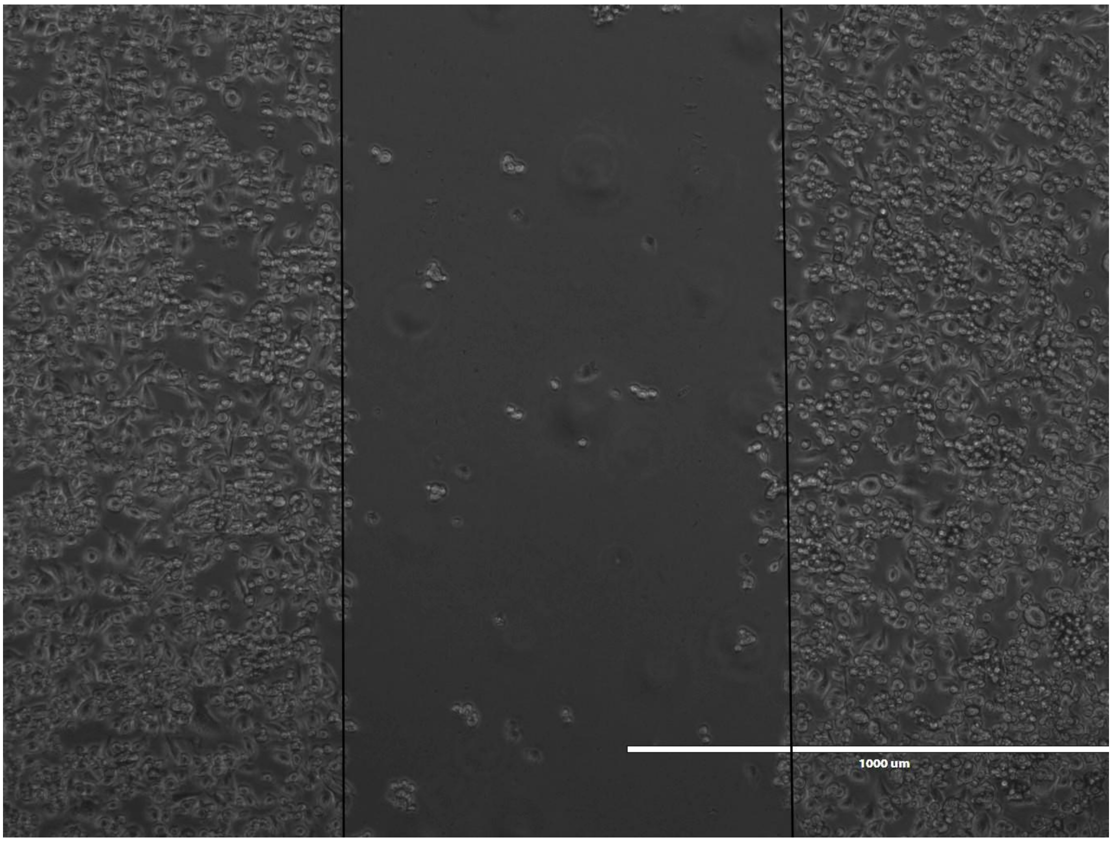

24 skov3-control

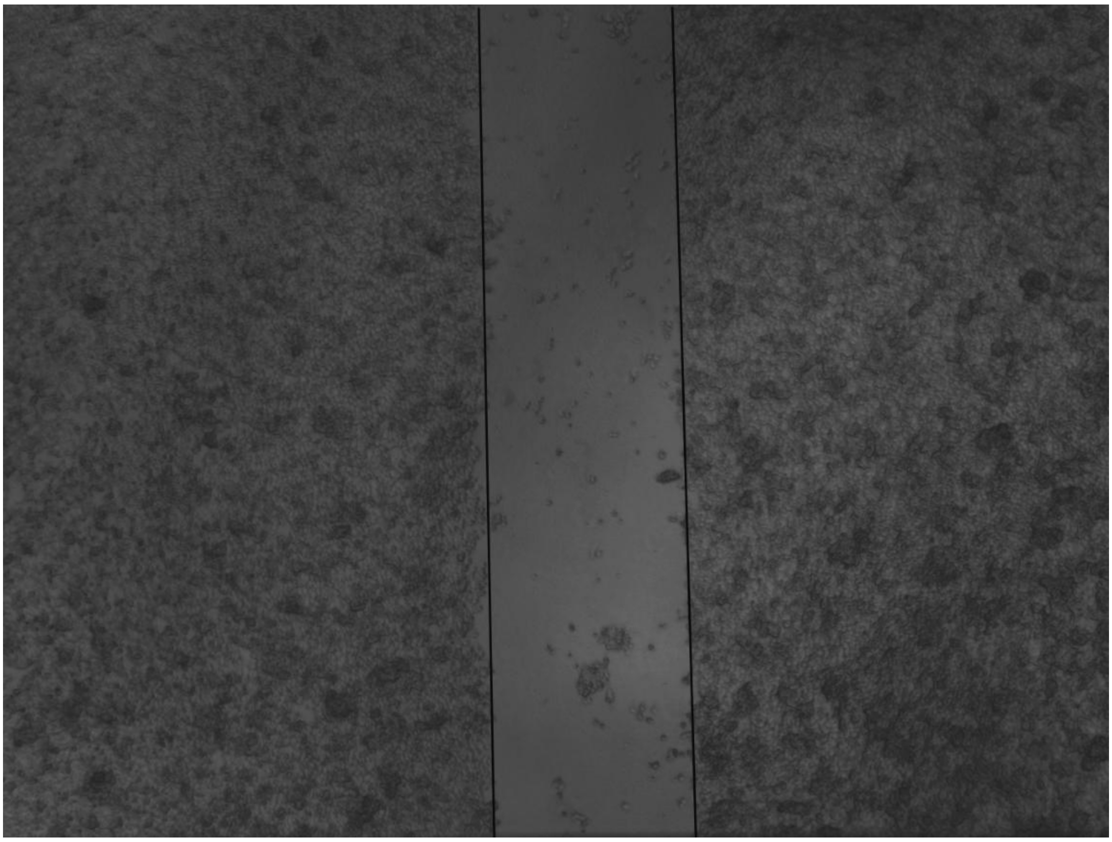

24 skov3-sh

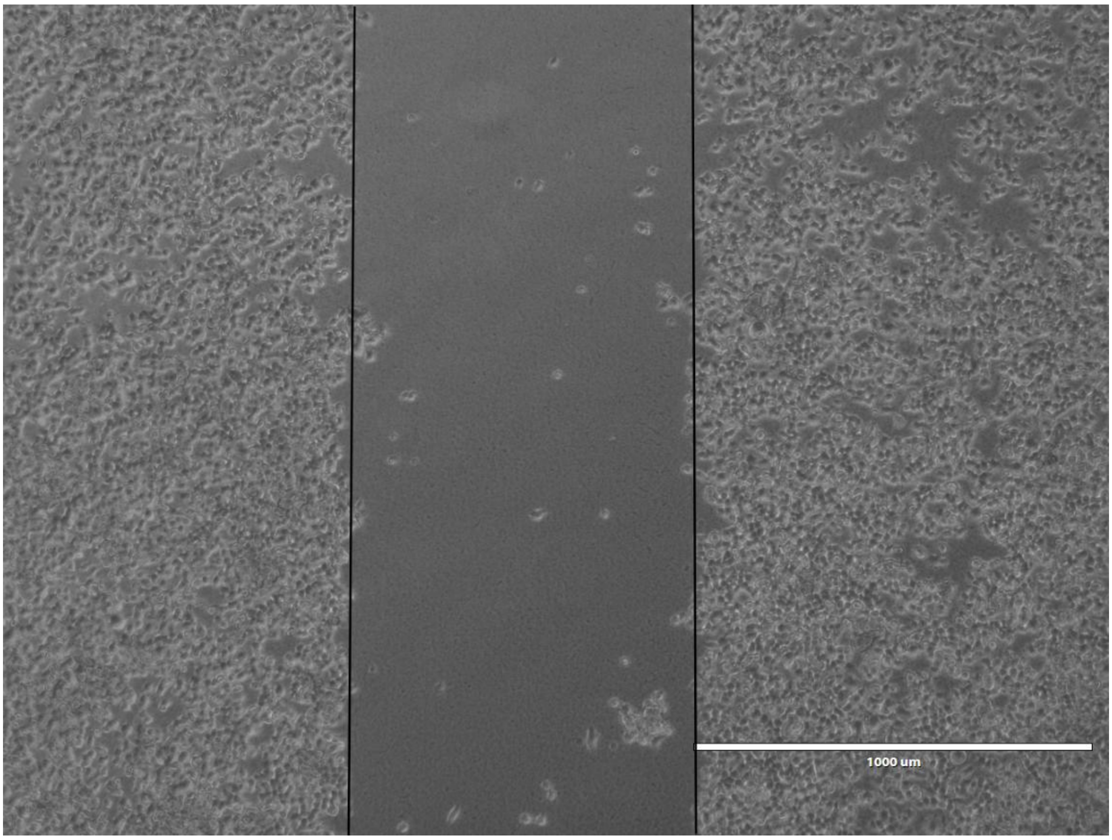

8910-0-1

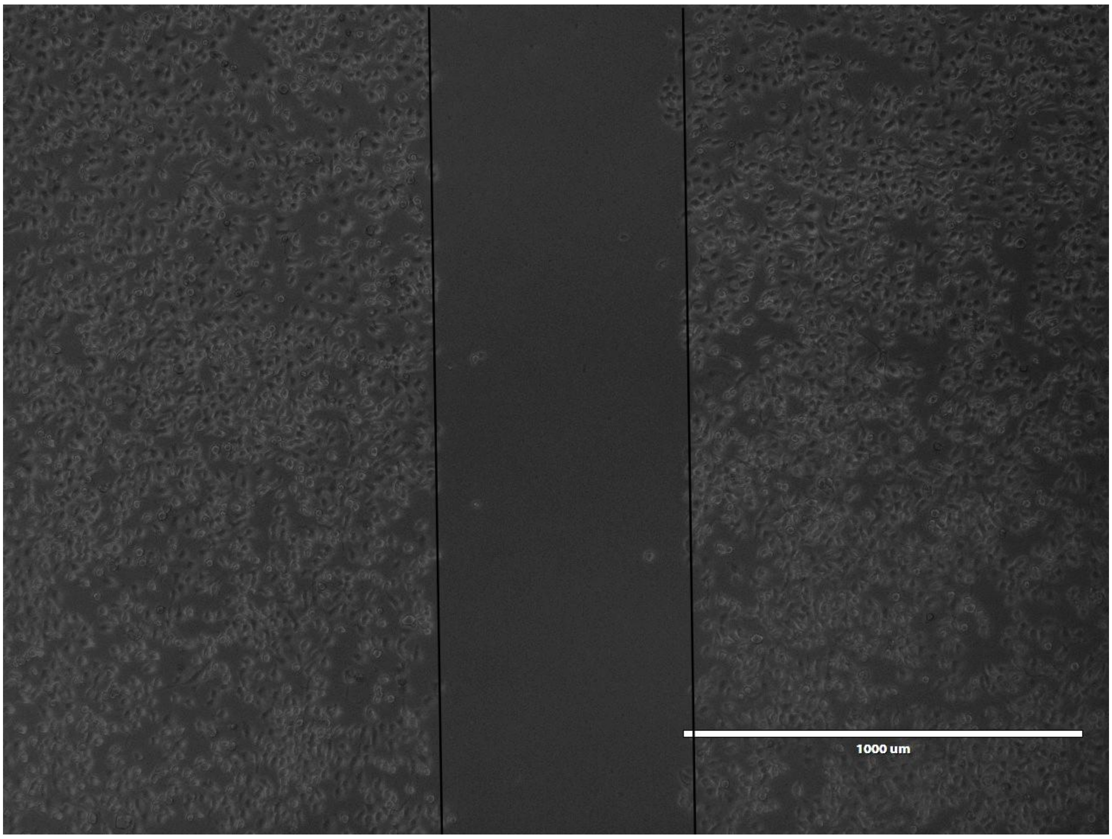

8910-0-2

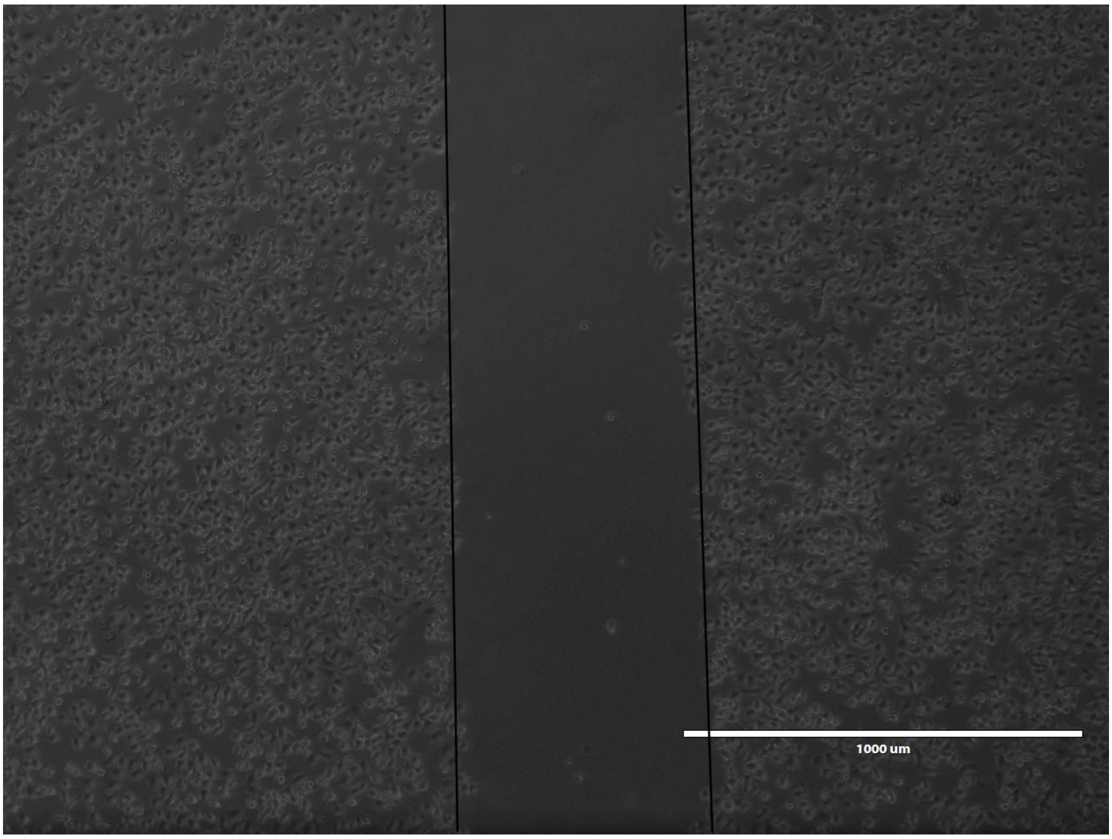

8910-24-1

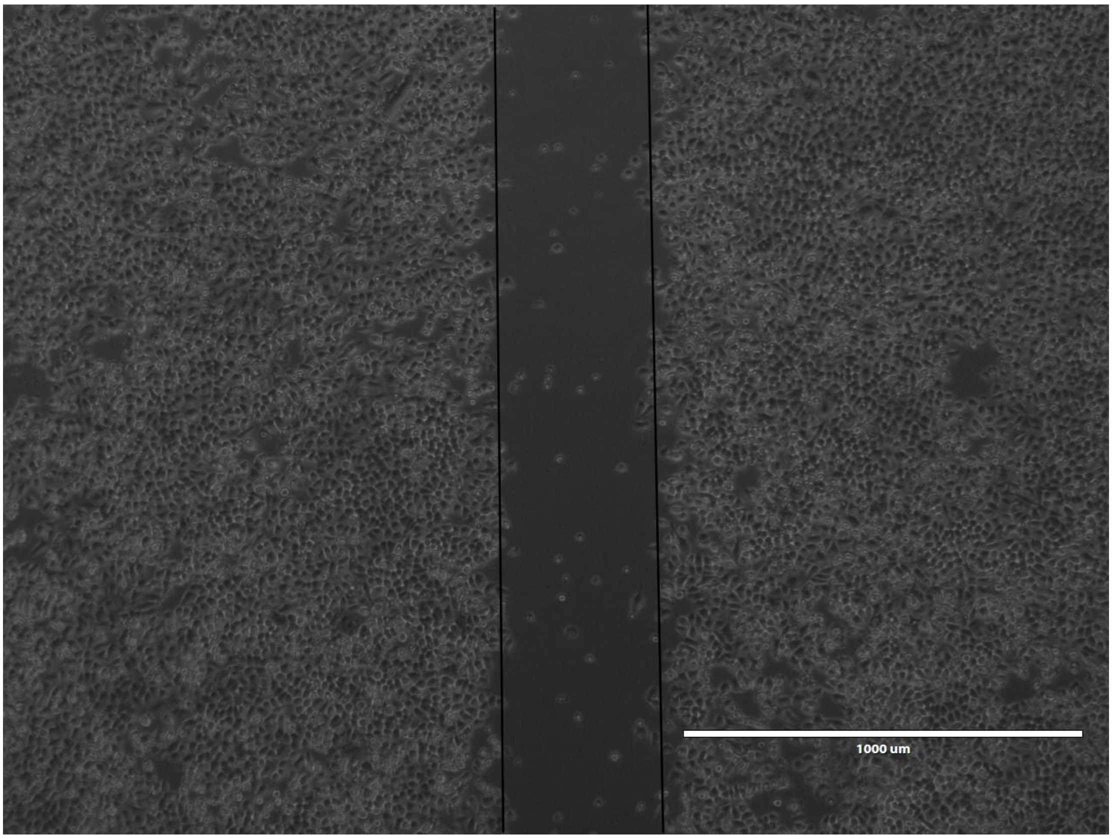

8910-24-2

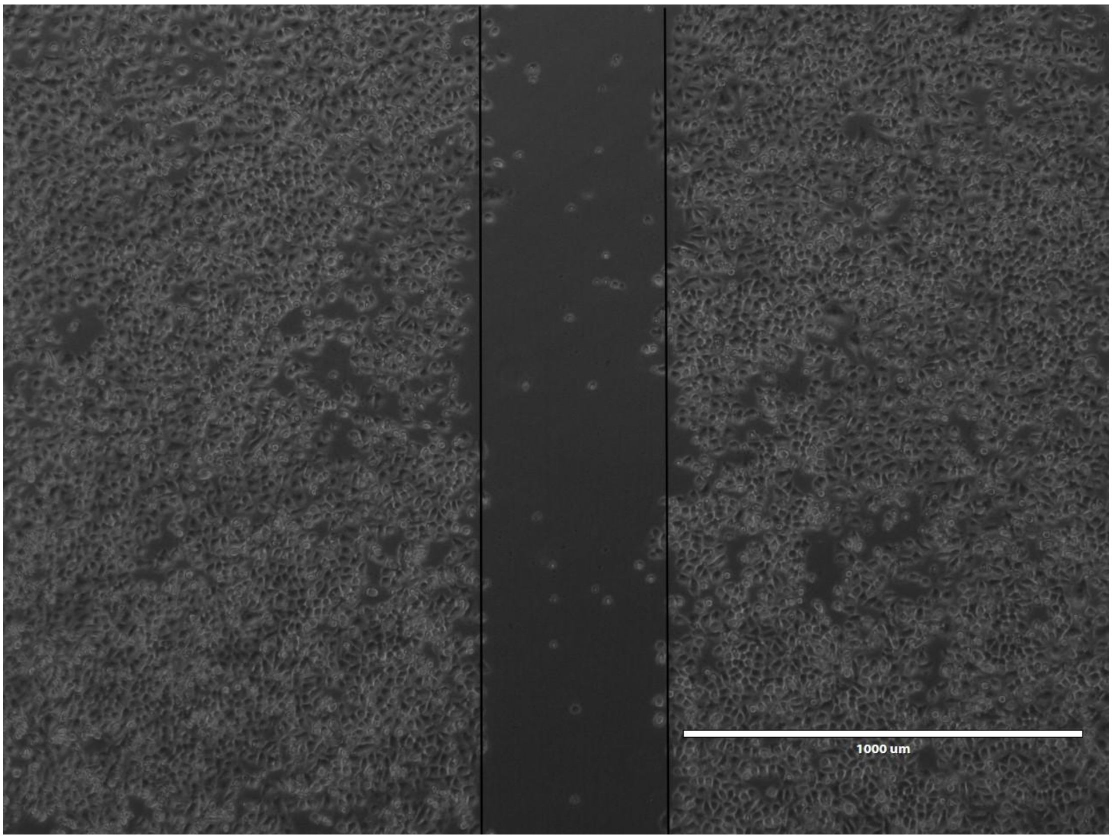

Supplement: Supplementary file 8 [file DataSheet5.PDF]
