# Supplementary material for: Circular RNA CDR1as Alleviates Cisplatin-Based Chemoresistance by Suppressing MiR-1299 in Ovarian Cancer
Source: Front Genet. 2022 Jan 26;12:815448. doi: 10.3389/fgene.2021.815448 (PMC8826532; doi:10.3389/fgene.2021.815448)
Supplement: Supplementary file 9 [file DataSheet8.PDF]

2E

HO8910-CONTROL

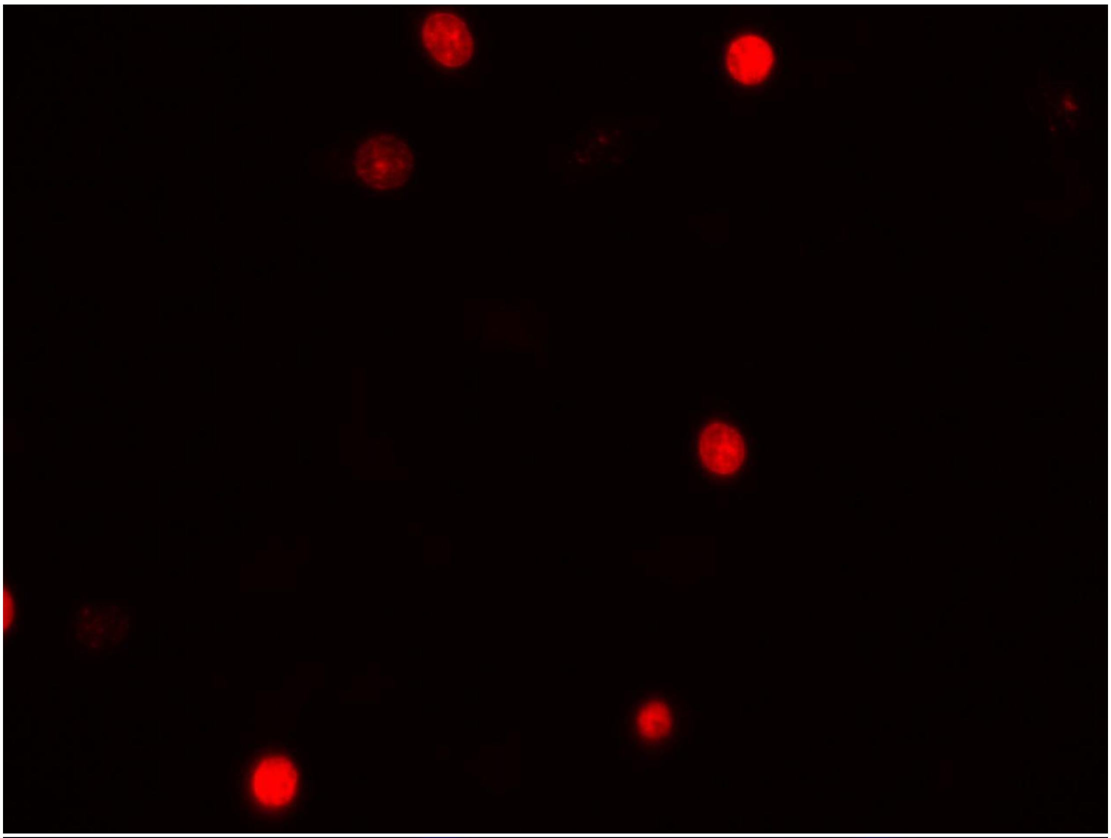

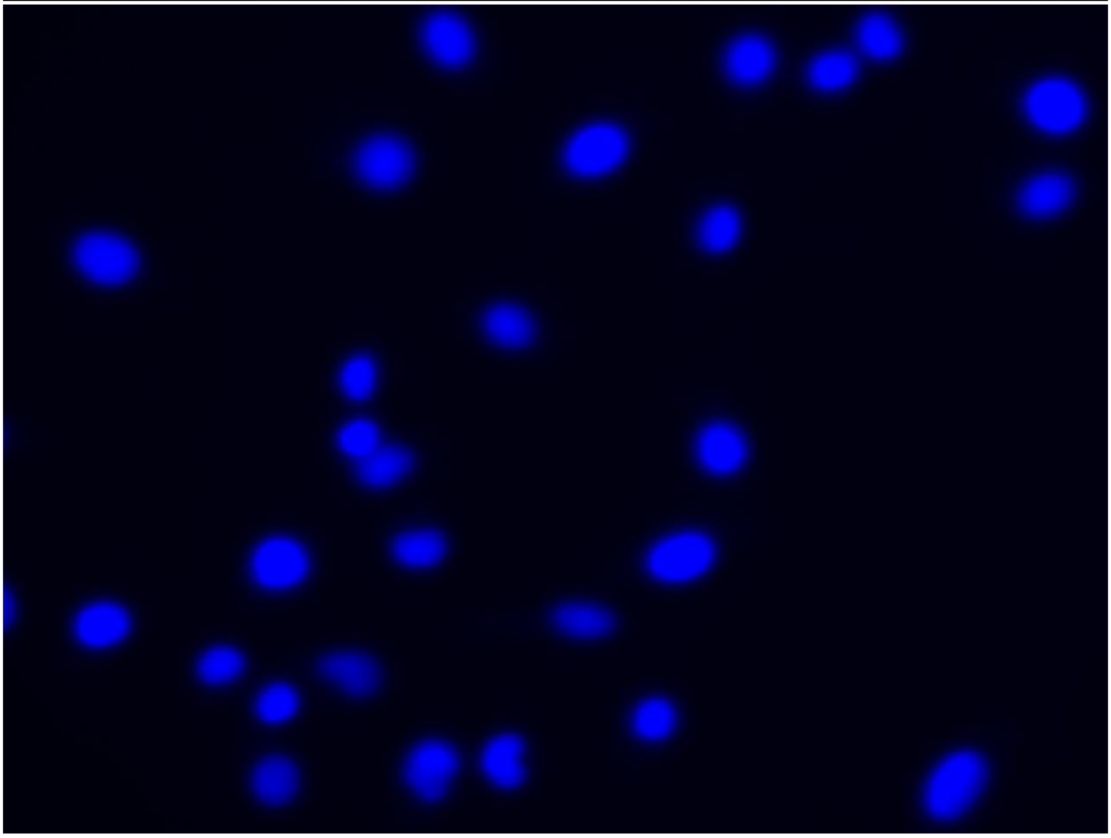

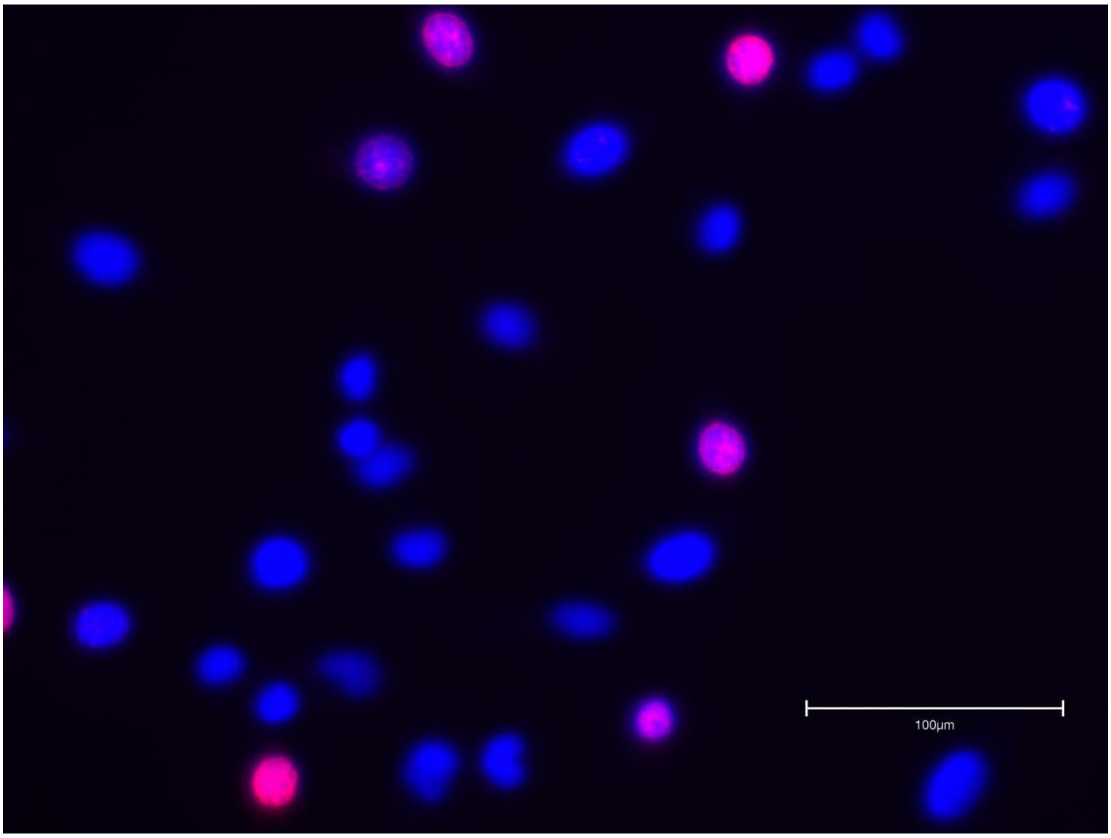

HO8910-sh

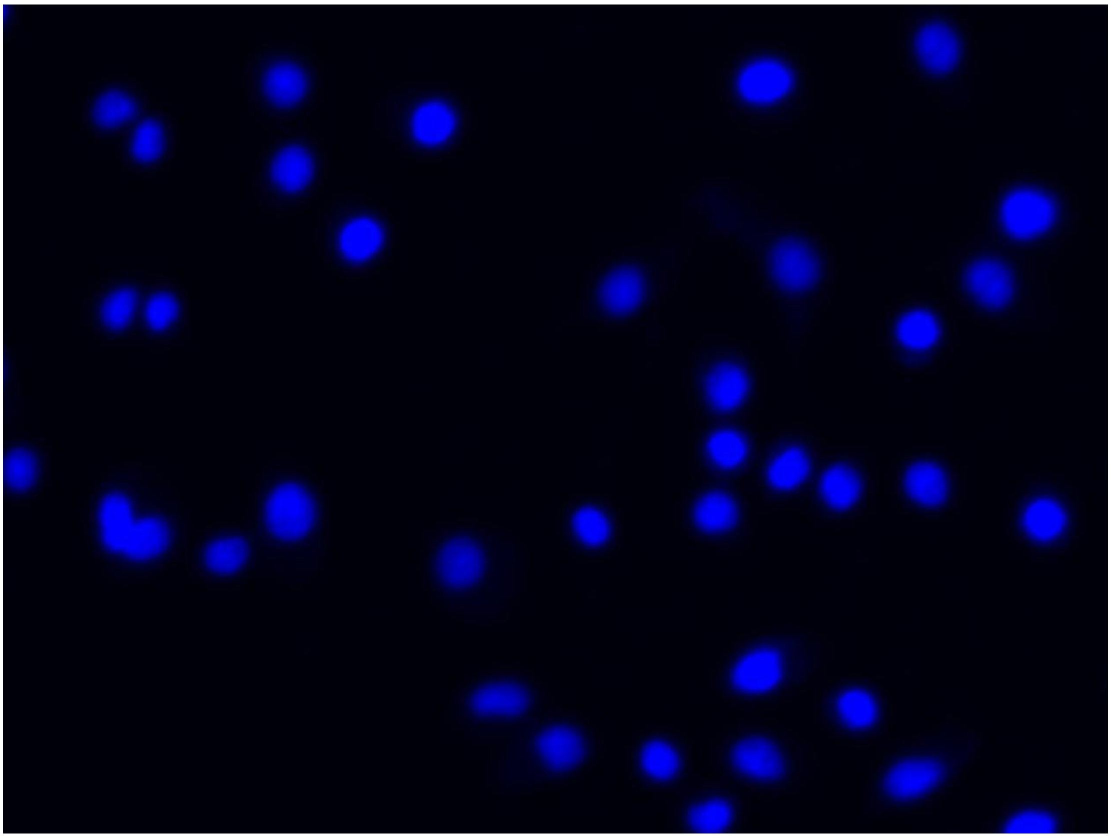

SKOV3-CONTROL

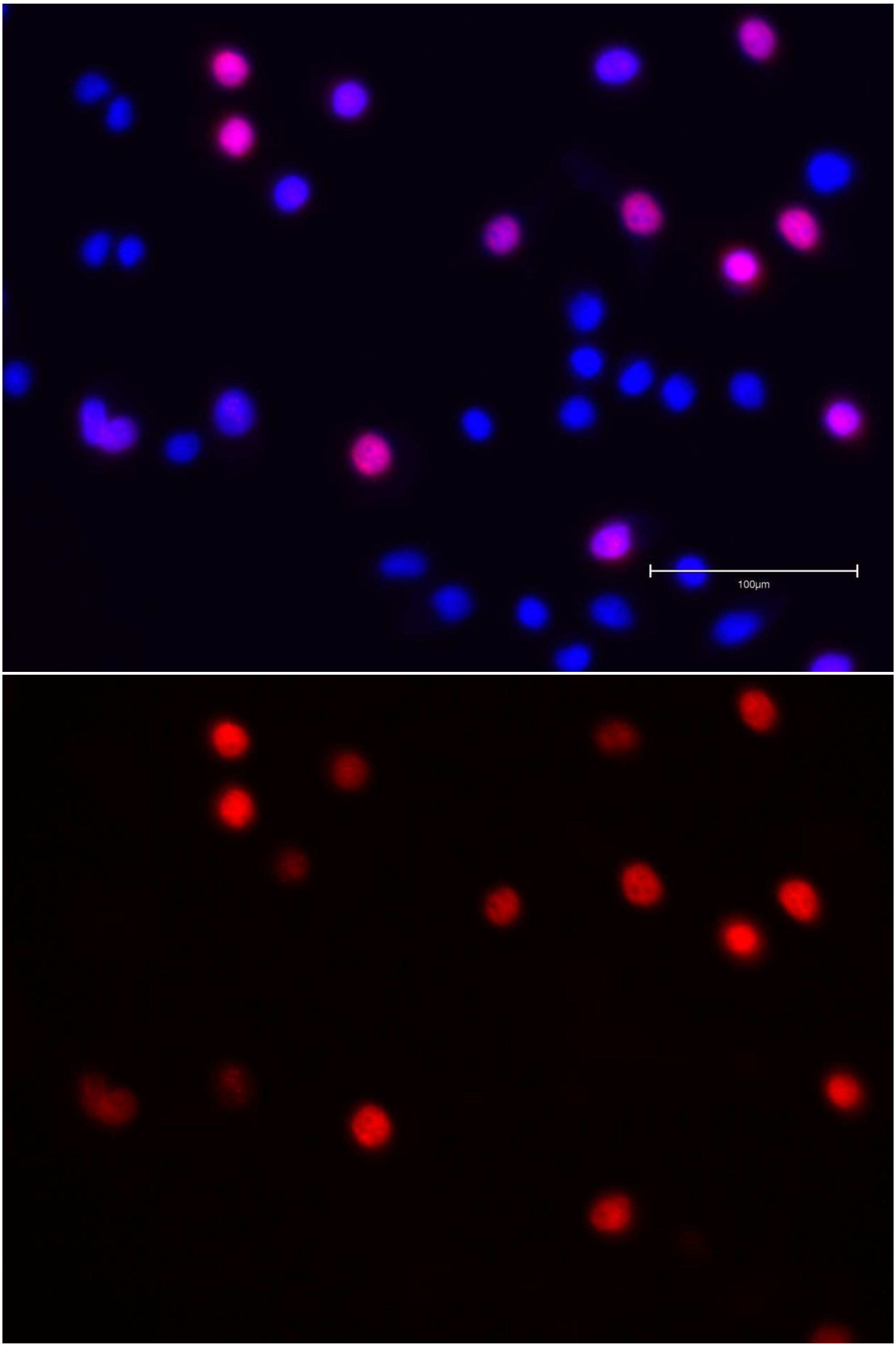

SKOV3-CONTROL

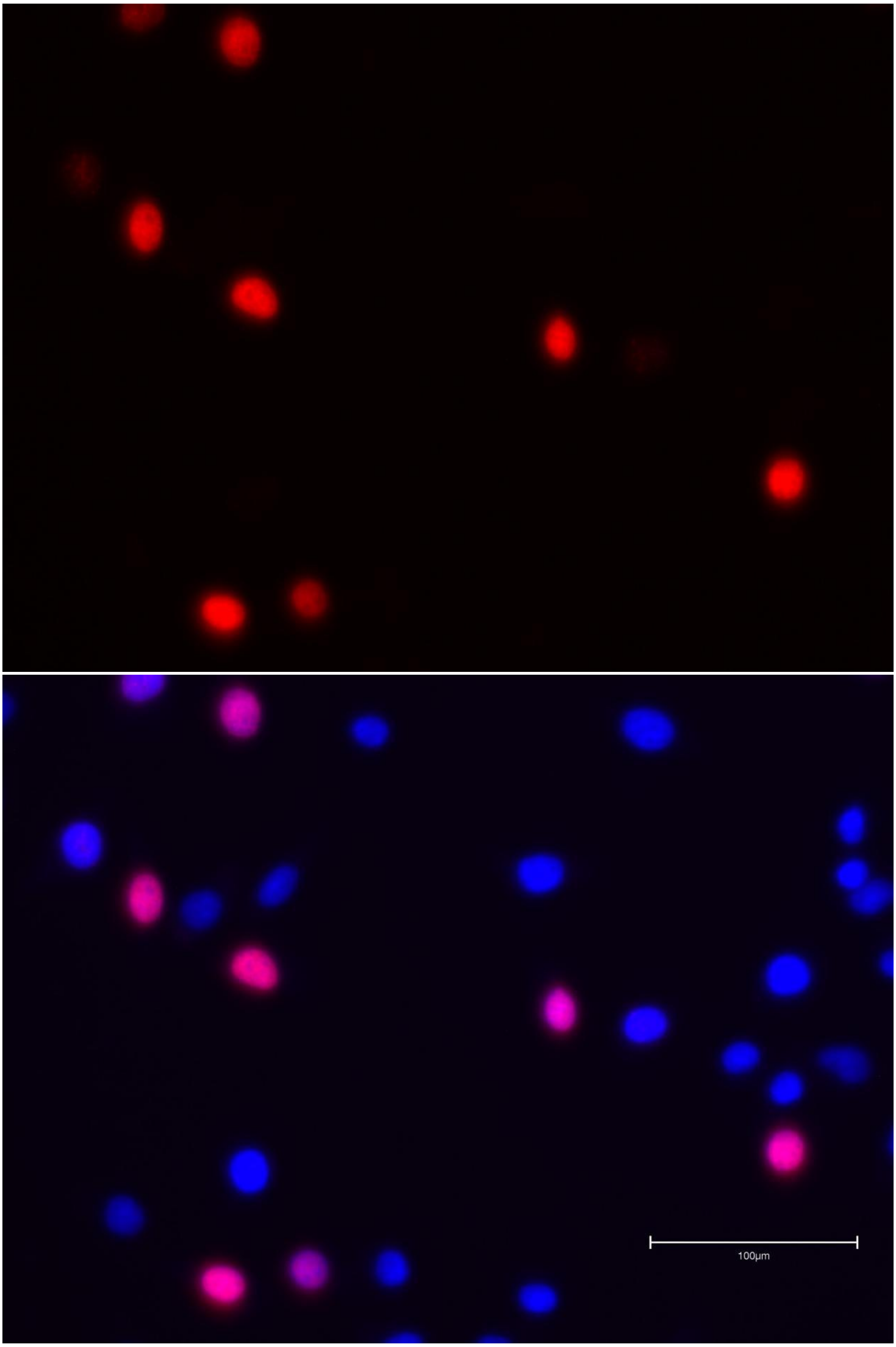
100µm

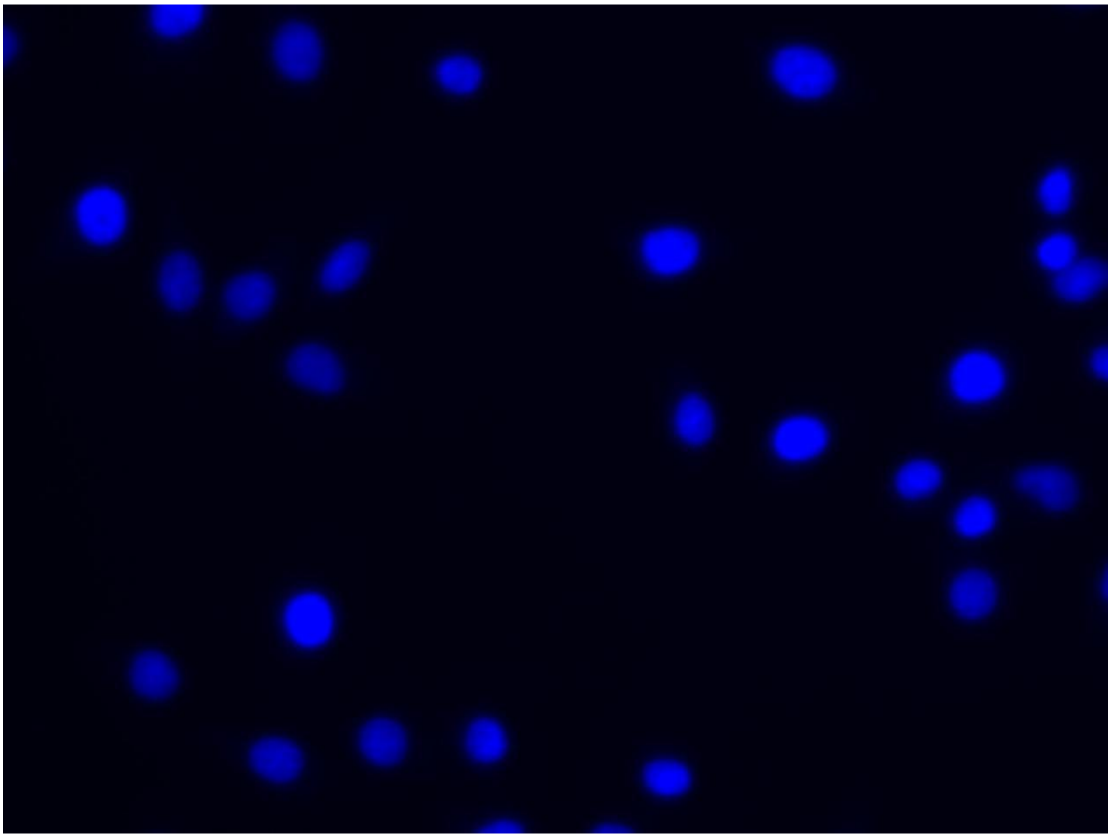

SKOV3-sh

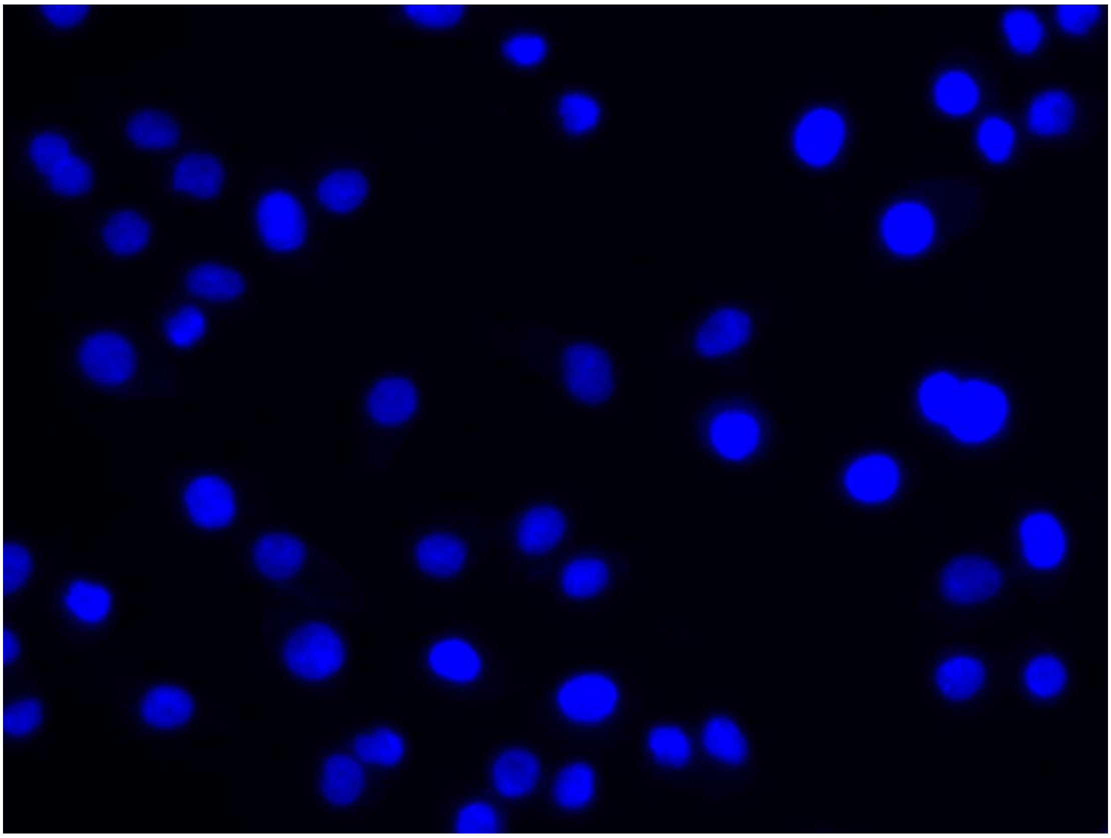

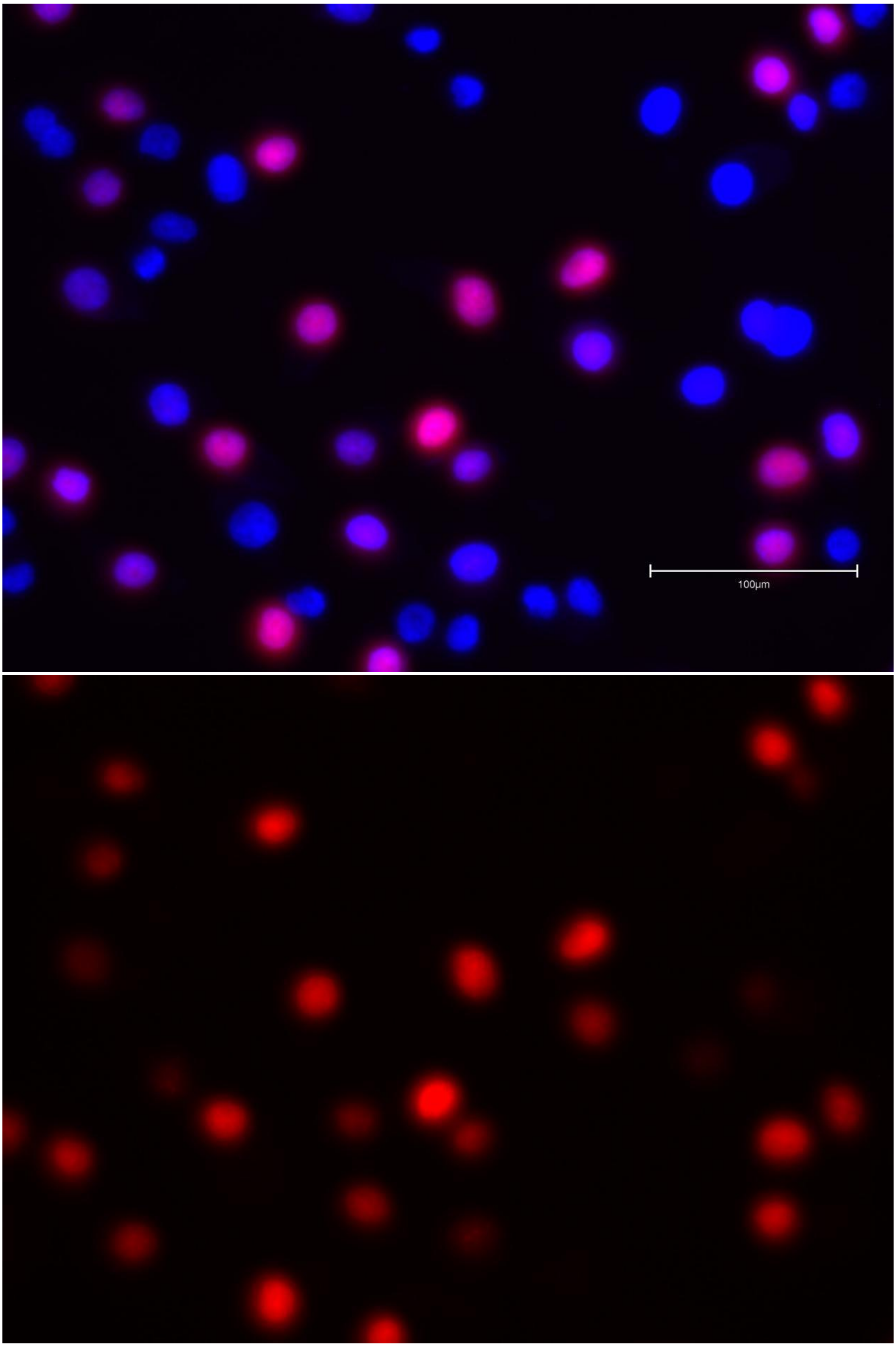

2H

HO8910/DDP-CONTROL

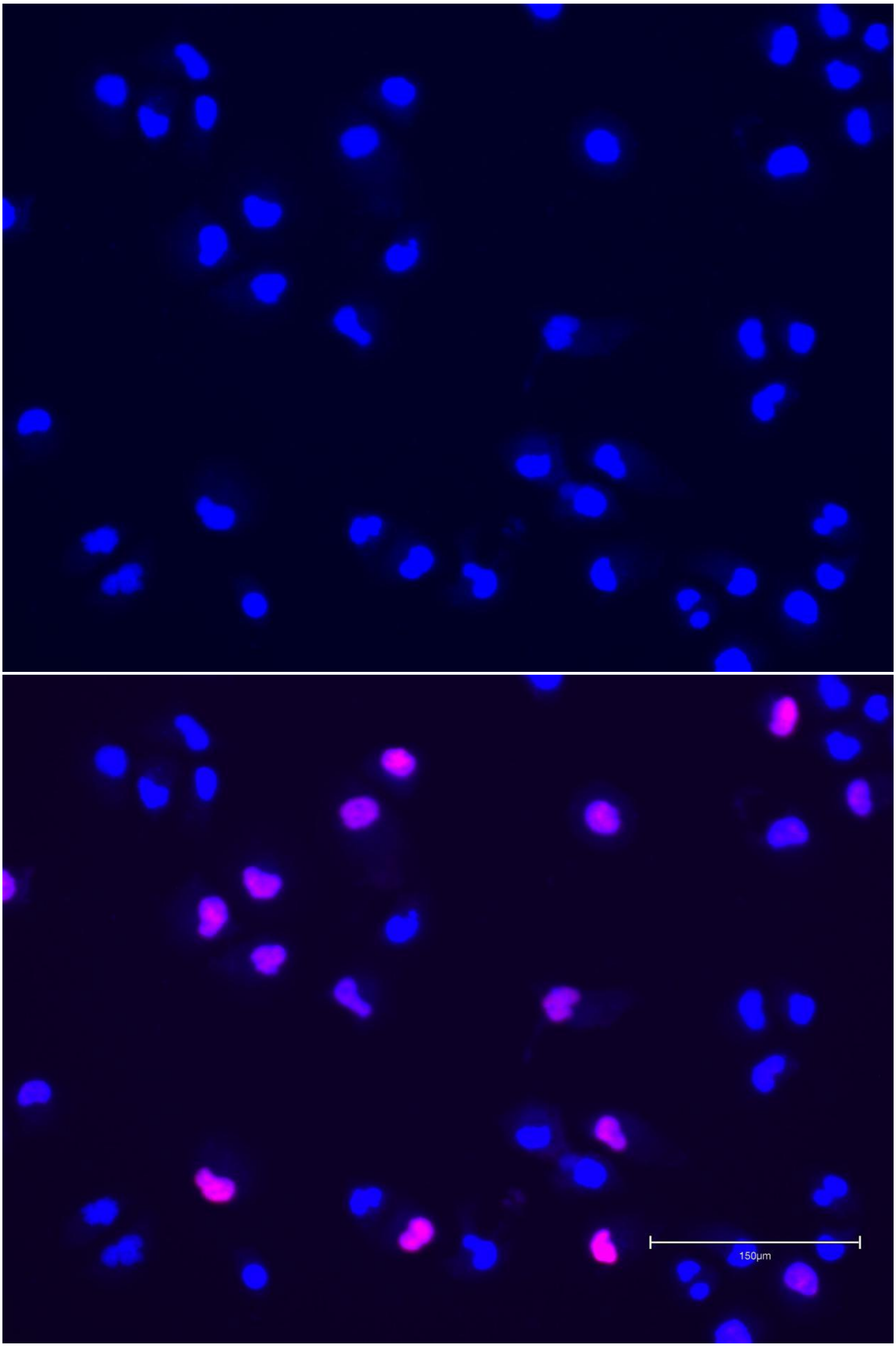
150μm

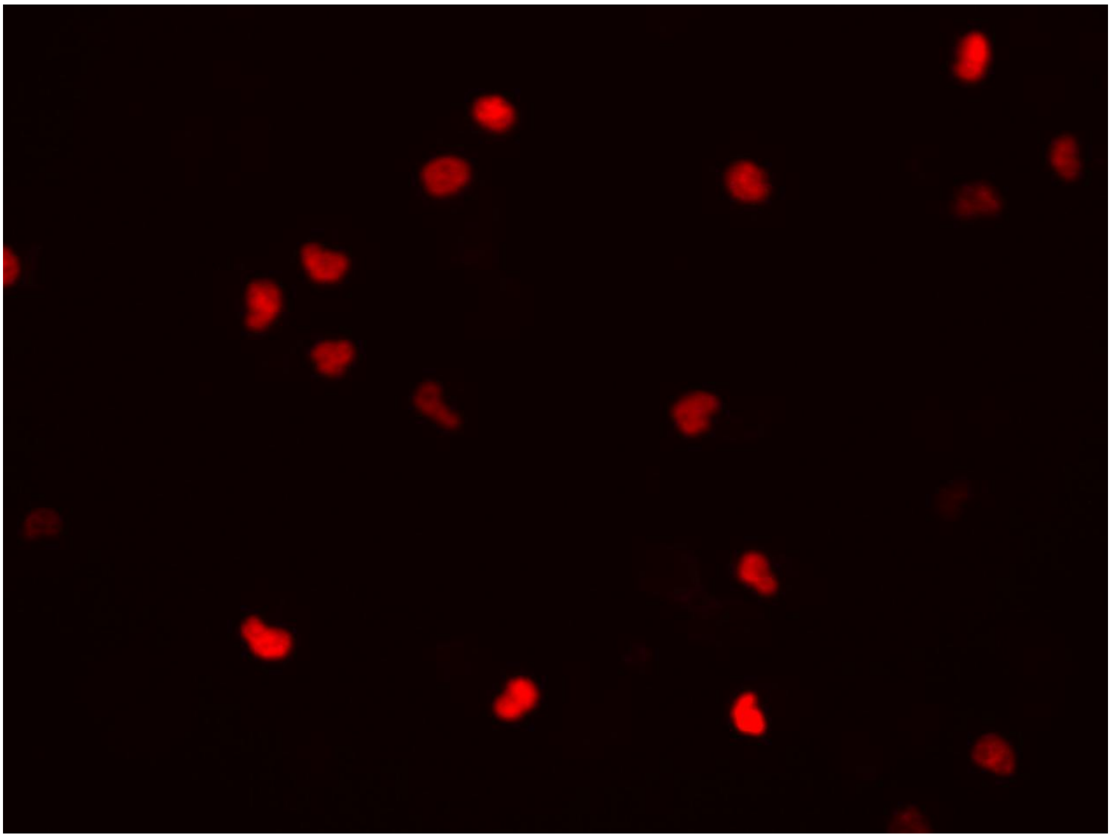

HO8910/DDP-h

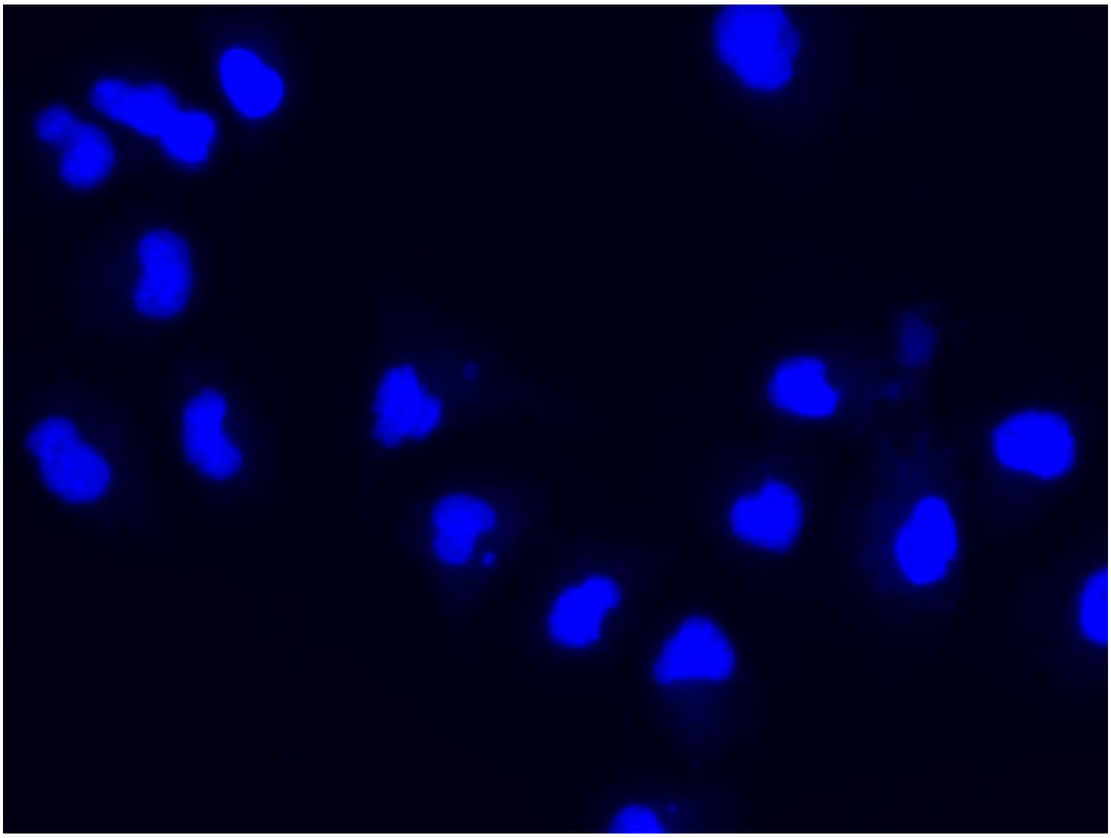

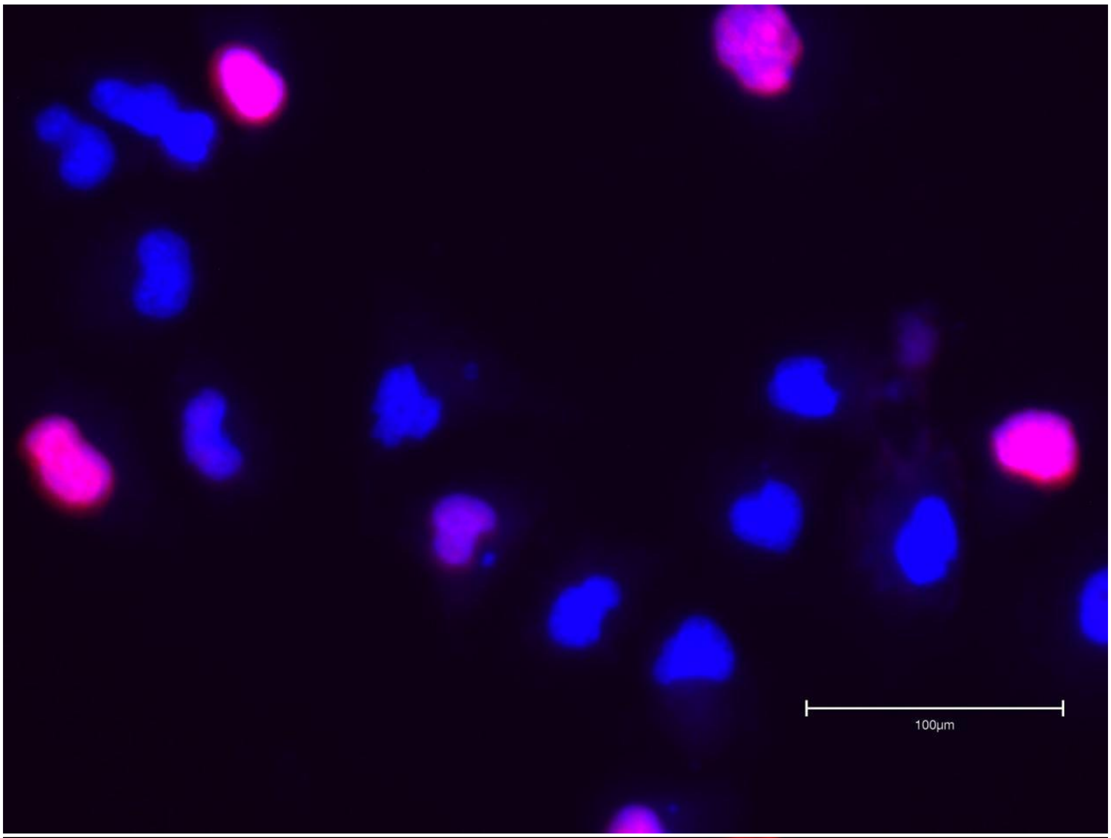

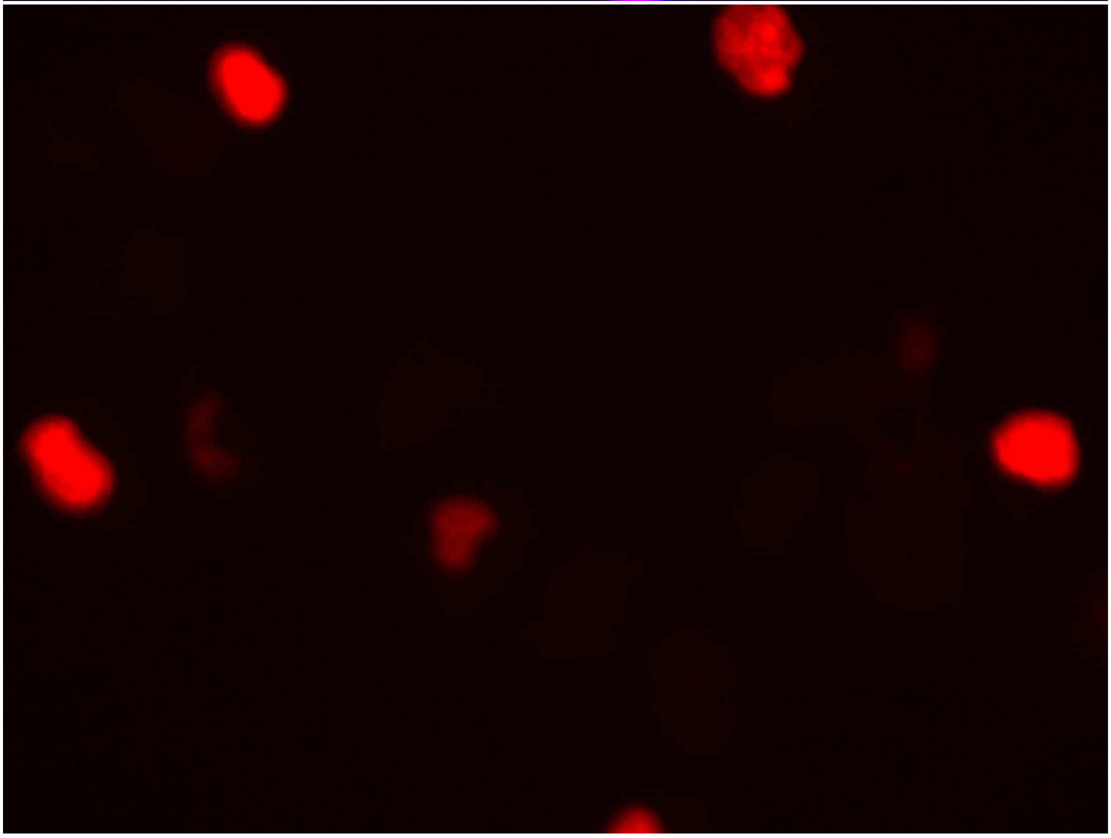

SKOV3/DDP-CONTROL

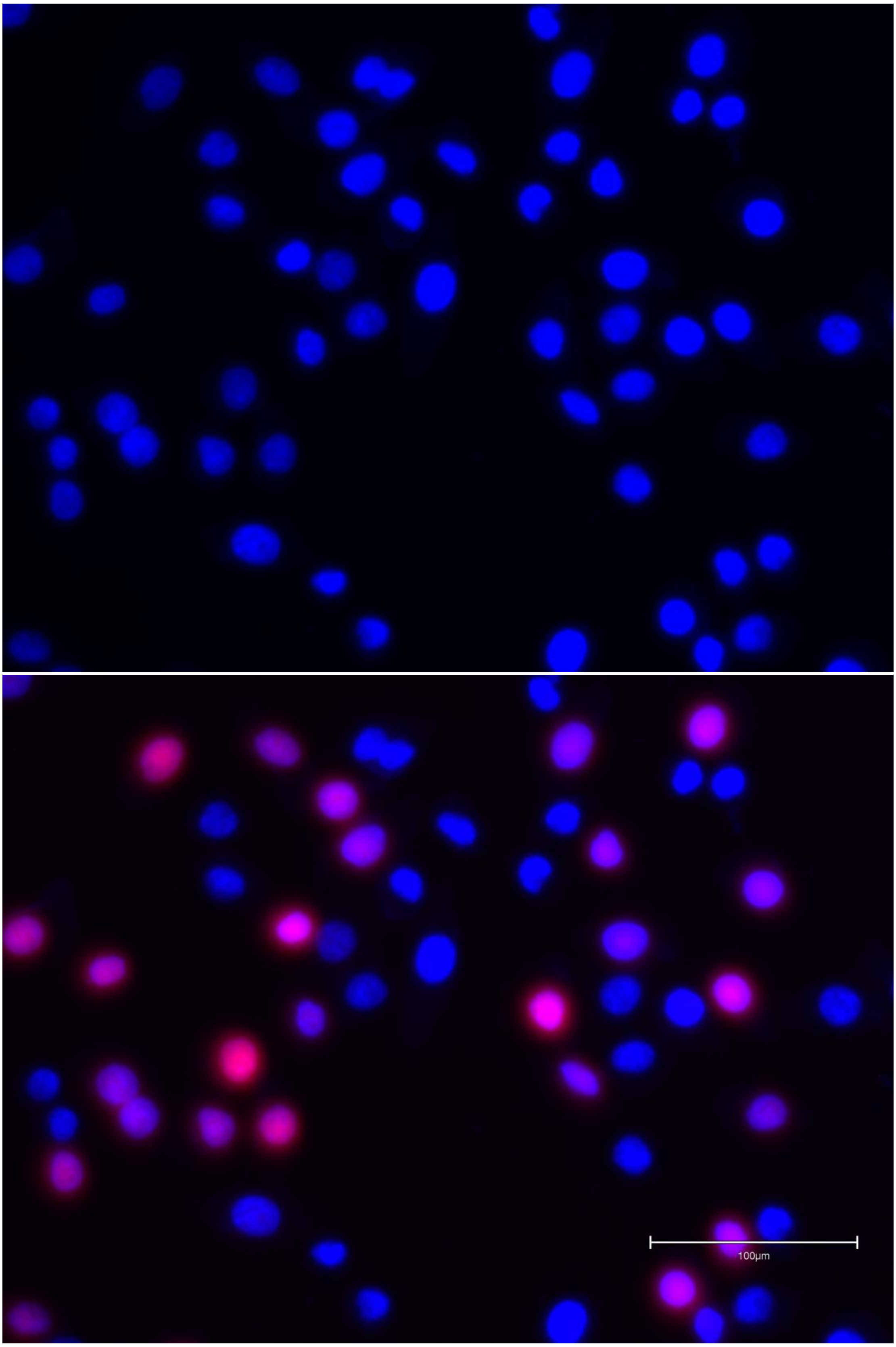
100µm

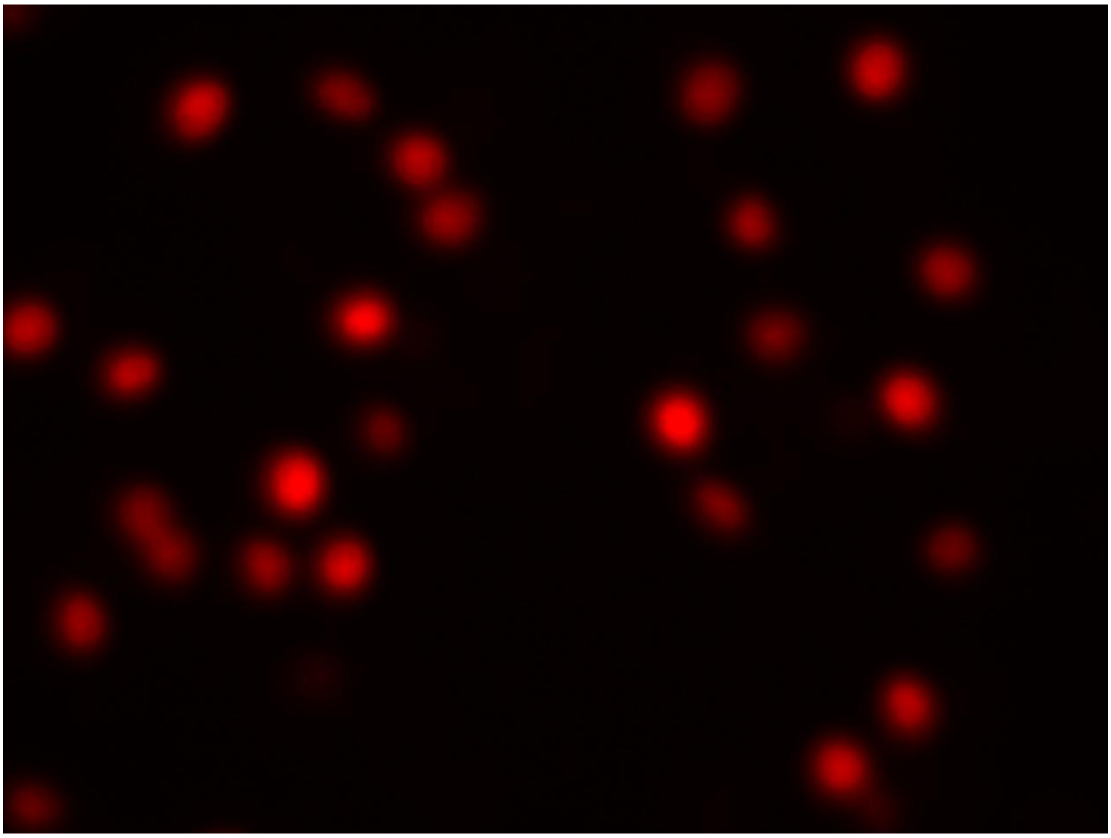

SKOV3/DDP-h

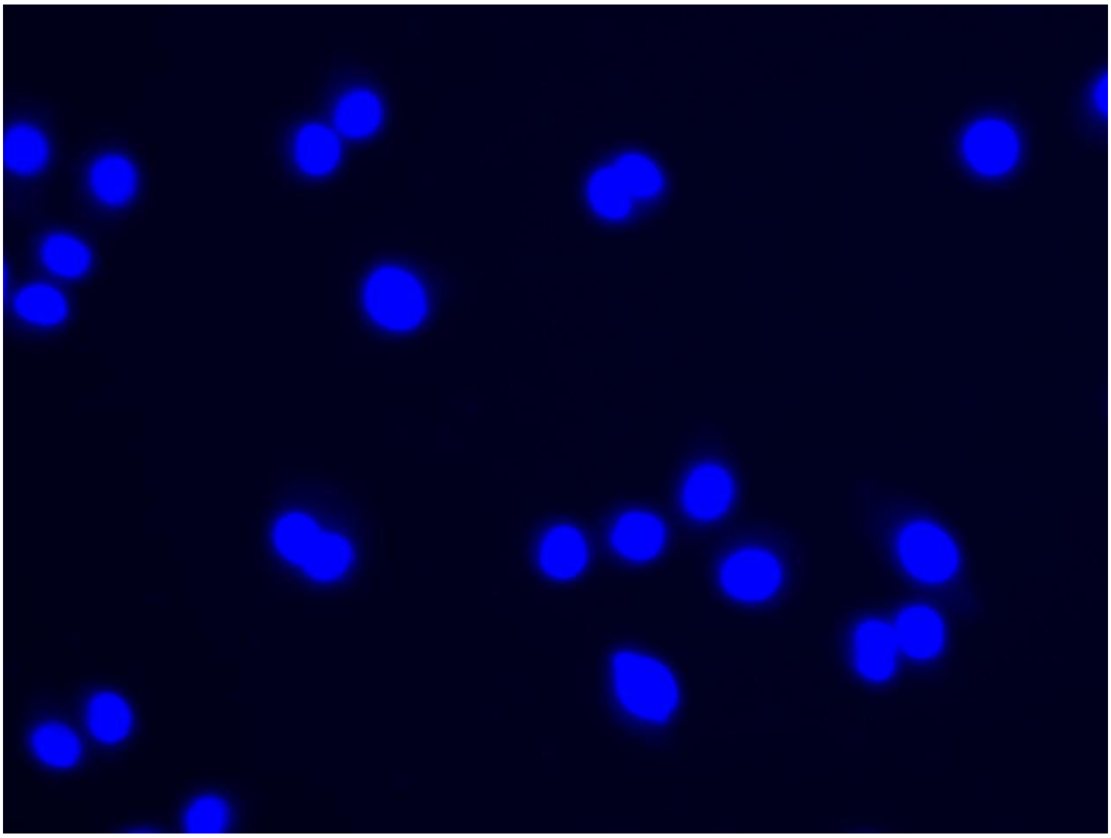

7C

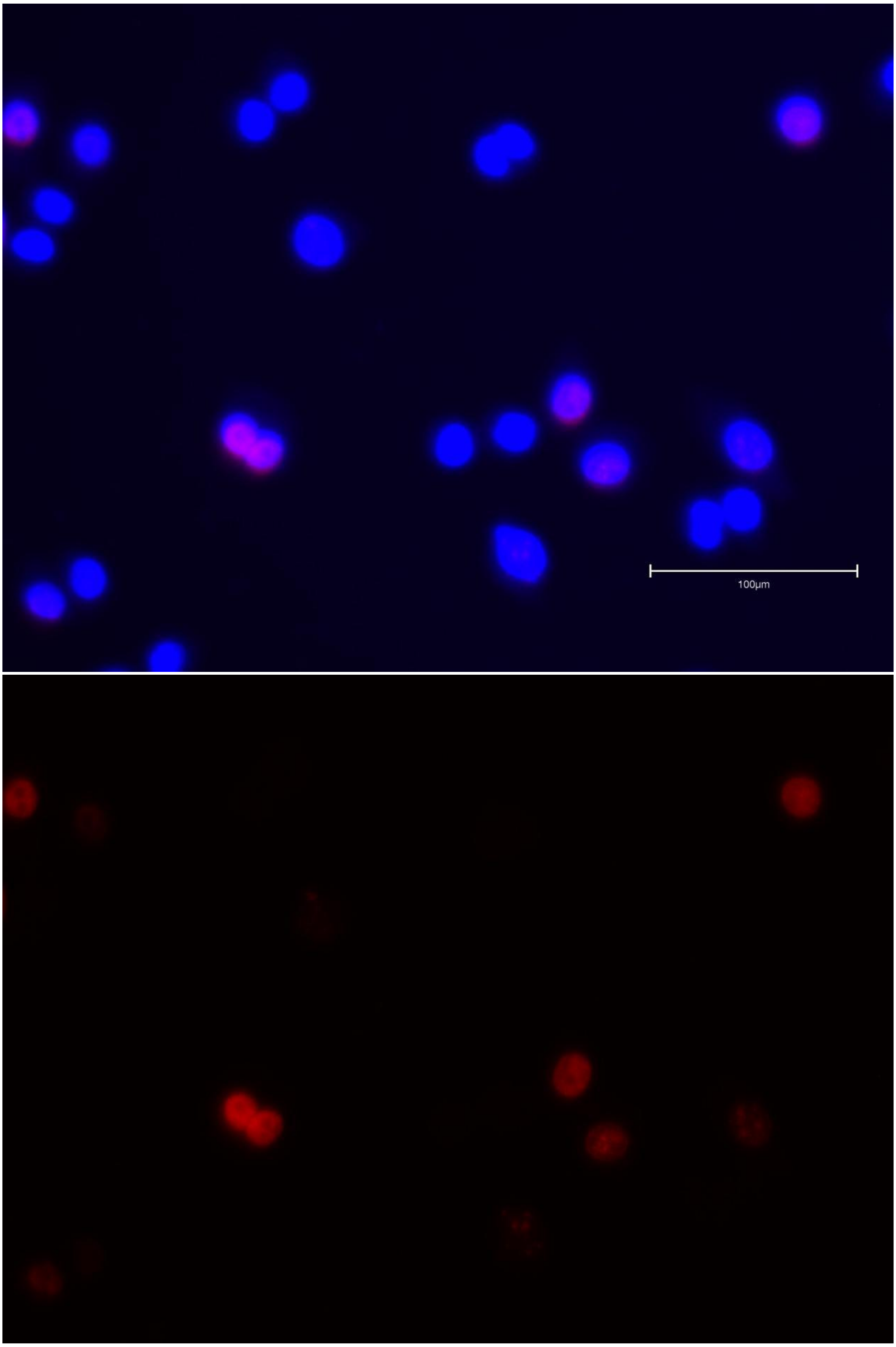

7C

HO8910-control+1299

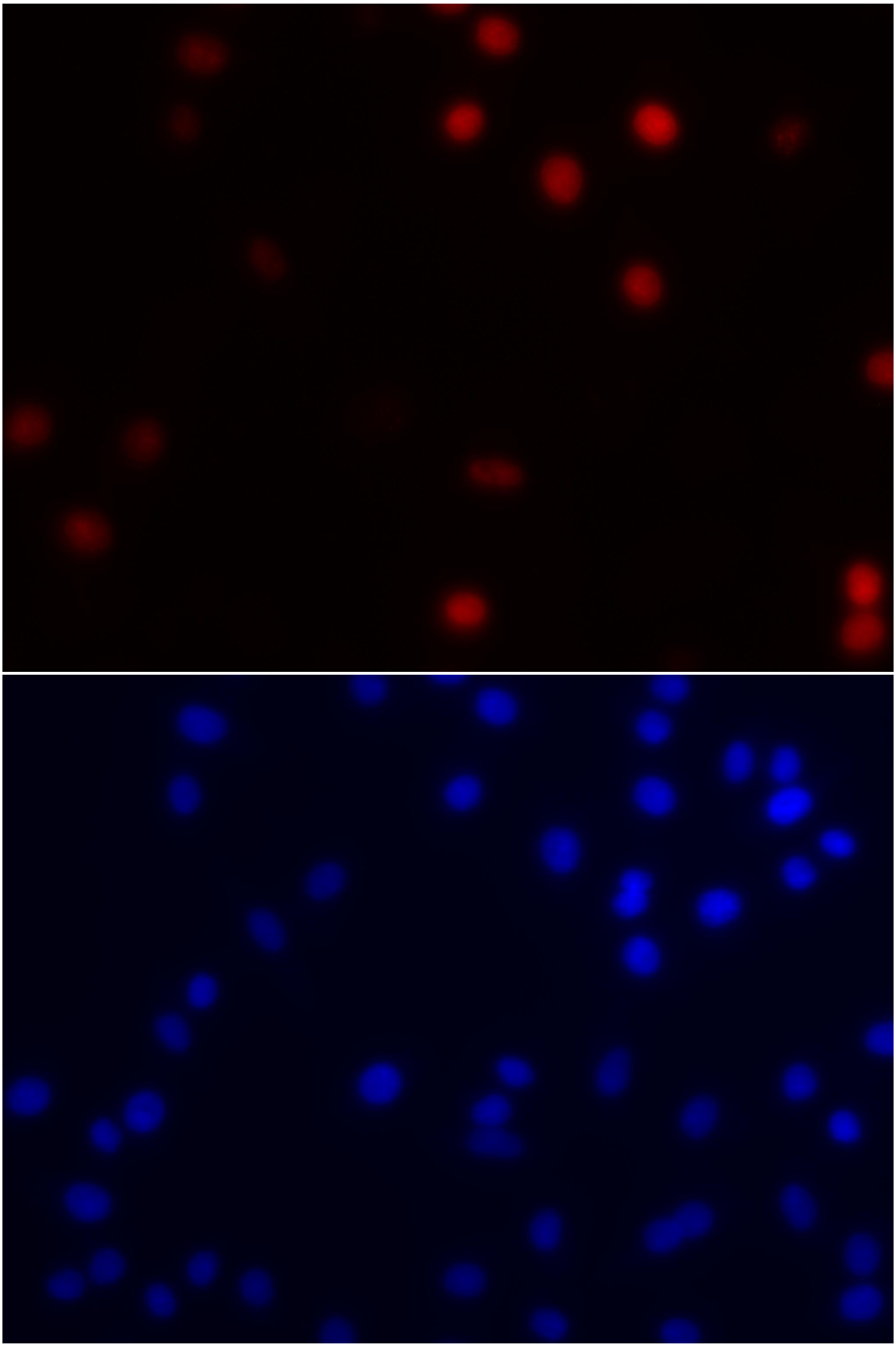

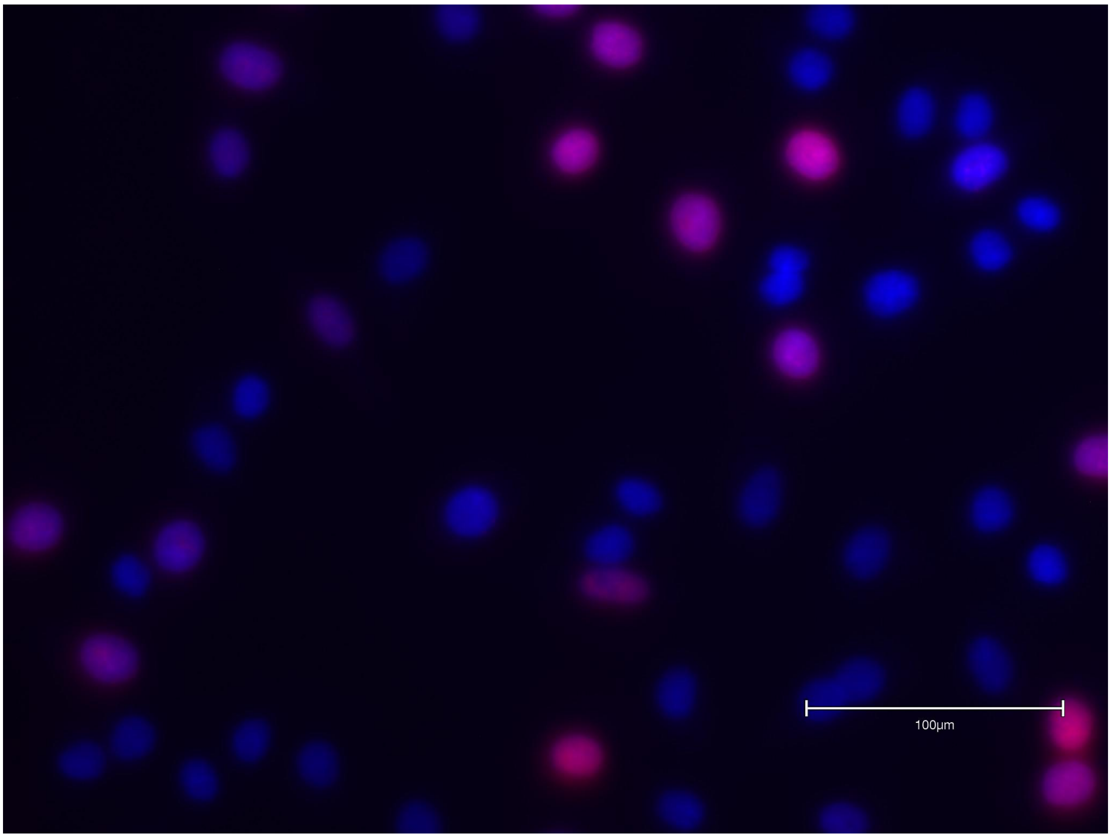

HO8910-SH-CDR1AS+NC

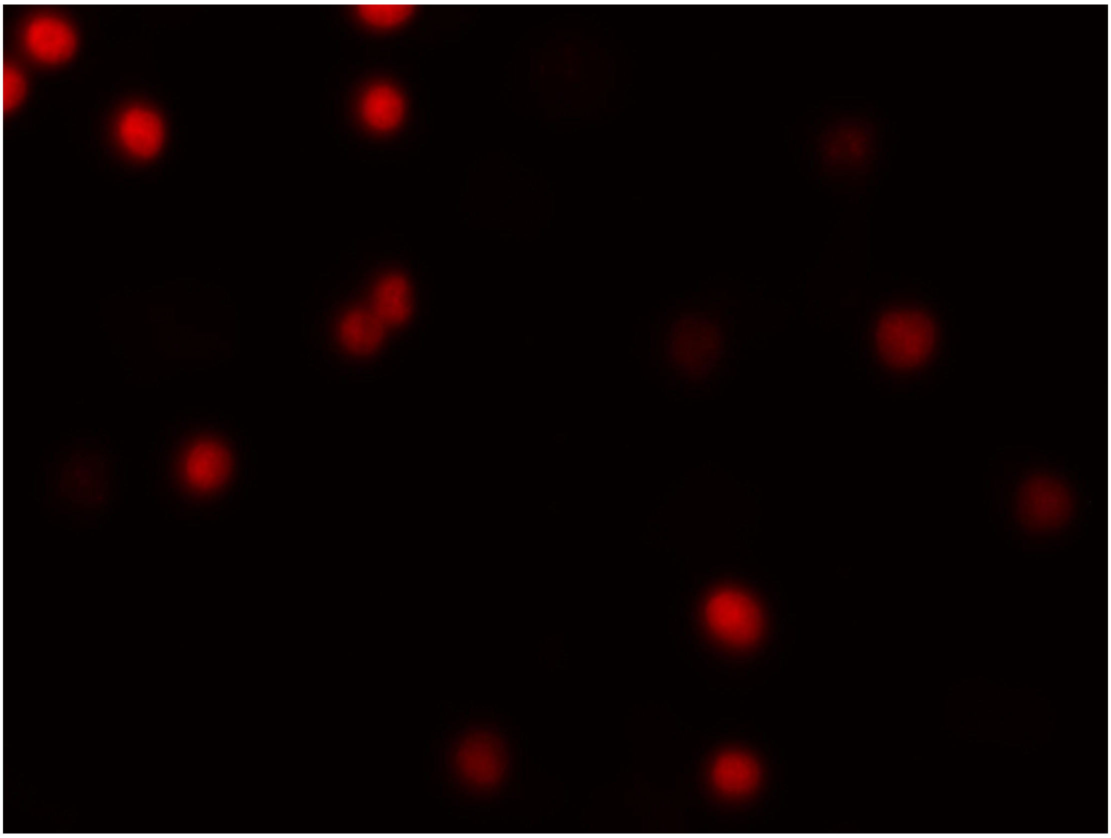

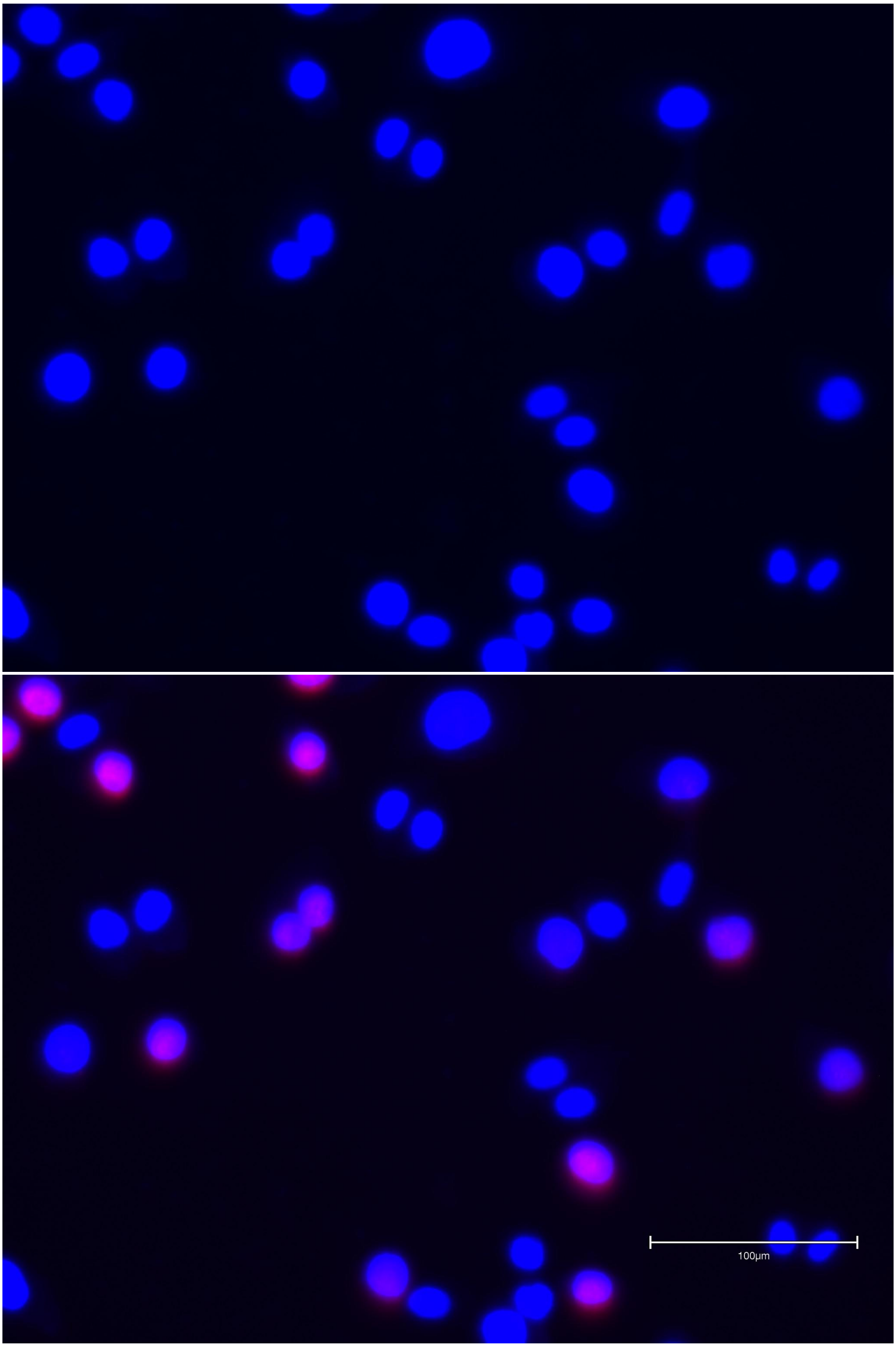

HO8910-CONTROL+NC

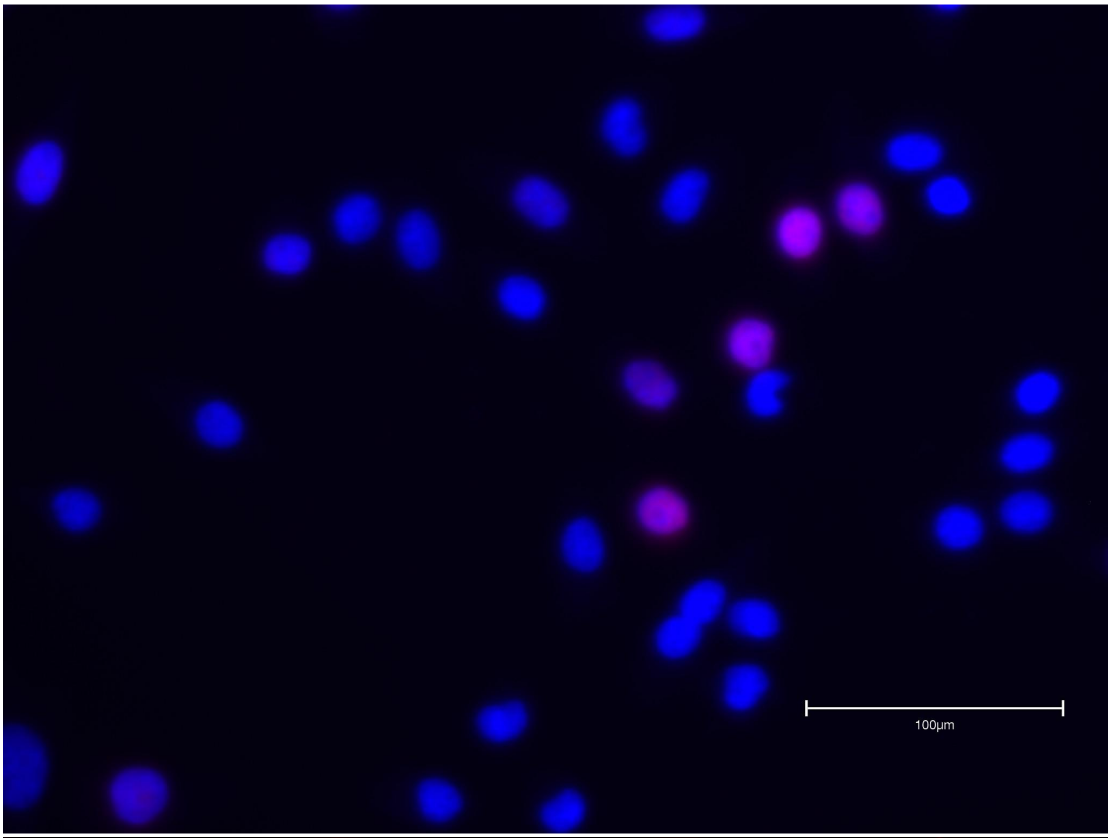
100µm

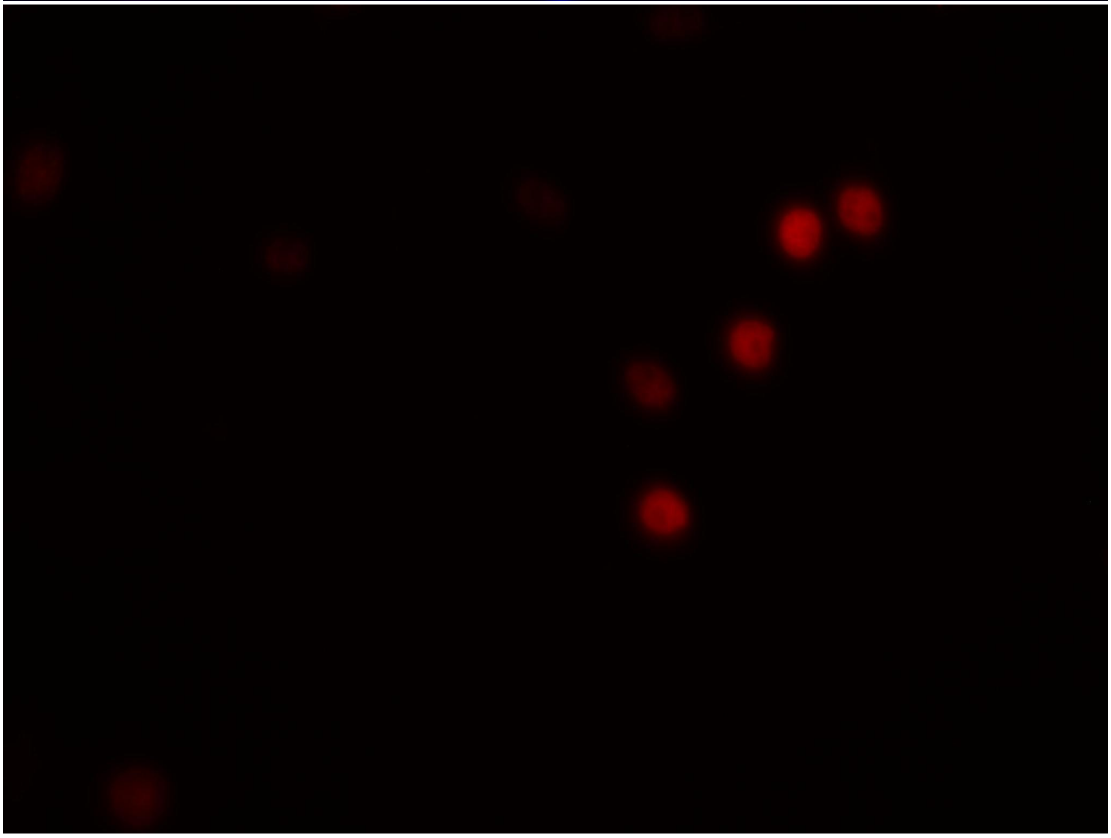

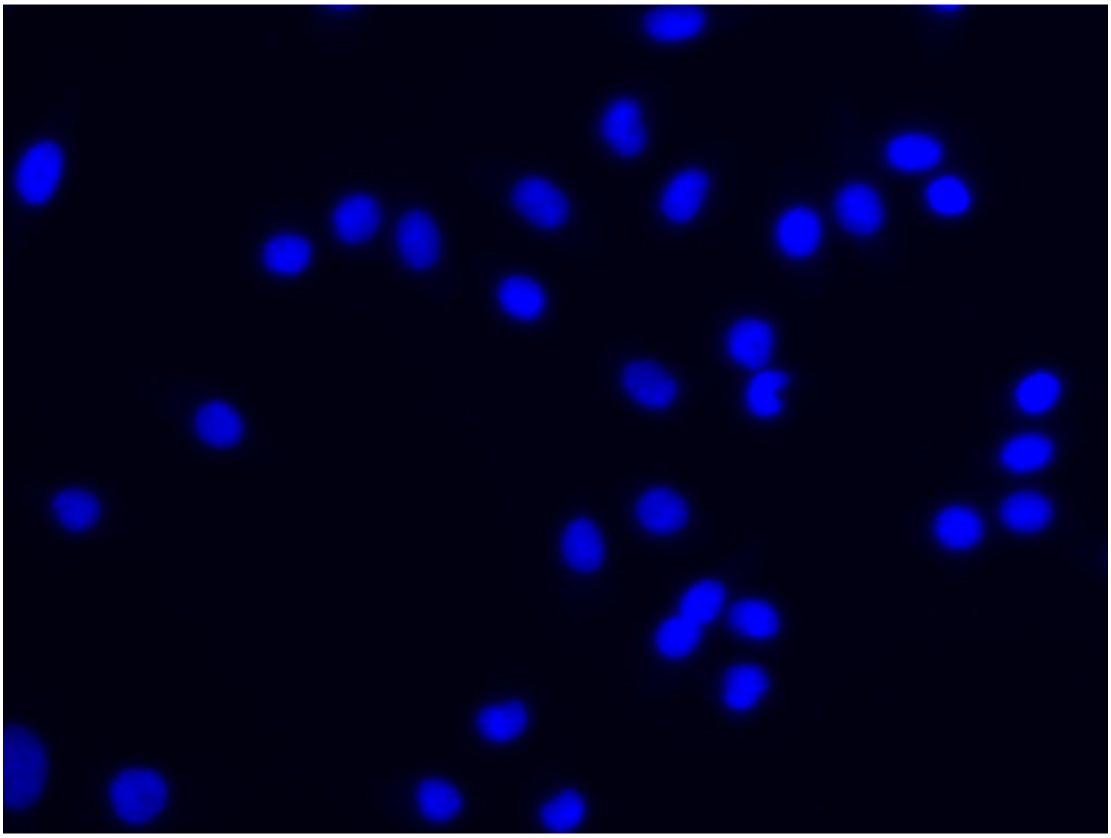

HO8910-SH-CDR1AS+1299

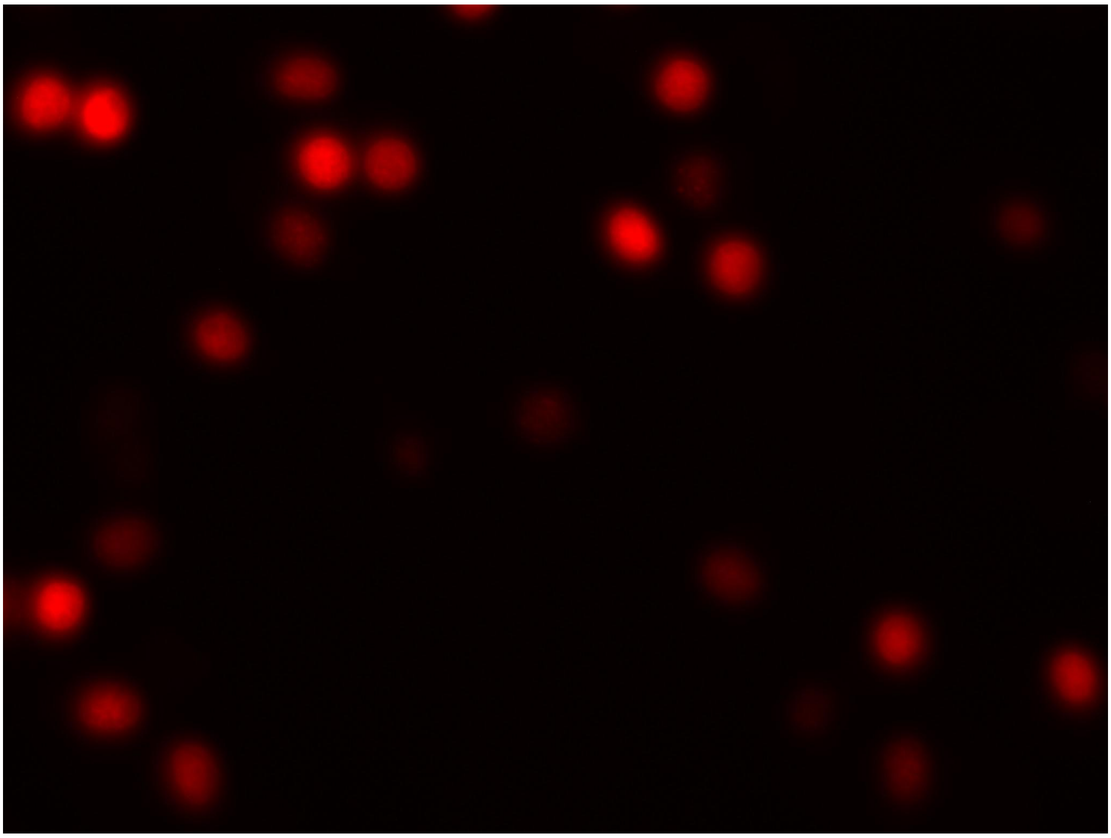

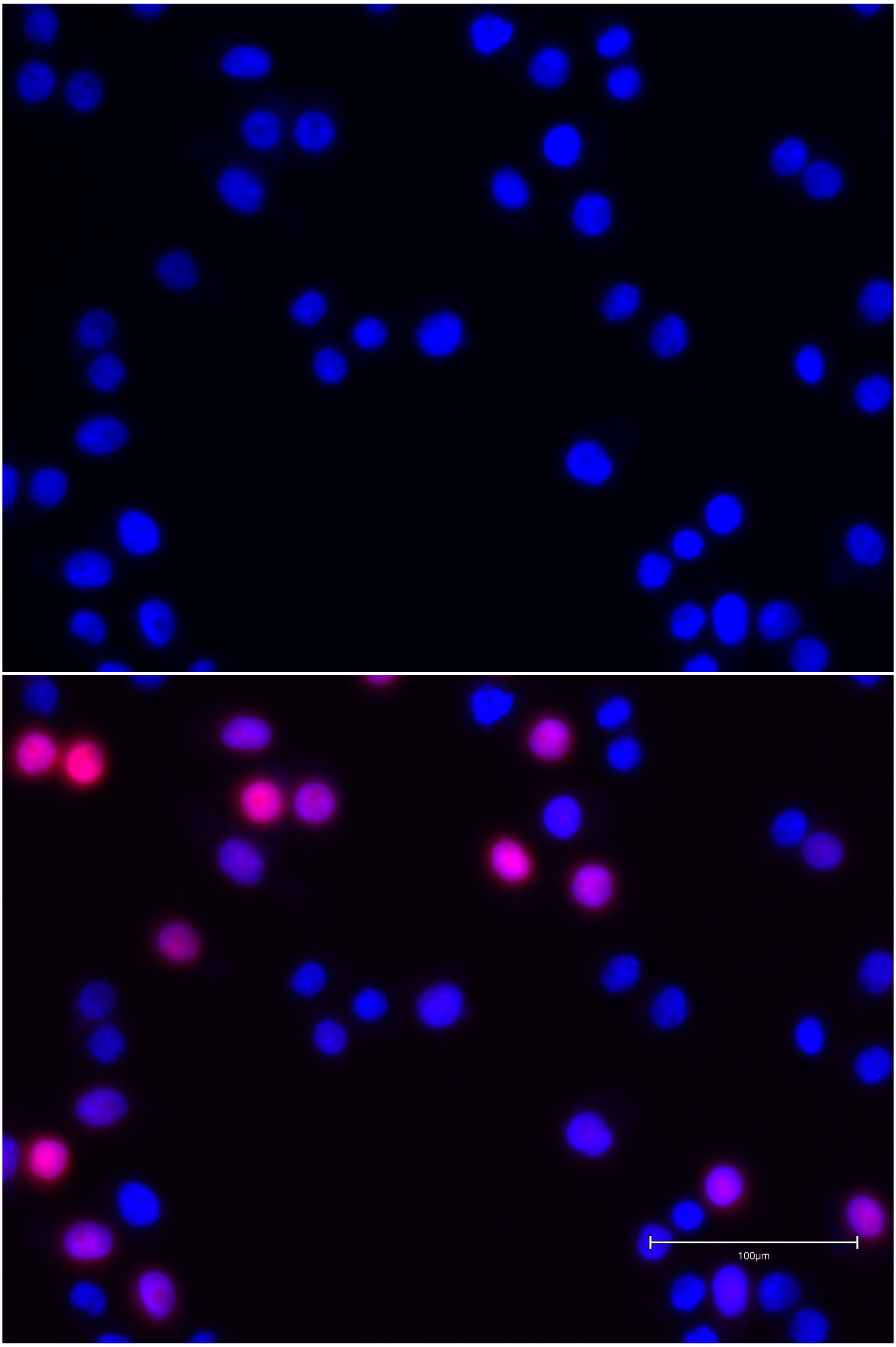

SKOV3-CONTROL+1299

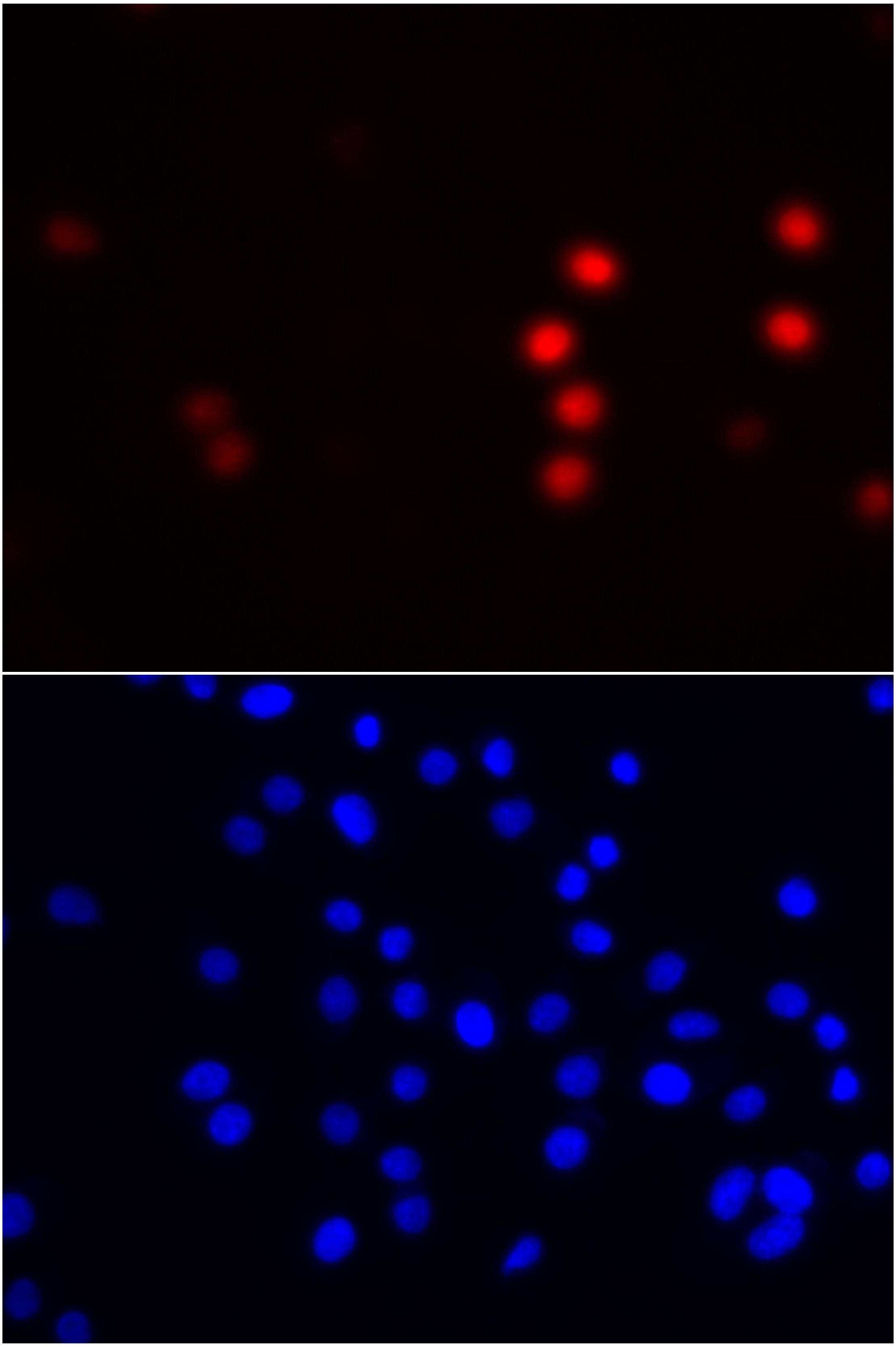

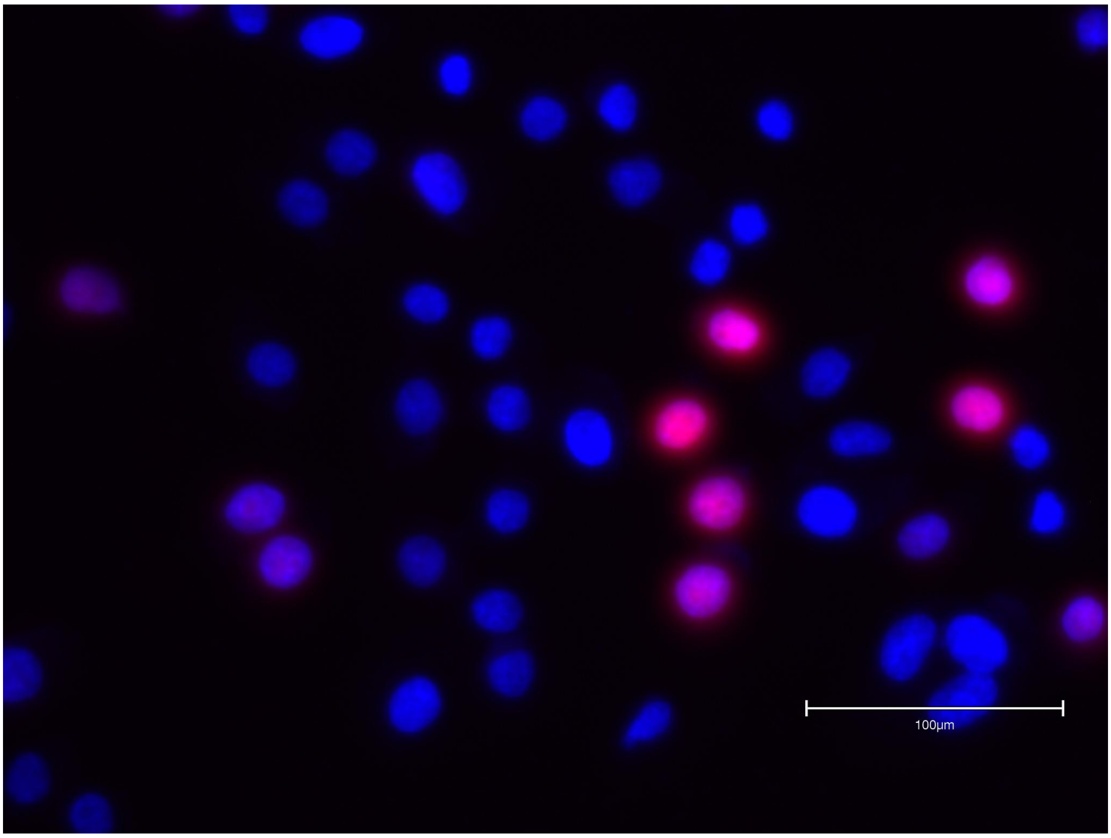

SKOV3-SH-CDR1as+NC

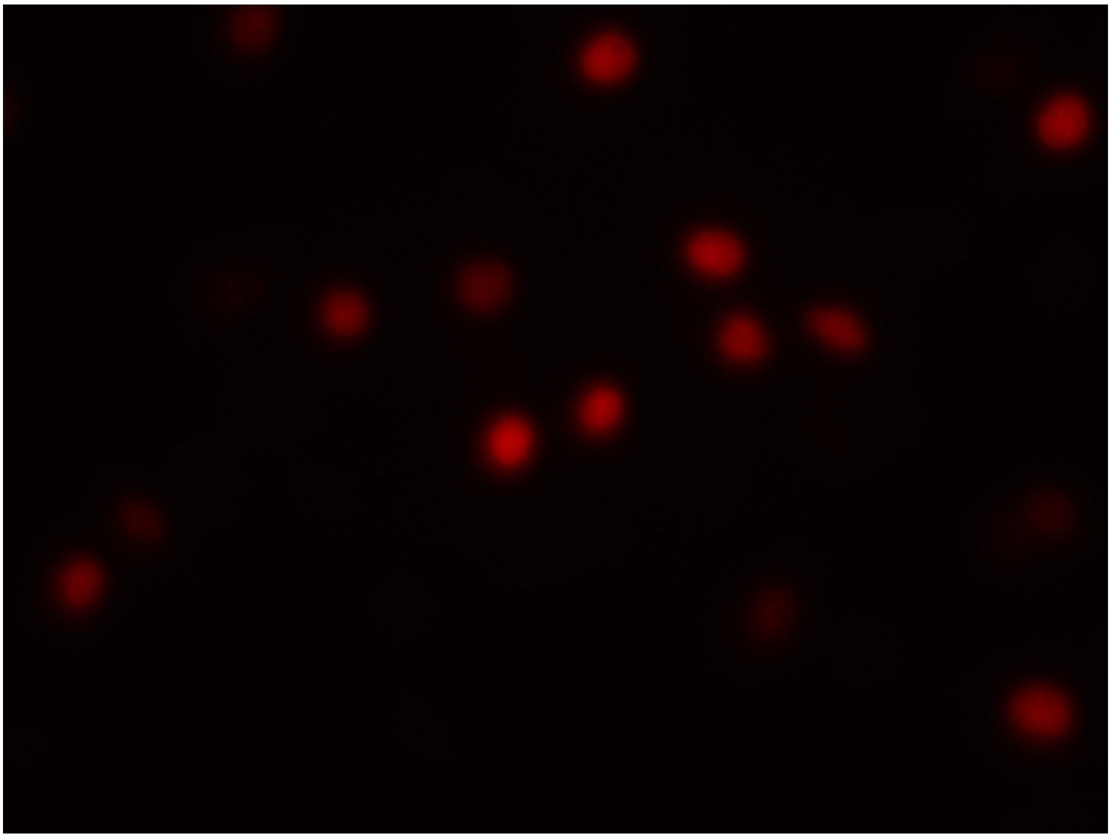

SKOV3-CONTROL+NC

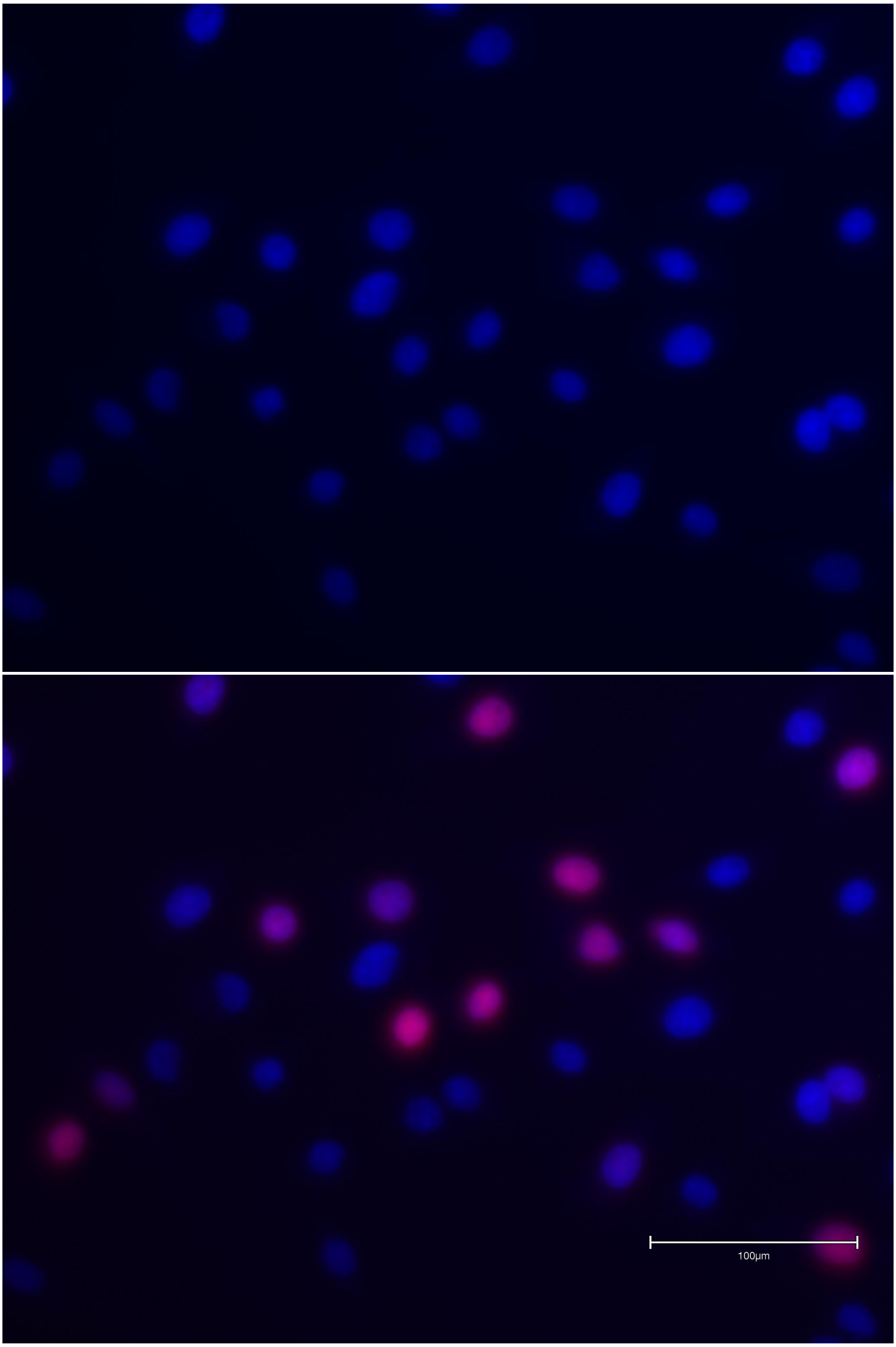

SKOV3-CONTROL+NC

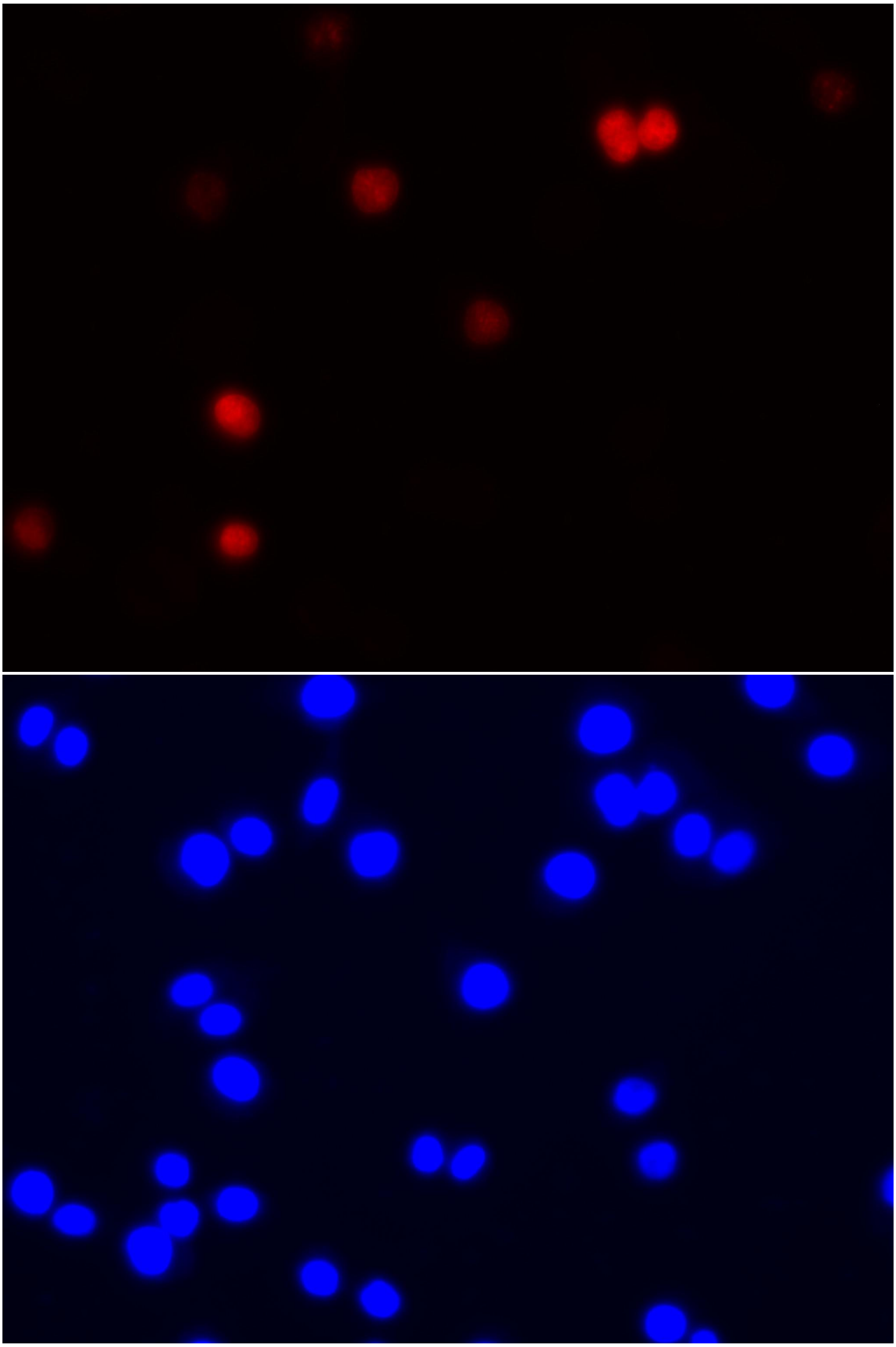

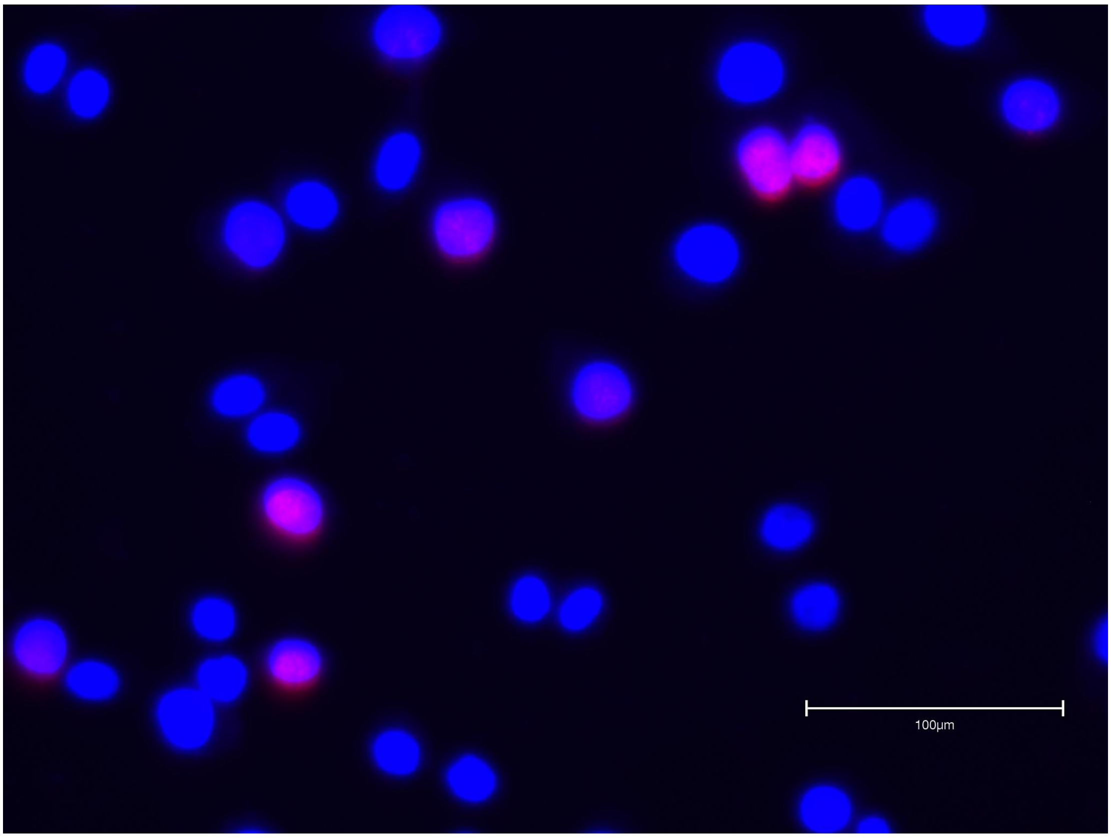

SKOV3-SH-CDR1as+1299

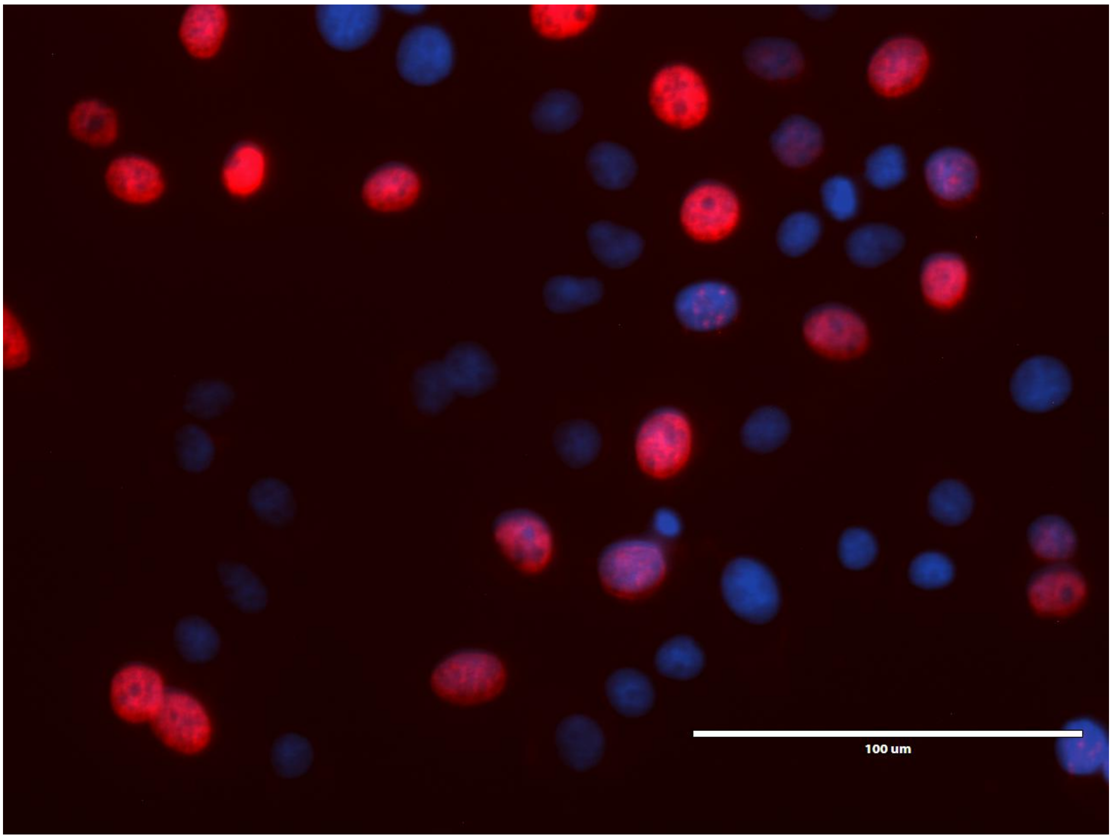

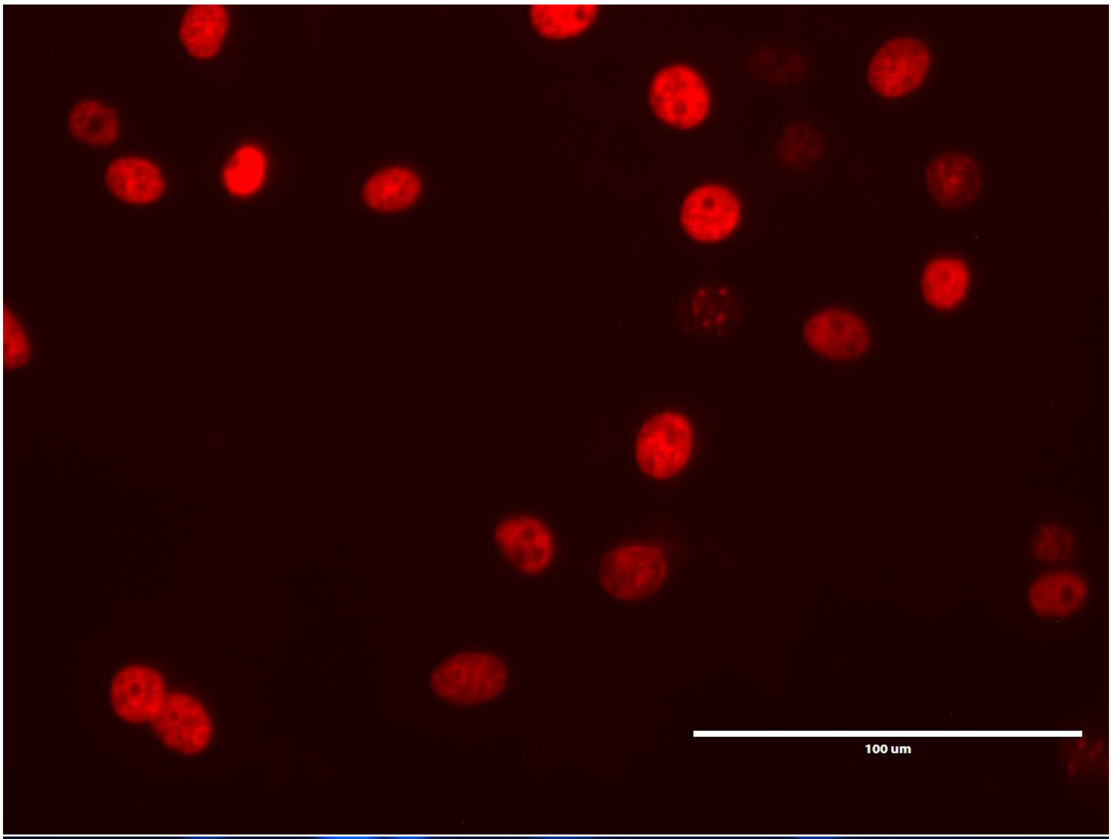
100 um

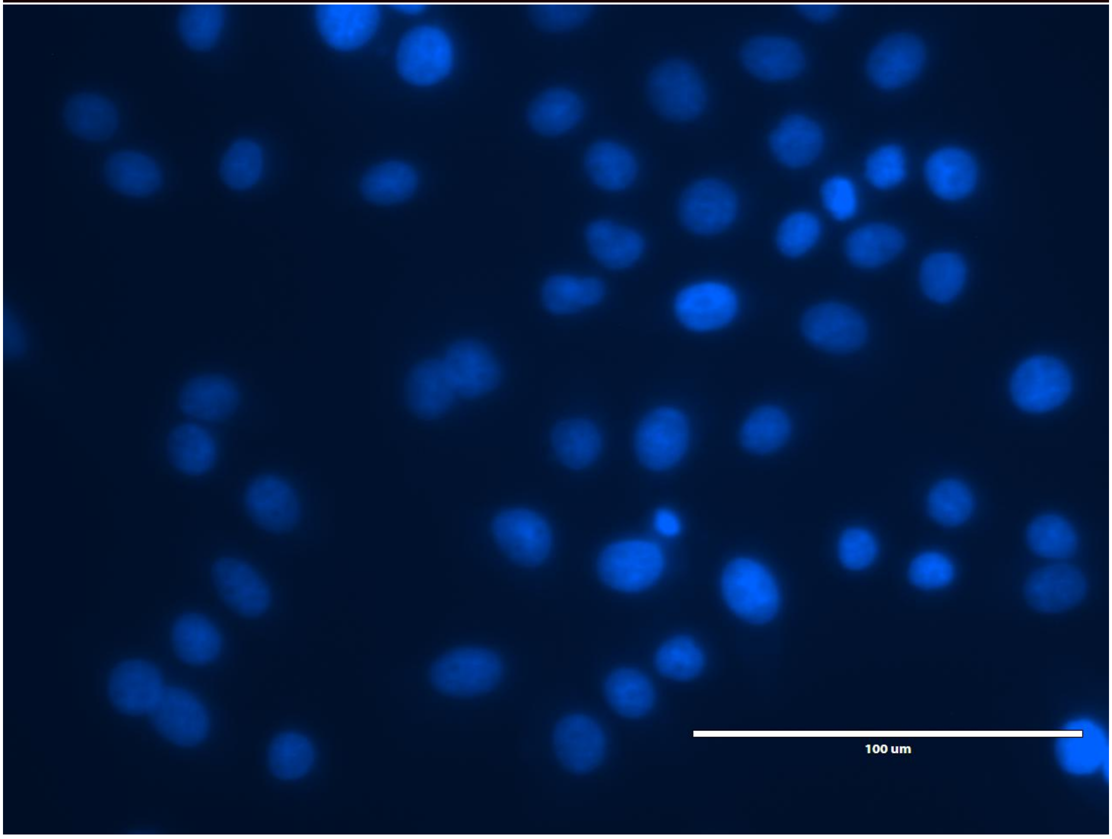
100 um
